# High site-fidelity in common bottlenose dolphins despite low salinity exposure and associated indicators of compromised health

**DOI:** 10.1371/journal.pone.0258031

**Published:** 2021-09-30

**Authors:** Ryan Takeshita, Brian C. Balmer, Francesca Messina, Eric S. Zolman, Len Thomas, Randall S. Wells, Cynthia R. Smith, Teresa K. Rowles, Lori H. Schwacke

**Affiliations:** 1 National Marine Mammal Foundation, San Diego, California, United States of America; 2 Water Institute of the Gulf, Baton Rouge, Louisiana, United States of America; 3 Centre for Research into Ecological and Environmental Modelling, University of St Andrews, St Andrews, United Kingdom; 4 Chicago Zoological Society’s Sarasota Dolphin Research Program, Mote Marine Laboratory, Sarasota, Florida, United States of America; 5 National Oceanic and Atmospheric Administration, National Marine Fisheries Service, Office of Protected Resources, Silver Spring, Maryland, United States of America; Wildlife Conservation Society Canada, CANADA

## Abstract

More than 2,000 common bottlenose dolphins (*Tursiops truncatus*) inhabit the Barataria Bay Estuarine System in Louisiana, USA, a highly productive estuary with variable salinity driven by natural and man-made processes. It was unclear whether dolphins that are long-term residents to specific areas within the basin move in response to fluctuations in salinity, which at times can decline to 0 parts per thousand in portions of the basin. In June 2017, we conducted health assessments and deployed satellite telemetry tags on dolphins in the northern portions of the Barataria Bay Estuarine System Stock area (9 females; 4 males). We analyzed their fine-scale movements relative to modeled salinity trends compared to dolphins tagged near the barrier islands (higher salinity environments) from 2011 to 2017 (37 females; 21 males). Even though we observed different movement patterns among individual dolphins, we found no evidence that tagged dolphins moved coincident with changes in salinity. One tagged dolphin spent at least 35 consecutive days, and 75 days in total, in salinity under 5 parts per thousand. Health assessments took place early in a seasonal period of decreased salinity. Nonetheless, we found an increased prevalence of skin lesions, as well as abnormalities in serum biochemical markers and urine:serum osmolality ratios for dolphins sampled in lower salinity areas. This study provides essential information on the likely behavioral responses of dolphins to changes in salinity (e.g., severe storms or from the proposed Mid-Barataria Sediment Diversion project) and on physiological markers to inform the timing and severity of impacts from low salinity exposure.

## Introduction

**Fig 1:**
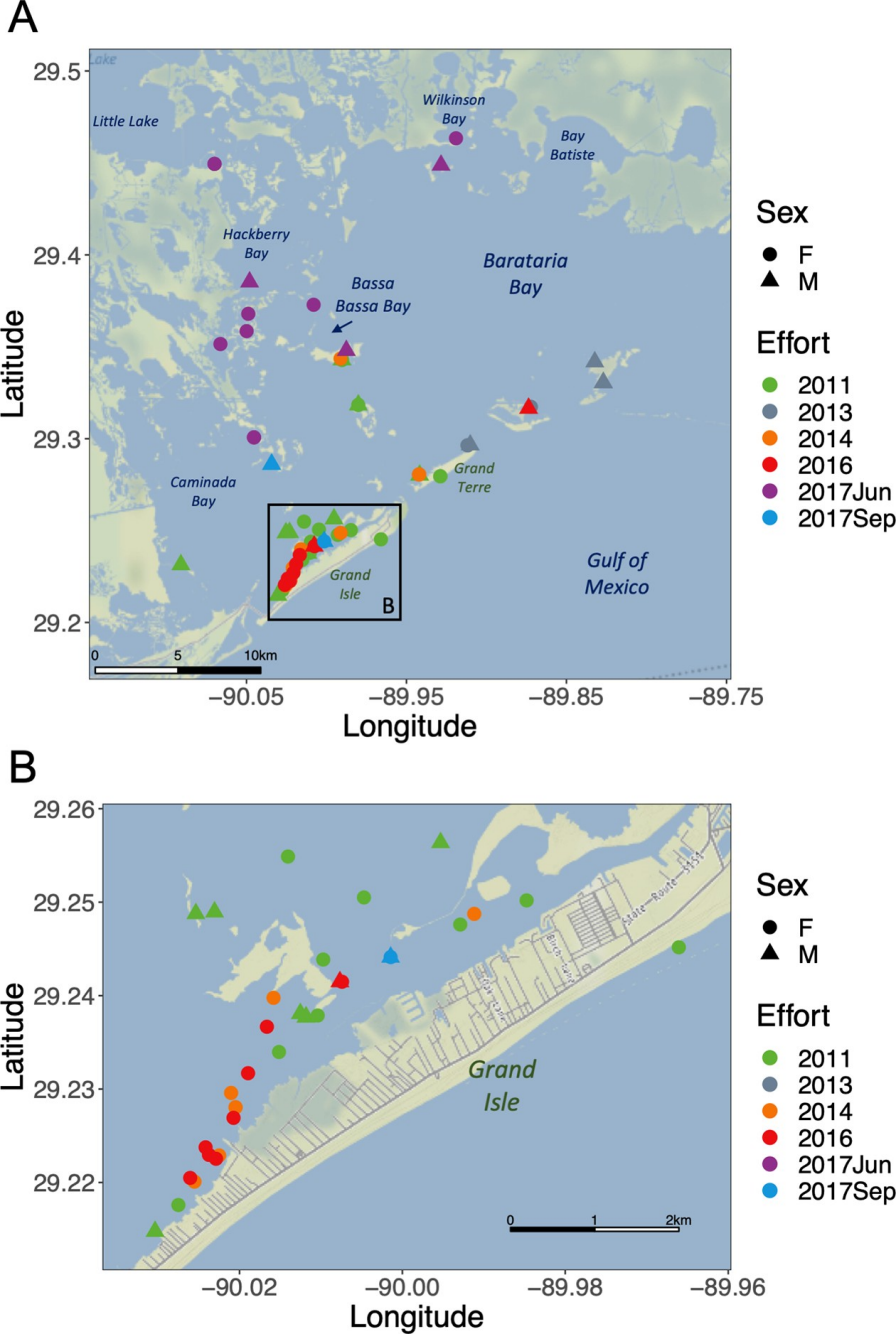
Capture/Release locations for all dolphins with satellite-linked telemetry tags in the Barataria Basin, LA from 2011 to 2017. (A) The June 2017 field effort was designed specifically to investigate dolphins near or north of Bassa Bassa Bay (purple), while the other efforts typically focused on dolphins near the barrier islands, especially Grand Isle (B). Map tiles used with permission from Stamen Design, under a CC BY license. Data by OpenStreetMap, under ODbL.

The Barataria Basin—including Barataria Bay, Caminada Bay, and surrounding coastal marsh habitat south of New Orleans, LA and west of the Mississippi River—is home to approximately 2,000 common bottlenose dolphins (*Tursiops truncatus*) (Fig 1) [1,2]. The ecosystem in the basin ranges from freshwater wetlands at the northern reaches to a marine system on the south side of the barrier islands (e.g., Grand Isle and Grand Terre; the location of all places named within the Barataria Basin are shown in Fig 1) that separate Barataria Bay from the northern Gulf of Mexico (nGOMx), with the majority of the basin representing a gradient of brackish waters subject to daily, seasonal, and/or yearly fluctuations in salinity [3,4].

Although the Barataria Basin Estuarine System (BBES) and the neighboring Terrebonne-Timbalier Bay Estuarine System have been designated as estuaries of national significance, the accumulated effects of levees and channels near the Mississippi River have driven significant changes to the hydrology and habitat of this ecosystem. In combination with the effects of increased development, climate change, tropical disturbances, and disasters (e.g., the *Deepwater Horizon* [DWH] oil spill), the quality and quantity of coastal habitat in the basin is rapidly decreasing [5,6]. As part of a larger effort to combat coastal erosion across Louisiana, resource managers have proposed to divert sediment from the Mississippi River into the Barataria Basin via the Mid-Barataria Sediment Diversion project [7]. To divert the sediment, large volumes of freshwater will be introduced into the Barataria Basin, which may affect estuarine species that depend on aquatic habitats with particular ranges of salinity [4].

In the U.S., marine mammals are managed by individual stock, which is defined as a group of marine mammals of the same species in a common spatial arrangement that interbreed when mature (MMPA 16 U.S.C. 1361 et seq.). While many stocks of common bottlenose dolphins spend their entire lives in marine waters (typically >30 parts per thousand [ppt]), at least some bottlenose dolphins can tolerate lower saline conditions. As an individual’s exposure to low salinity conditions increases in duration and/or severity (closer to 0 ppt), the dolphin can be negatively affected by direct contact of the skin to low saline water, as well as incidental ingestion of low saline water during foraging. Dermal exposure may lead to skin lesions such as color changes and epidermal sloughing, erosions, and/or ulcerations, indicating progressive stages of the skin’s inability to act as a barrier to external conditions [8–14]. In addition, skin may become overgrown with external mats consisting of fungi, algae, and/or bacteria that can penetrate the skin as the impacts of lower saline waters become more severe. Once the skin is compromised, the individual may develop secondary infections and/or net gain of low saline water into the body [13]. Similarly, gastrointestinal integrity may also be compromised, depending on how much water an individual consumes [15], leading to intracellular and extracellular absorption and potential for infections after incidental ingestion of low saline water. Both of these exposure pathways may lead to systemic physiological changes like osmotic imbalance, biochemical aberrations, cellular damage, and the potential for localized/systemic secondary infections or septicemia. Exposure to waters <20 ppt for as little as one day can result in mild serum electrolyte changes [16,17]. Visually observable skin lesions may require exposures <10 ppt for days to weeks, and the lesions/physiological effects of low salinity exposure can resolve if/when the individuals return to higher saline waters [17–21].

In some circumstances, osmotic imbalance, biochemical aberrations, cellular damage, and/or secondary infection can deteriorate from mild to severe, resulting in potentially life-threatening illnesses (e.g., hemolysis, anemia, septicemia/toxemia, and cerebral or pulmonary edema). In 2019, a record-breaking year of precipitation in the watersheds draining to the nGOMx led to large amounts of fresh water flowing into coastal waters, driving salinity levels below 10 ppt for 15–20 weeks [22]. This resulted in a four-fold increase in stranded bottlenose dolphins from Louisiana to the Florida Panhandle, with a high prevalence of freshwater-like skin lesions, and led NOAA to declare an unusual mortality event (UME) [23]. Many of the strandings were in eastern Louisiana, western Mississippi Sound, and adjacent bays/estuaries, coinciding with increased runoff from the Mississippi River and associated spillway openings. In this area, stranded dolphins with freshwater-like lesions were found after 17 days with nearby waters below 5 ppt, and the number of strandings peaked after 40 days with waters below 5 ppt [23].

Like other bay, sound, and estuarine stocks of dolphins in the Southeastern U. S. [reviewed in 2], the BBES Stock shows high, year-round site fidelity to the estuarine waters within the basin, with occasional movements <5 km from the barrier islands into adjacent coastal waters of the nGOMx [24]. Only a few individual dolphins have been identified moving between the Barataria Basin and the neighboring Terrebonne-Timbalier Bay Estuarine System [25]. During summer/fall months from 2011 to 2014, individual BBES dolphins with telemetry tags typically ranged (defined as the mean longest distance between locations across a home range) no more than 22 km for females and 27 km for males while the tags were operational (about four to five months) [24]. This is consistent with extensive photographic-identification and telemetry research documenting that bay, sound, and estuarine dolphins in the Southeastern U.S. typically stay in relatively small usage areas [e.g., 24,26–33], often despite acute or chronic environmental stressors such as low salinity [10,19,34], tropical disturbances [21,35], oil spills and other anthropogenic contaminants [10,34,36–38], and harmful algal blooms [35,39–42]. Due to their high site fidelity, BBES dolphins were exposed to DWH oil in their heavily contaminated habitat and subsequently suffered numerous adverse health effects from oil toxicity (e.g., lung disease, impaired stress response and adrenal dysfunction, immune dysfunction, and poor overall health prognoses), leading to drastic and prolonged elevated reproductive failure rates and mortality rates following the oil spill [43–53]. NOAA declared a UME related to the effects of DWH oiling on cetaceans throughout the nGOMx, including dolphins in the Barataria Basin [51,54].

Most of our understanding of the health and movements of the BBES Stock comes from investigations into the effects of the DWH oil spill on BBES dolphins (where oiling was especially persistent and heavy), with most of the studies focused near the barrier islands, particularly Grand Isle and Grand Terre [44,55,56]. For example, in an effort to extrapolate densities of individuals into an estimate of abundance of BBES dolphins, Hornsby et al. [57] used telemetry data from 2011 to 2014 (first described by Wells et al. [24]) to define dolphin habitat area in the basin based on a salinity model. Similarly, White et al. [58] use the same telemetry dataset in preliminary comparisons of modeled salinity to dolphin locations. Both studies observed that the tagged BBES dolphins spent little time in waters below 7.98 ppt; however, most of the tagged dolphins in the dataset were captured in the southern half of the stock area (where salinities are typically less affected by seasonal fluctuations, since they are closer to the open waters of the nGOMx).

In 2017, we conducted catch-and-release health assessments in the Barataria Basin, with a focus on dolphins found ranging in the northern half of the stock area, where salinities are more influenced by temporal fluctuations of freshwater input. Satellite-telemetry tags were attached to individuals so that we could compare their movement patterns to salinity trends as modeled using the Delft3D Barataria Basin hydrodynamic model [58]. We conducted the health assessments and simultaneously deployed tags during the transition period from the lower salinity spring/summer rainfall runoff period to the higher salinity fall/winter period (typically dominated by influx of waters from the nGOMx). This provided an opportunity to investigate whether dolphins in the northern part of the BBES Stock area move in association with changes in salinity, and with data from the health assessments, to determine if BBES dolphins that spent time in the lower salinity environments of the northern portion of the stock area were more likely to exhibit skin lesions and/or clinicopathological indicators of low salinity exposure.

## Materials and methods

Dolphin catch-and-release efforts were conducted in August 2011, June 2013, June 2014, July 2016, June 2017, and September 2017. All field research with bottlenose dolphins was authorized under NMFS’ Marine Mammal Health and Stranding Response MMPA/ESA Permit #932-1905/MA-009526 and #18786–1. During each catch-and-release effort, the dolphins included in this study were each 1) given a health assessment by veterinarians and 2) affixed with satellite telemetry tags. Data from the 2011, 2013, and 2014 health assessments and satellite telemetry tags were described previously [24,44,48], and information about these dolphins is provided in S1 and S2 Tables. From September 11 to September 22, 2016 and on September 21, 2017, health assessment and tagging activities were focused on the waters near Grand Isle and Grand Terre in the southern portion of the BBES Stock area (Fig 1) and used protocols similar to the previous field efforts [59].

### Dolphin catch-and-release in June 2017

From June 14 to June 23, 2017, our research team temporarily captured dolphins (one to two individuals at a time) in the Barataria Basin with a focus on dolphins found in the northern half of the BBES Stock area (including and north of Bassa Bassa Bay, or approximately 29.34° N), attached satellite-telemetry tags, and collected dolphin health data. We used previously established protocols for the safe capture, handling, health assessment, tag attachment, and release of dolphins, focusing on individuals over two years of age [24,44,48,59]. At each capture location, we recorded geographic and environmental data, including the global positioning system (GPS) coordinates and water depth via a vessel-based GPS unit, as well as water temperature and salinity using a Yellow Springs Instruments (YSI) probe, for comparison with modelled salinity estimates.

### Satellite-telemetry tagging

The tagging equipment and methods we used are consistent with the review of satellite-telemetry tagging of small cetaceans in Balmer et al. [60] and the techniques described in Wells et al. [24]. In 2016, we deployed 10 SPOT-299A tags from Wildlife Computers (Redmond, Washington, USA) with a projected battery life of 280 days at 250 transmissions per day. In 2017, we deployed two types of tags: SPOT-299A and Sirtrack’s (Havelock North, New Zealand) K2F 172C KIWISAT 202B (location only), with a projected battery life of 168 days. Satellite-telemetry tagging of Barataria Bay dolphins between 2011 and 2014 is described by Wells et al. [24]. All tags were coated with Propspeed (Oceanmax, Ltd., Auckland, New Zealand), excluding the saltwater switches, to reduce biofouling. The distance between the center of the attachment point to the trailing edge of the dorsal fin was approximately 35 mm. We programmed each tag to attempt signaling with the Argos satellite system during four, one-hour blocks per day (08:00 to 11:59 Central Daylight Time) in order to maximize the number and quality of the transmissions. Although on occasion we tagged two dolphins that were caught at the same time, after assessing the telemetry data, it was clear that either 1) one of the tags did not transmit enough times to be included in our data set or 2) that the individuals were likely only associated for a brief time (due to the fission-fusion nature of bay, sound, and estuary dolphin social structure) rather than mother-calf pairs or bonded adult male pairs [e.g., 61]. Thus, we treated all of the tags as independent samples.

### Health assessment

Comprehensive veterinary health assessments of each dolphin have been previously described [44,48,59] and included a physical examination, morphometric data, skin and blubber biopsy, urinalysis (when available), pulmonary and reproductive ultrasound examinations, and blood sample collection for complete biochemistry, hematology, and osmolality analysis. The types of samples/analyses for each individual dolphin varied depending on the sampling priority for each field effort’s study design, timing constraints due to weather and/or sample processing and shipping requirements, and how well the dolphin tolerated sampling procedures.

For each dolphin in June and September 2017, veterinarians conducted a skin assessment using a standardized form to document the location and description of each lesion type (Supplemental Materials). Visual assessment of freshwater-like lesions was typically characterized by hypopigmented, circular and/or irregular, multifocal to coalescing lesions and may or may not have had associated ulcerations and were consistent with freshwater lesions described in other studies [13,14]. Two experienced researchers (LS and CS) reviewed photographs of each dolphin to confirm the presence or absence of freshwater-like lesions.

To evaluate the hematology and serum chemistry data, we analyzed panels of related analytes representing pathologic processes (e.g., inflammation) or organ systems (e.g., hepatobiliary) as described previously [44]. We defined the low salinity exposure cohort as those dolphins with at least two days of low salinity (<5 ppt) exposure in the week leading up to the health assessments, based on the median modeled salinity of each individual’s potential ranging area (PRA, see below) prior to their capture/release date, while the remaining dolphins were considered the reference group. We calculated prevalence of abnormal health panels and estimated 95% confidence intervals (CIs) using the Agresti-Coull method. We estimated relative risk of an abnormality in each health panel for the low salinity exposure cohort versus the reference cohort using the *epitools* R package. We calculated and present p-values for relative risk (using the median-unbiased method), but we did not compare to a threshold value for statistical significance. We instead present 95% confidence intervals for the relative risk to represent uncertainty in the estimates.

### Dolphin movements relative to modeled salinity

We compared dolphin telemetry data to modeled daily salinity fields across the BBES Stock area. Salinity estimates were generated by the Delft3D-based hydrodynamic model as described in White et al. [58]. In brief, the Delft3D model was calibrated and validated by using a variety of field observations. Water level, velocity, salinity, and temperature were calibrated by comparing the model output with USGS, CRMS, and NOAA data. Historical simulations were performed to estimate the salinity conditions of Barataria Basin from 2011 to 2017. Field observations were used to impose the model boundary conditions (i.e., riverine discharge, tides, etc.) and atmospheric forces for those specific years. The model uses a triangular grid with 375 m resolution in the BBES Stock area (although a small portion of the stock area, close to the Mississippi River, has a 125 m resolution). The model is a 2D-depth average and therefore the salinity estimates are averaged over depth. Most of the basin is relatively shallow (~2 m on average) [2], however deeper water can be found near passes between the barrier islands and in dredged shipping channels.

We used publicly available packages for the statistical software R (version 4.0.0) [62] and packages *tidyverse*, *PropCIs*, *ggmap*, *sf*, *stars*, *raster*, *akima*, *and adehabitatHR* to conduct our GIS and statistical analyses. We interpolated the Delft3D output onto a grid with 375 m square cells and removed pixels that consisted entirely of land (based on 2018 shoreline maps, as described below). We used these daily rasters to 1) identify the salinity for each dolphin’s telemetry location/timepoint, 2) calculate the daily median salinity across the Barataria Basin (as defined above), and 3) calculate the daily median salinity in each dolphin’s PRA. When we summarize these daily median salinity values across multiple individuals, we report the mean of the daily median salinity values. Some of the telemetry locations (approximately 6.2%) and portions of the PRAs were outside the modeled salinity dataset’s spatial extent or in locations that the model considered land. These telemetry locations were removed from the salinity analyses. To compare the Delft3D model salinity estimates with the field-collected salinity measurements, we conducted a simple linear regression comparing the difference between the model estimate and the field measurement versus the field measurement alone.

For the purposes of our analyses, we selected 5 ppt as the threshold for low salinity conditions. However, other studies have shown that dolphins exposed to salinities higher than 5 ppt suffered adverse health effects consistent with low salinity exposure [e.g., 16,17]. To calculate the number of consecutive and cumulative days each dolphin spent under 5 ppt, we generated daily mean salinity exposure levels (in ppt) for each individual based on their telemetry locations. When an individual dolphin’s satellite-telemetry tag transmitted more than once daily, we selected one random location from the highest quality locations (Argos Quality 1, 2, and 3) in each six-hour window (see section below), then we averaged the predicted salinity values for those locations to get a single mean predicted salinity value for each dolphin on each day. Because not every tag transmitted a high-quality location every day, we allowed for gaps of no more than 48 hours (i.e., we assumed that dolphins transmitting from low-salinity locations two times within 48 hours most likely did not move into higher-salinity water between those two transmissions, but we allowed for the possibility that a dolphin could have moved into higher-salinity water between two transmissions that were more than 48 hours apart).

### Estimating individual dolphin ranging areas

Kernel density estimates (KDEs) and percent volume contours (PVCs) can be used to answer a variety of questions about how animals use space, including describing home ranges, foraging areas, territory, habitat, etc. [63]. The parameters and techniques underpinning these calculations need to be chosen carefully depending on the study questions [reviewed in 64,65]. Generally, the methods are useful for comparing animals within a study, but should be used cautiously to compare across studies. We were particularly interested in characterizing dolphin movements in relation to changes in salinity within each dolphin’s range. Therefore, as discussed below, we selected parameters that likely overestimate the size of each dolphin’s 95% PVC to avoid limiting the potential salinity range that each dolphin could access. By calculating larger PVCs, we allow for a wider range of salinities to test if/how an individual dolphin might move to respond to fluctuations in salinity.

We select the term “potential ranging area” (PRA) to describe the waters that each dolphin might move within (and the salinities in that area), based on their telemetry locations. PRA sizes were calculated, using the *adhabitatHR* package in R [66], using the 95% PVC of fixed, bivariate normal KDEs on a 200 x 200 pixel grid. We included telemetry data locations with error <1,500 m (Argos Quality 1, 2, and 3). To avoid autocorrelation due to several locations in one time/place (and because the interval between transmissions was not constant) [67], we randomly selected from the highest quality locations in any given six-hour period (0:00–6:00, 6:00–12:00, 12:00–18:00, and 18:00–24:00 each day). Our final dataset consisted of 7,454 locations. Across our study time period, the median number of highest quality locations in each transmission window was 1 location, with a maximum of 6 locations in one transmission window. Although we calculated 95% PVCs for any individual dolphin with ≥10 such locations, we limited summary statistics (pooling the telemetry data across individuals) to only include individuals with >50 such locations. The resulting dataset was not normally distributed, based on a Shapiro-Wilk test, and so we report the median and the inter-quartile range (IQR) of dolphins grouped based on their transmitted locations. “Barrier island-associated dolphins” were mostly restricted to the waters near the barrier islands, while “interior dolphins” transmitted from locations north of the barrier islands (but may also have visited the barrier islands).

We use the *ad hoc* approach described in Kie et al. [68], where the bandwidth parameter is iteratively reduced from the reference bandwidth (*h*_*re*f_) until one finds a minimum value for *h* where the 95% PVC is a single, contiguous polygon (*h*_*ah*_). We did not allow *h* to be greater than *h*_*re*f_, thus some 95% PVCs were fragmented. The *h*_*ah*_ used for each individual is reported in S2 Table. Although using 10–20 locations to calculate utilization domains may underestimate the PRA for these individuals, our choice to use the *ad hoc* approach in selecting a bandwidth will partially offset this problem, and will allow us to generate reasonable estimates of salinities in waters used by these dolphins (i.e., we do not necessarily need a perfect estimate of a dolphin’s home range, but a reasonable representation of the salinities they were exposed to). We are also only comparing salinities and locations for the time periods when the tags were transmitting.

For the purposes of our study, we did not take physical barriers into account when performing the KDE and calculating the 95% PVC, as 1) the resolution of the salinity model (375 m) reduces the complexity of the land:water interface, 2) the Delft3D model does not generate salinity estimates at locations with land, and 3) potential dolphin movements in response to daily salinity changes (the time resolution of the salinity model dataset was daily) would likely not be hindered by islands within the basin. For example, over the course of 24 hours, a dolphin could circumnavigate Grand Isle—the largest island within the geographic extent of our modeled salinity dataset [24,69]. However, to calculate the size of each PRA, we clipped the 95% PVCs for each individual to exclude land, based on a digitization of the 2018 Barataria Basin coastline generated from a composite of the U.S. Geological Survey (USGS) National Hydrographic Dataset, U.S. Fish and Wildlife Service, National Wetlands Inventory, USGS Wetlands, and the National Landcover Dataset. For simplicity, we use unclipped versions of each contour in the figures presented here. Base map tiles are by Stamen Design, with permission to publish under CC BY 4.0. Data from OpenStreetMap and OpenStreetMap Foundation, which is made available under the Open Database License.

We compared the short-term, fine-scale PRAs to long-term photographic-identification data collected during small-vessel surveys during 2010 to 2019 in the Barataria Basin. These sighting histories included the date, time, and location of the observed dolphins, as well as an identification of the individual, based on dorsal fin matching and/or freeze brands from health assessments. A description and full list of these sightings is provided in the Supplemental Materials. We compared these sightings (n = 1,262 for the 70 dolphins in our satellite-telemetry dataset) to the PRAs defined in our analyses of the 70 tagged dolphins (Supplemental Materials).

## Results

### Field efforts and satellite-telemetry tagging

In June 2017, we carried out six days of catch-and-release activities in the Barataria Basin, mostly north of Bassa Bassa Bay (approximately 29.34° N; Fig 1), including 13 dolphins (9 females and 4 males; S1 Table). We suspended operations for three days due to Tropical Storm Cindy. Salinity at the capture locations ranged from 0.7 to 8.3 ppt for the five days prior to Tropical Storm Cindy. Salinity was 6.2 and 10.2 ppt at the two capture locations on June 23, the day after the storm. In July 2016 and September 2017, our research team assessed BBES dolphins near Grand Isle. During 2016, we conducted seven days of catch-and-release activities (Fig 1), including tagging and assessing ten dolphins (8 females and 2 males). In September 2017, we assessed 22 dolphins (12 females, 10 males) over four days. We caught and released two of these dolphins in northeast Caminada Bay and two dolphins just north of Grand Isle, and fitted these four individuals (1 female, 3 males) with satellite-telemetry tags. Salinity at the July 2016 capture locations ranged from 14.6–27.6 ppt; salinity at the September 2017 capture locations ranged from 17.7–20.3 ppt (S1 Table). Across the three field efforts, dolphins ranged from 185–274 cm in length, including all age classes except dependent calves.

Using only the location data included in our study (Argos Quality 1, 2, and 3), SPOT-299A tags deployed in 2016 (n = 10) transmitted locations on average 85 times (range 16–167) over the course of 62 days (range 14–104). In 2017, SPOT-299A tags (n = 11) transmitted locations on average 100 times (range 47–181) over 107 days (range 48–182), while KS202 tags (n = 6) transmitted an average of 15 locations (range 3–24) over 65 days (range 12–132) (S1 Table).

**Fig 2:**
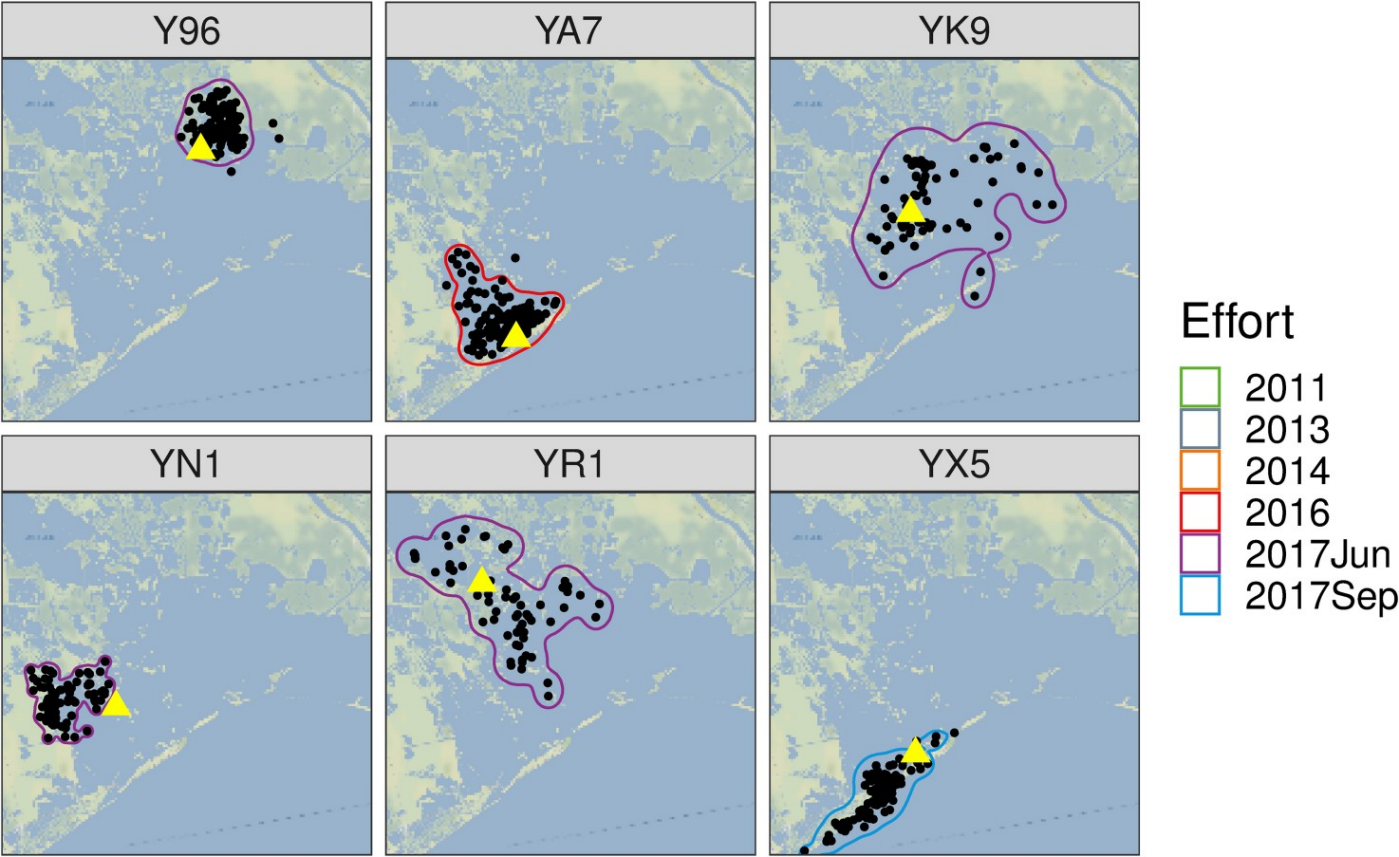
Examples of individual dolphins’ telemetry locations and potential ranging areas from the 2016 and 2017 field efforts. While some dolphins maintained relatively small potential ranging areas throughout the lifetime of their tags (e.g., Y96, YA7, YN1, and YX5), other dolphins showed extended movements within the interior of the basin (e.g., YK9 and YR1). Yellow triangles denote tagging locations. Map tiles used with permission from Stamen Design, under a CC BY license. Data by OpenStreetMap, under ODbL.

While dolphins are found across the study area, the tagging from 2011–2016 and in September 2017 was focused mainly on dolphins near the barrier islands or in the western part of the Barataria Basin. Only 6 out of 58 dolphins (10.3%) had telemetry locations with a mean latitude near Bassa Bassa Bay or further north (29.34° N; S2 Table). In June 2017, 10 of the 12 dolphins (83.3%) had telemetry locations with a mean latitude north of Bassa Bassa Bay (S2 Table). Dolphins tagged in June 2017 were detected as far northwest as Little Lake and in the northern wetlands around Wilkinson Bay and Bay Batiste. Most locations were near wetland edge habitat, however some dolphins moved into the more open waters in the middle of Barataria Bay proper (e.g., YK9 in Fig 2). Across the entire 2011 to 2017 group of tagged dolphins, there was a very strong linear relationship between the latitude at a dolphin’s capture location and the mean latitude of each respective dolphin’s transmitted locations (*R*^*2*^ = 0.76; *F*(1,68) = 217.3; *p* < 0.001).

### Potential ranging areas (PRAs)

While our study questions did not specifically rely on the absolute size of each individual’s PRA, it is informative to make some comparisons within our dataset. For dolphins with at least 50 transmissions in our filtered dataset, there was not a strong linear relationship between the tag lifetime and the PRA size (S1A Fig: *R*^*2*^ = 0.06; *F*(1,55) = 3.5; *p* = 0.068) and tag lifetimes were similar between males and females (S1B Fig: Wilcoxon test *p* = 0.16; S3 Table). From 2011 to 2017, barrier-island associated dolphins (e.g., YX5 in Fig 2) typically remained close to the islands with a median PRA of 58 km^2^ (inter-quartile range [IQR]: 46–78) for females (n = 20) and 103 km^2^ (IQR: 68–115) for males (n = 9) (see S2 Table for the designation of each individual’s location group). Female, interior dolphins with at least 50 transmissions (n = 15) had a median PRA of 187 km^2^ (IQR: 148–217), while male, interior dolphins (n = 13) had a median area of 175 km^2^ (137–258). Although dolphins that spent most of their time in the interior were more likely to have larger PRAs (e.g., YR1 and YK9 in Fig 2; S2 Fig: Wilcoxon test *p* < 0.001), some dolphins tagged in the interior of the basin (approximately the lowest quartile; e.g., Y96) had smaller PRAs similar to the barrier island dolphins (e.g., YA7 and YN1 in Fig 2). For example, Y96 maintained a relatively small PRA (53 km^2^ based on 125 telemetry locations over 126 days) near Wilkinson Bayou (Fig 2). Many of the dolphins tagged in the interior of the basin either moved within or near the wetlands on the western side of the Barataria Basin (similar to the West ranging pattern in Wells et al. [24]), but five dolphins (all tagged in June 2017) generally stayed north of Bassa Bassa Bay and were more likely to move along the northern boundary of Barataria Bay proper (e.g., YR1 and Y96 in Fig 2; see Supplemental Materials for maps of all individuals’ tagging location, telemetry locations, and PRA).

Our analysis of photographic-identification data found that of the tagged dolphins, 63 were sighted in more than one year and six dolphins were sighted in all ten years (2020–2019); the median number of years each dolphin was sighted was six (Supplemental Materials). Cumulatively, 1,174 (93.0%) of the sightings were within each dolphin’s respective PRA. For the sightings outside of the respective PRAs, 78.4% (n = 69) were within 3 km of the respective PRA boundary. Of the tagged dolphins with more than one year of sighting history, only two were not seen within the PRA in multiple years: both of these dolphins (Y37 seen five times and YN1 seen four times) were sighted in only two years, with only one sighting year outside the PRA, and the sightings were each 0.9 km from the respective PRA boundary.

### Dolphin locations and movements relative to salinity

Based on the Delft3D salinity estimates, June 2017 tagging and health assessment activities were conducted during a period when the basin was about to reach its minimum median salinity level for the year (6.3 ppt on June 21; S3 Fig). By the September 2017 field effort, the median basin-wide salinity was 16.7 ppt (on September 21), and the maximum basin-wide median salinity would reach 27.3 ppt on December 23. Other years from 2011 to 2016 generally follow a similar trend, with a peak in salinity in December/January and a nadir in salinity in June/July.

There was a range of ±8 ppt in the differences between the salinity measurements taken in the field during the tagging and health assessment activities (data collected from 2013 to 2017) compared to the Delft3D model estimates for the same dates and locations (S4 Fig). The Delft3D model tended to underestimate salinities near the barrier islands (higher saline waters) and overestimate salinities in the northern part of the study area (lower saline waters), when compared to the salinity measurements taken during the field activities (*β* = -0.42; *R*^*2*^ = 0.63; *F*(1,68) = 117.4; *p* < 0.001) (S5 Fig). In the most northern catch-and-release locations (dolphins Y96, YN9, YR1, and YR3), field-measured salinity was between 0.7 and 2.5 ppt, but the Delft3D model estimated salinity between 4.3 and 4.7 for the same dates/locations. For captures in the highest salinity waters (dolphins Y71, YA7, YF1, and YJ1), field-measured salinity ranged from 23.7 to 27.6 ppt, but the Delft3D model salinities for these dates/locations ranged from 18.4 to 20.9 ppt.

**Fig 3:**
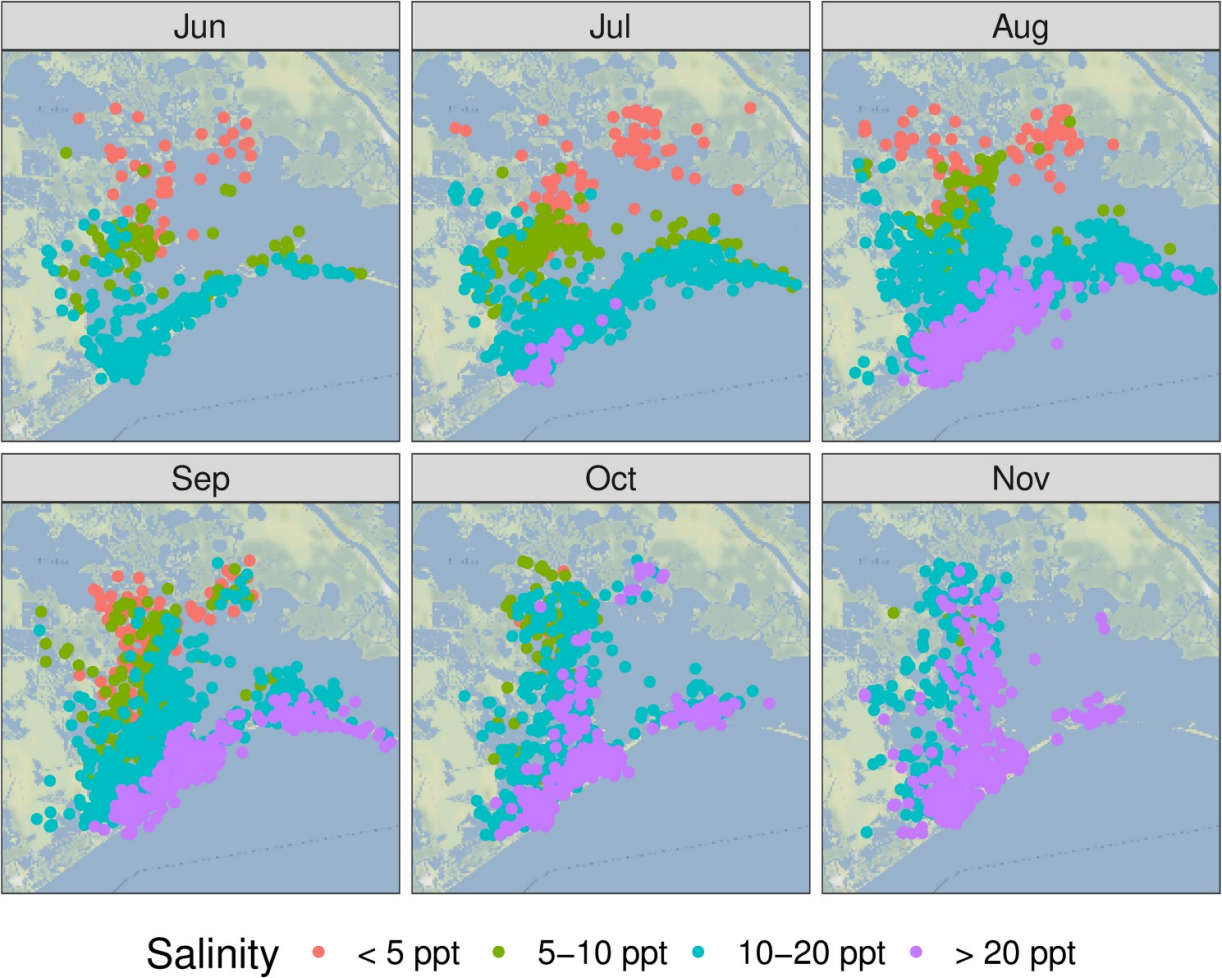
Estimated salinity at telemetry locations/timepoints from 2011–2017. We binned the Delft3D modeled salinity for each telemetry transmission and plotted transmission locations for all individuals. Dolphins at locations with model estimates with low salinities (e.g., < 5 ppt [parts per thousand]) tend to be in the northern part of the basin during the summer months. Map tiles used with permission from Stamen Design, under a CC BY license. Data by OpenStreetMap, under ODbL.

Salinities at dolphin telemetry locations were estimated to be below 5 ppt only in the northern part of the study area mostly between June and October in 2011, 2014, and in 2017 (Figs 3 and S6). Dolphins tagged in June 2017 were located in waters with salinity estimates averaging 7.7 ± 2.9 ppt (± standard deviation), while dolphins from other field efforts ranged from 15.3 ± 2.8 ppt to 23.9 ± 4.3 ppt (Table 1). Based on the estimated salinities at each telemetry location, 15 dolphins experienced at least two days in waters less than 5 ppt (Table 2). Most of these dolphins were tagged in June 2017, but five dolphins tagged in 2011 and one dolphin in 2014 experienced at least 2 days under 5 ppt while their tags were transmitting.

**Table 1:** Estimated mean salinity for dolphin telemetry locations by field effort. ppt = parts per thousand.

Field effort	# of dolphins	Mean salinity at telemetry locations (ppt)	Standard deviation (ppt)
Aug 2011	25	17.7	3.4
Jun 2013	8	15.7	1.5
Jun 2014	11	15.3	2.8
Jul 2016	10	16	1.7
Jun 2017	12	7.7	2.9
Sep 2017	4	24.1	4.3

**Table 2:** The number of cumulative and consecutive days under 5 ppt for each dolphin based on telemetry locations and modeled salinity estimates.

FB	Field effort	Sex	Mean salinity at telemetry locations (ppt)	Days below 5 ppt	Total days with transmissions	% of transmission days under 5 ppt	Maximum consecutive days under 5 ppt
Y96	Jun 2017	M	6.9	72	126	57	35
YR1	Jun 2017	F	3.7	52	95	55	12
YK9	Jun 2017	F	6.2	40	108	37	6
Y94	Jun 2017	M	7.1	16	56	29	5
Y25	Aug 2011	F	11.7	13	160	8	5
Y98	Jun 2017	M	9.5	13	88	15	2
YN9	Jun 2017	F	5.5	13	129	10	3
Y14	Aug 2011	M	11.5	11	208	5	3
Y27	Aug 2011	F	11.9	10	218	5	3
Y37	Aug 2011	F	11.7	10	162	6	3
YN5	Jun 2017	F	9.5	9	111	8	3
YR5	Jun 2017	F	8.9	5	77	6	1
Y22	Aug 2011	M	14.9	4	180	2	3
Y97	Jun 2014	F	8.3	4	145	3	2
YR3	Jun 2017	F	2.9	4	62	6	1

To be considered consecutive, transmissions had to be within 48 hours—i.e., we assumed that dolphins that transmitted twice with 48 hours did not visit waters >5 ppt during that 48 hour gap, but that transmissions more than 48 hours apart allowed for the dolphin to move into waters >5 ppt (parts per thousand).

**Fig 4:**
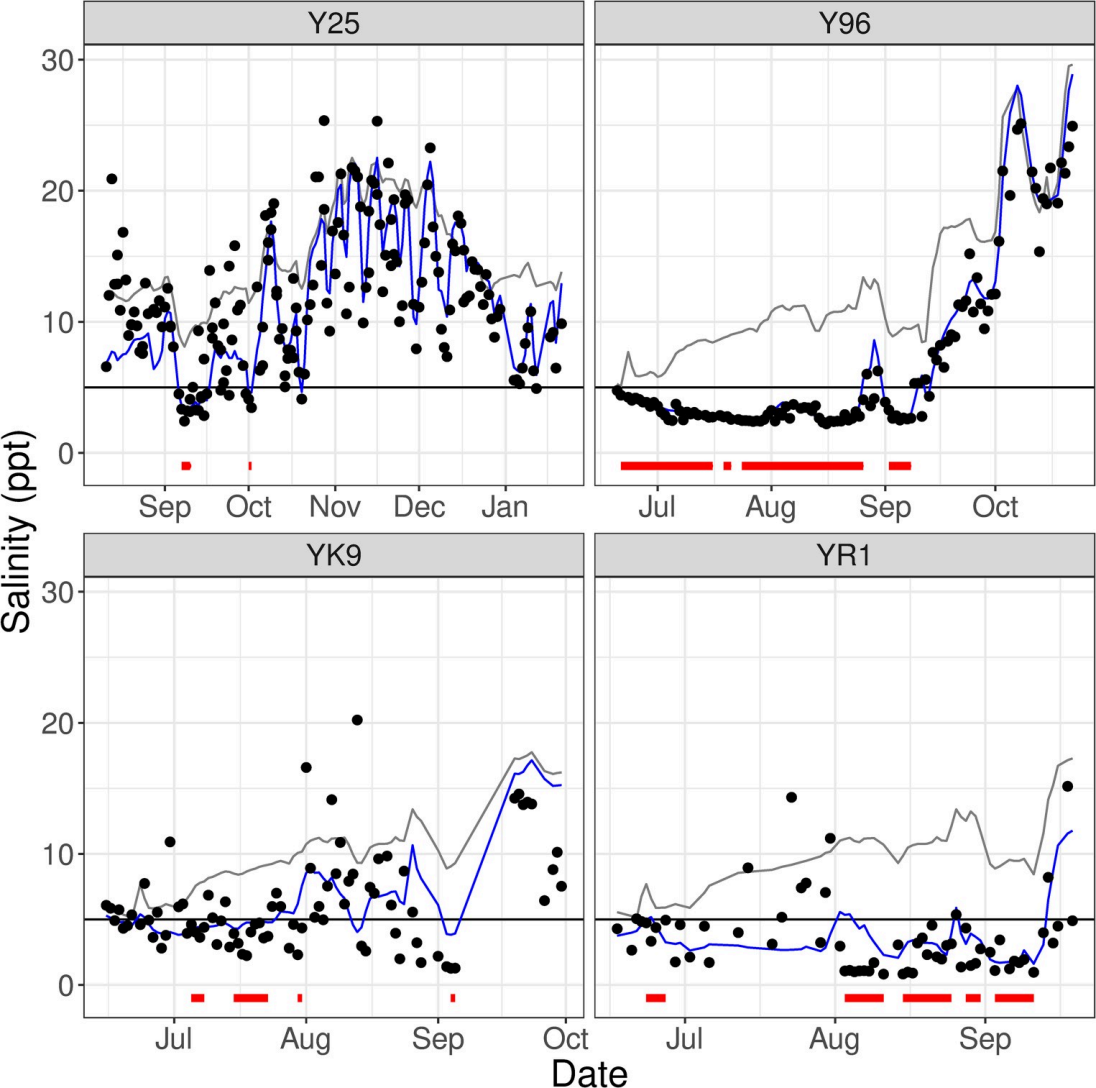
Predicted salinity exposures over time for dolphins in waters less than 5 parts per thousand (ppt) for at least five consecutive days. We compared each dolphin’s salinity exposure (based on their telemetry locations; black dots) to the median salinity within their potential ranging area (PRA) (blue line) and the median salinity across the Barataria Basin study area (grey line). The range and trend of dolphins’ salinity exposures was typically more similar to their potential ranging area than the overall median basin salinity, and several dolphins spent most of the time while they were tagged in waters <5 ppt. Each dolphin in this subset spent one or more periods of at least five consecutive days in waters <5 ppt (red bars).

In total, dolphins tagged in June 2017 spent 224 out of the 852 days (26%) when tags transmitted locations in waters with <5 ppt salinity (Table 2). Two dolphins, Y96 and YR1, spent at least half of the transmission days in waters <5 ppt. Y96 spent at least 72 days (of the 126 days with transmissions) in waters under 5 ppt, including three stretches of consecutive days longer than one week (at least 23, 35, then 8 consecutive days <5 ppt; Fig 4). Four other dolphins, three from the June 2017 field effort and one from the 2011 field effort, were detected in waters <5 ppt for at least five consecutive days.

**Fig 5:**
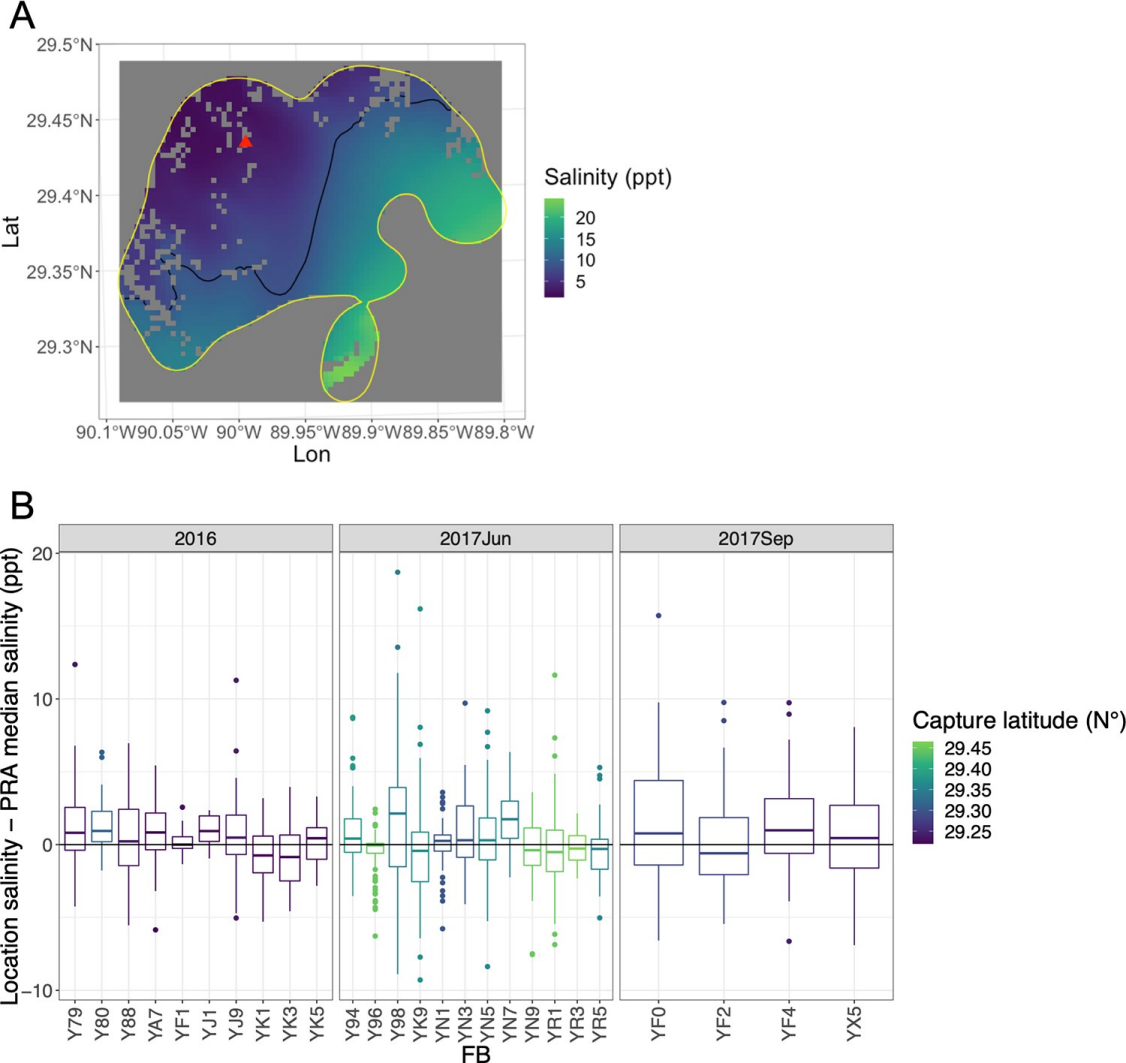
Dolphin salinity exposures compared to their potential ranging area (PRA) median salinity. We compared each dolphin’s salinity exposure (based on their telemetry locations) to the median salinity within their PRA. For example, on August 27, 2017, YK9 transmitted her location at the red triangle (A), which the Delft3D model predicted to be 3.2 parts per thousand (ppt). The median salinity within YK9’s potential ranging area that day (yellow contour) was predicted to be 10.6 ppt (black line). Thus, based on the modeled salinity, YK9 was in waters 7.4 ppt lower than the median salinity in her PRA. Across the duration of their tags, dolphins were typically in salinities within ± 2 ppt of their PRA median salinity, and were equally likely to be above or below the median salinity regardless of whether they were caught in the northern or southern parts of the stock area (B). Boxplots for all individuals from 2011 to 2017 are in the Supplemental Materials. Grey pixels within the PRA (A) represent land.

The estimated salinities at dolphin telemetry locations were typically similar to the median estimated salinity throughout their individual PRAs, and were equally likely to be above the median or below the median throughout the duration of the tags regardless of where the dolphin was captured within the basin (Figs 5 and S7). The difference between the telemetry locations and the median PRA salinity was on average within ±2 ppt for each dolphin, but occasionally dolphin locations were over ±10 ppt from their median PRA salinity. There was little difference among dolphin locations or movements across months, years, or locations for which we have telemetry data (S8 and S9 Figs): in all circumstances, there was no clear pattern of dolphin movement with respect to nearby salinity gradients. Barrier-island-associated dolphins stayed near the barrier islands regardless of salinity fluctuations (although salinities remained relatively high in those areas throughout the study period). Interior dolphins were typically found in waters near the PRA median salinity despite having higher salinity waters available to them, both within their PRA and in the broader BBES Stock area. For example, interior-associated dolphins transmitted from locations that were, on average, 7.8 ppt lower than the highest available salinity in each respective dolphin’s PRA (S10 Fig).

### Low salinity exposure and potential health effects

In total, seven dolphins were categorized into the low salinity cohort (based on the median salinity in their PRAs prior to their health assessment): six dolphins assessed in June 2017 and one in 2011 (S4 Table). The other 63 dolphins served as a reference group for comparison.

All of the dolphins with freshwater-like lesions had telemetry locations with a mean latitude near or north of Bassa Bassa Bay (S11 Fig). Freshwater-like skin lesions, specifically assessed only in 2017, were 3.3 (CI: 0.95–11.66) times more prevalent in the low salinity group than in the reference group (*p* = 0.054; Table 3). The small sample size for the exposure group (n = 6) created uncertainty in the estimates, as reflected in the relatively wide range of the confidence interval. The lesions ranged from mild, flat, circular areas of discoloration (S12A Fig) to more severe, depressed lesions of varying color and size (S12B Fig), but all of the freshwater-like lesions were multifocal to coalescing. The two dolphins in the reference group with freshwater-like skin lesions, YN7 and Y94 (S13 Fig), were also tagged and assessed in June 2017. YN7 had ten transmissions, with a potential ranging area of 33 km^2^. Y94 was mostly located near Bassa Bassa Bay and the surrounding wetlands. Fifteen additional dolphins were given skin evaluations (based on the data form in the Supplemental Materials) in September 2017 (but were not tagged). All of these dolphins were capture/released near Grand Isle in high salinity waters; none of these dolphins had freshwater-like lesions.

**Table 3:** The number of cases and prevalences of adverse health indicators in reference vs low salinity exposure cohorts.

	# of cases		Prevalence (95% CI)		*p*-value	
Health Outcome	Reference	Low salinity	Reference	Low salinity		Relative Risk (95% CI)
Freshwater-like skin lesions	2/8	5/6	0.25 (0.06–0.6)	0.71 (0.42–0.99)	0.054	3.33 (0.95–11.66)
Hepatobiliary panel	7/62	3/7	0.11 (0.05–0.22)	0.43 (0.16–0.75)	0.062	3.86 (1.28–11.64)
Electrolytes/minerals panel	1/62	1/7	0.02 (0–0.09)	0.14 (0.01–0.53)	0.200	9.00 (0.63–128.61)
Inflammation panel	12/ 62	2/7	0.19 (0.11–0.31)	0.29 (0.08–0.65)	0.631	1.38 (0.39–4.92)
Iron panel	10/62	1/7	0.16 (0.09–0.27)	0.14 (0.01–0.53)	0.983	0.90 (0.13–6.03)
Anemia panel	6/62	0/7	0.10 (0.04–0.2)	0.00 (0–0.4)	NA	NA
Hypoglycemia panel	4/62	0/7	0.06 (0.02–0.16)	0.00 (0–0.4)	NA	NA
Renal panel	0/62	0/7	0.00 (0–0.07)	0.00 (0–0.4)	NA	NA

To be considered consecutive, transmissions had to be within 48 hours—i.e., we assumed that dolphins that transmitted twice with 48 hours did not visit waters >5 ppt during that 48 hour gap, but that transmissions more than 48 hours apart allowed for the dolphin to move into waters >5 ppt (parts per thousand).

Dolphins in the low salinity exposure group were 3.86 (CI: 1.28–11.64) times more likely to have hepatobiliary abnormalities than the reference group (*p* = 0.062; Table 3). The three dolphins in the low salinity exposure cohort all had elevated liver enzymes levels, while the seven dolphins in the reference cohort had a mix of elevated or depressed liver enzyme levels (Supplemental Materials). Although there were one or two dolphins (depending on the specific panel) in the low salinity exposure group with abnormal levels of electrolytes/minerals, inflammation, or iron, a similar low prevalence for each panel was seen in the reference group, and low case numbers/sample size limited the statistical power to compare the groups. We did not observe anemia, hypoglycemia, or renal abnormalities in any of the low salinity exposure cohort.

We measured serum osmolality in June and September 2017, as well as plasma and urine osmolality in a subset of BBES dolphins. Serum osmolality was similar in the reference (mean = 345 mOsm kg^-1^; range 335–362 mOsm kg^-1^) and low salinity exposed groups (340 mOsm kg^-1^; range 330–358 mOsm kg^-1^). Plasma osmolality samples and analysis were only available in September 2017. Mean (± standard deviation) plasma osmolality for September was 344 ± 5 mOsm kg^-1^ (as compared to the mean serum osmolality of 349 ± 10 mOsm kg^-1^). The median difference in plasma and serum osmolality for September samples was 3.5 mOsm kg^-1^, and 75% of the differences were within ±4 mOsm kg^-1^, indicating that serum and plasma values are generally comparable.

Veterinarians collected urine samples for osmolality from three dolphins in June, and one dolphin in September 2017. YR3 and Y94 had very low values (597 and 662 mOsm kg^-1^, respectively) compared to a previously reported bottlenose dolphin urine osmolality value of 1,815 mOsm kg^-1^ [70]. We sampled YK9 in the central portion of the bay (northwest of Bassa Bassa, salinity = 5.2 ppt); YK9’s urine osmolality was 1,201 mOsm kg^-1^. In contrast, Y72, sampled in September near Grand Isle (salinity = 19.3 ppt), measured 1,857 mOsm kg^-1^ in its urine. Neither YK9 nor Y72 had evidence of freshwater-like skin lesions. Urine to serum osmolality ratios were 1.7, 1.9, 3.5, and 5.3 for YR3, Y94, YK9, and Y72, respectively.

## Discussion

### Potential ranging areas (PRAs)

Ranging patterns of dolphins in 2016 and 2017 were similar to the trends reported by Wells et al. [24]. All tagged dolphins stayed within the basin or within a few kilometers of the barrier islands, and throughout the basin, there were individual dolphins that maintained relatively small PRAs. Males tended to range farther than females near the barrier islands (although male and female interior dolphins had similar PRAs), and dolphins in the interior of the bay tended to range farther than island-associated dolphins. The barrier islands represent areas of especially high productivity, likely driven by the interface between the open ocean and the estuarine ecosystems, and Barataria dolphins congregate at high densities near the islands and the passes between them [1].

However, by targeting dolphins in the northern part of the study area, we also revealed patterns of movements with extended ranging along an east-west axis (e.g., YK9) and dolphins with high site-fidelity to wetlands in the northern extent of the BBES Stock area (e.g., Y96). These individuals’ PRAs and estimated salinity exposures reveal that earlier attempts to establish population-level habitat ranges (and sometimes interpreted as “salinity preferences” in the literature) using only dolphins caught south of Bassa Bassa Bay [57] or to correlate dolphin movements with modeled salinity estimates [58] should be re-evaluated using telemetry data and photo-identification/small vessel-based survey data from dolphins across the entire BBES Stock area. Across all catch-and-release field efforts, we found that the latitude at which a dolphin was captured correlated well with the mean latitude of their telemetry locations, emphasizing the importance of study designs that target dolphins throughout the basin for a holistic understanding of all BBES dolphins.

Although the telemetry data presented here provide additional insight into the movements of dolphins in a wider area over a few months, it is still not representative of the BBES Stock across all seasons, years, and geographic areas. It would also be informative to have additional movement and salinity data during the period from January to June, when salinities are decreasing in the basin. However, the PRAs defined by the telemetry data overlapped with more than 93% of the small vessel survey sightings for the dolphins in this dataset, with the majority of the tagged dolphins (63 out of 70) seen within their PRAs over multiple years from 2010 through 2019. Six of the dolphins (including four island-associated and two interior dolphins) were observed within their respective PRAs in all ten years of surveys. The similarity between the PRAs and the decade-long sighting histories supports the previous observations that bay, sound, and estuary dolphins in the nGOMx have high site-fidelity to particular areas within their stock areas across seasons and years.

### Dolphin movements relative to salinity gradients

Despite the heterogeneity of movement patterns exhibited by individual dolphins, we did not see any evidence that any of the dolphins moved in association with salinity changes. Based on the low salinity estimates of dolphins’ locations in the northern part of the study area in June/July 2017, it is clear that these dolphins did not avoid low salinity waters even though they theoretically could have moved into higher salinity waters closer to the barrier islands (Figs 2 and 3; S6 and S7 Figs). Rather, throughout the tagging period—a period of time with low salinity from the spring runoff and then increasing salinities over the late summer/fall months—there was no clear pattern of dolphin movement with respect to salinity gradients within their PRAs (Fig 4). Throughout the 2011–2017 dataset, few dolphins moved across the entire north-to-south extent of the BBES Stock area, and when they did, there was no obvious consistent trend up or down salinity gradients in geographic space or time. There was no temporal trend to individual dolphin locations with estimated salinities above and below the median salinity within their PRAs (S8 and S9 Figs); in total, the range and the average of estimated salinities at each individual dolphin’s telemetry locations were very similar to the respective median salinity ranges/averages for individual dolphins’ PRAs (Figs 5 and S7). In other words, dolphins were equally likely to be found in above-average salinity and below-average salinity within their PRAs, regardless of the overall seasonal, basin-wide salinity trends. Even island-associated dolphins tagged in 2013 remained near the eastern barrier islands in salinities <10 ppt rather than range into the open waters of the nGOMx to higher salinity waters (Fig 3), demonstrating high site fidelity irrespective of salinity.

As with any model, the Delft3D salinity estimates have unavoidable uncertainty and bias. Thus, absolute values of the predictions must be interpreted with appropriate caution. Ideally, this study would be repeated using satellite-telemetry tags with integrated salinity/conductivity sensors, so that every telemetry location is paired with a real-time salinity measurement. While conductivity-temperature-depth satellite-telemetry tags have been developed and successfully tested on large cetaceans in northern latitudes [71], such technology has not been integrated into the single-pin dorsal fin tags used for bottlenose dolphins. More importantly, the technology has not been successfully tested in warmer regions, where biofouling is a significant issue for the conductivity sensors. Therefore, such tags were not an option at the time of our study.

When comparing the field-based salinity measurements at the capture locations to the Delft3D estimates for those same dates/locations, the model underestimates higher salinities near the barrier islands and overestimates lower salinities in the northern part of the study area (S5 Fig). If the trend holds true across the larger model dataset (our findings are consistent with other assessments of the Delft3D model [72,73]), actual salinities in the northern portion of the study area are likely lower than the model predictions, and therefore our results represent conservative estimates of low salinity exposure to Barataria dolphins. However, a significant strength of analyzing the Delft3D daily salinity fields is the ability to assess relationships over geographic space and time and make relative comparisons among individuals. Corrections for bias over space are unlikely to change our observations that dolphins were equally likely to move to areas above or below the median salinity of their PRA.

### Low salinity exposures and health outcomes

In our analyses of the modeled salinity data and the telemetry data, we observed that most of the dolphins in June 2017 experienced at least two days in waters less than 5 ppt, with Y96 transmitting from locations under 5 ppt on 72 days. At least five dolphins (four in 2017 and one in 2011) spent at least five consecutive days under 5 ppt. Although little information is available about the relationship among durations of low salinity exposure, specific thresholds for low salinity exposure, and health outcomes for bottlenose dolphins and other marine mammals, many studies have associated prolonged exposures of cetaceans to waters at least < 20 ppt with skin lesions [8–12], abnormal blood chemistry indicators [16,18], and overall poor health/increased mortality [16,17,19–21,74]. Consistent with these studies, we documented freshwater-like skin lesions, hepatobiliary abnormalities, and reduced osmolality measurements in dolphins likely exposed to at least two days of low salinity (<5 ppt).

Compared to previously reported skin lesions for bottlenose dolphins after rapid declines in salinity to near-freshwater [21] or prolonged exposure in freshwater environments [19], our veterinary team considered the observed freshwater-like lesions to be mild. This could be related to the duration of exposure, the severity of hyposalinity exposure, the stage of the skin disease, or other factors we did not measure. The freshwater-like lesions were limited to dolphins in the northern part of the study area; none of the island-associated dolphins had freshwater-like lesions. Although the small sample size of northern dolphins limited our analyses, the low salinity exposure group had an increased risk for freshwater-like skin lesions and hepatobiliary abnormalities.

The three abnormal hepatobiliary cases were all characterized by elevated aspartate transaminase (AST) along with at least one other elevated liver enzyme. Our observations of elevated hepatobiliary enzymes are consistent with recent findings from Ewing et al. [16], who reported elevated AST and alanine transaminase (ALT) in dolphins exposed to salinity of less than 10 ppt for more than 5 days. All three of the hepatobiliary cases in the low salinity exposure cohort also had freshwater-like skin lesions. While the mild skin lesions and elevated hepatobiliary enzymes are consistent with early stages of disease, we cannot determine how the skin lesions and blood work abnormalities changed over the following months as the low-salinity exposure continued.

Healthy dolphins typically respond to ingestion of hypo- or hyper-saline fluid by altering solute clearance and urine osmolality [75]. We were only able to collect and analyze plasma osmolality samples from dolphins in September 2017, which likely had high salinity exposure prior to the health assessments. Their plasma osmolality is consistent with the ranges seen in healthy dolphins [76,77] and dolphins in an experimental study that fed dolphins freshwater or seawater [78]. While we were only able to collect urine samples from three dolphins in June, we found that interior dolphins (YR3 and Y94) sampled during this low-salinity period (1.8 and 2.3 ppt at their capture site, respectively) had urine osmolality values and ratio of serum:urine osmolality values that were lower than any previously reported for bottlenose dolphins, including dolphins fed deionized water in an experimental study [78]. Both YR3 and Y94 were sampled north of Bassa Bassa Bay, and both exhibited freshwater-like skin lesions. Interestingly, serum osmolality values for these two dolphins were non-remarkable, suggesting that they were osmoregulating/compensating following the freshwater exposure either by altering urine osmolality (which were low) or clearance (which was not measured). Further studies, ideally collecting samples after a prolonged exposure to low salinity, would help to better understand how long such compensation could continue before overwhelming the dolphin’s homeostatic mechanisms. These findings support the hypothesis that duration of exposure to a low salinity environment is an important consideration in addition to the degree of low salinity.

While our telemetry results demonstrate that at least one dolphin, Y96, was able to survive multiple months in salinities less than 5 ppt (Fig 4), it does not indicate that they will maintain good health or that their probability of survival was not decreased. At the time of tagging, the median salinity in Y96’s PRA was less than 5 ppt for at least two of the previous seven days, and Y96 exhibited freshwater-like skin lesions, low body weight, and abnormal electrolyte/mineral and hepatobiliary health panel results. Following the June 2017 field effort, the Barataria Basin underwent a period of prolonged low salinity conditions; however, we have no information about the health status of dolphins during/after this time. Therefore, we are unable to determine if the dolphins’ health was stable or declining during the period over which we received data from satellite-telemetry tags. We simply know that the dolphins were still alive, based on the tracking data. Four of the seven dolphins in the low salinity cohort were seen in 2019 small vessel surveys within Barataria Bay, but Y96 was not one of them. Future studies could attempt to evaluate skin condition and/or health of the tagged dolphins near the end of estimated tag durations.

If, as our results suggest, dolphins’ high site-fidelity to local areas within the Barataria Basin puts them at risk of prolonged seasonal exposures to low salinity, then it is likely that the longer durations, more sudden changes, and even lower salinities expected as a result of the proposed Mid-Barataria Sediment Diversion will increase the likelihood of low salinity-related adverse health effects, which may lead to an increased number of dolphin mortalities. In addition to the potential diversion, dolphins living in bays, sounds, and estuaries along the Gulf coast are increasingly threatened by freshwater-related effects from climate change, including severe storms, coastal erosion, increased rainfall, and flooding. Given the two recent UMEs affecting dolphins in the nGOMx [23,54], monitoring and assessing how changes in salinity impact dolphin behavior and health is important to inform long-term restoration efforts for dolphin populations and their habitat.

A recent study of dolphins in Pensacola Bay, Florida demonstrated that the distribution of dolphin groups did not substantially change in response to salinity fluctuations caused by a record-breaking freshwater flood event in 2014 [79]. However, the Pensacola Bay area is a smaller estuary/bay system (~462 km^2^) that is characterized by deeper mid-system waters and water column stratification even in shallow waters, and where there’s evidence that individual dolphins tend to range throughout the system. Dolphins had access to stratified areas with higher bottom salinities that persisted throughout the flood event. Other than near the passes between the barrier islands, BBES dolphins have very little access to waters deeper than 2–3 m, likely limiting the potential for similar high salinity refuges via stratification.

By studying top-level predators in habitats that are rapidly changing, we can better understand the broad-scale impacts of cumulative stressors on the overarching ecosystem. Cetaceans with high site-fidelity (such as BBES dolphins) tend to be the least resilient to changes in habitat conditions and are more likely to be listed as threatened/endangered [80]. Our results have applicability to Southeastern U.S. bottlenose dolphin stocks, as well as marine mammal populations/species worldwide that have long-term site fidelity and limited ranging patterns to a given habitat. For example, dolphins in Australia (*Tursiops aduncus* and *Tursiops australis*) exhibited similar skin lesions after sudden and weeks/months-long exposure to hypo-saline conditions due to intense rainfall [13]. Indo-Pacific humpback dolphins (*Sousa chinensis*) in China and Taiwan [81–83] and the Chilean and Guinean dolphins (*Cephalorhynchus eutropia* and *Sotalia guianensis*, respectively) in South America [84,85] are also species/populations that are impacted by many of the same environmental stressors as Barataria dolphins. In addition, by monitoring salinity conditions in coastal and estuarine small cetacean habitats, it may be possible to predict where and when increased numbers of strandings will occur, allowing wildlife managers to improve interventions, responses, and preparations for rehabilitation [86]. As humans continue to re-engineer the hydrological networks through “water transfer mega projects”, such as the proposed Mid-Barataria Sediment Diversion, studies that integrate various parameters (e.g., animal movement, animal health, and environmental data) will be essential to evaluate the associated impacts [87]. A better understanding of how environmental, anthropogenic, and climatic changes combine to affect marine mammal mortality will be paramount for conservation and management efforts in the future.

## Conclusions

Across our analyses, we found no evidence that satellite-telemetry tagged dolphins moved in association with changes in salinity. Individual tagged dolphins showed a variety of movement patterns during the transmission window of the tags, but none were associated with trends in salinity over time or geographic space. We also saw that dolphins that are predicted to have been exposed to low salinity (<5 ppt) for at least two days in the week prior to the health assessments were more likely to have freshwater-like skin lesions and hepatobiliary abnormalities. Our results show that dolphins in the northern parts of the BBES Stock area did not move to avoid low salinity conditions for the duration of the satellite-telemetry- tags, despite experiencing adverse health effects secondary to low salinity, consistent with the concept of “ecological cul-de-sacs” [88]. Thus, it is likely that other factors drive Barataria dolphin movements, including prey availability, occurrence of predators, mating opportunities, calving events, age, sex, and/or social organization.

Although our comparisons between the seasonal telemetry data and the decade-long sighting history suggests that the PRAs represent dolphin activity centers across years, we cannot necessarily assume that these patterns would hold if sampling were conducted outside the conditions present when we conducted our fieldwork. For example, it is unknown whether this pattern continues into the eastern part of the basin. It would also be preferable to conduct similar analyses using measured salinity values at each dolphin location instead of the model simulations. This could be accomplished using satellite-telemetry tags with conductivity sensors. Conducting paired health assessments at the beginning and end of the satellite-telemetry tag lifetimes, after prolonged exposure to low salinity, would be the most robust (but also logistically challenging) means of identifying dose-time-response relationships between defined magnitudes/durations of low-salinity exposures and related adverse health effects. However, our analyses do provide some insight into potential biomarkers to monitor (e.g., hepatobiliary enzymes, urine osmolality, and urine:serum osmolality ratio) before, during, and after diversion operations. Our results can also provide reference data for interannual comparisons for future studies.

## Supporting information

S1 File Supplementary figures and tables. This document contains S1–S13 Figs and S1–S4 Tables.
(PDF)

S2 File Individual bottlenose dolphin observations and PRAs. These plots combine information about each dolphin’s 1) history of photographic identification survey observations (points colored by year of observation), 2) locations received during deployment of satellite telemetry tags (black points), and 3) the potential ranging area (PRA) determined by the satellite transmissions (black contour). The dolphin ID is provided at the top of each plot along with the general pattern of usage (either Island or Interior).
(PDF)

S3 File Individual dolphin health observations. This spreadsheet contains health assessment data from live dolphins near Barataria Bay, LA using methods previously described. Relevant data for each live wild dolphin sampled include sampling date, sex, morphometrics, and diagnostic test results including hematology and serum chemistry values, osmolality measurements, and results of tests assessing multiple indicators for specific physiological systems.
(XLSX)

S4 File Observations of common bottlenose dolphins in the Barataria Basin from photographic identification surveys from 2010 to 2019. (XLSX)

S5 File Skin assessment form. The data recording sheet for assessing skin health during health assessments in the Barataria Basin.
(PDF)

S6 File Salinity estimates. This spreadsheet contains the daily salinity estimates at the transmitted locations for each dolphin, their PRA, and the Barataria Basin, based on the Delft3D model output.
(XLSX)
