# Supplementary material for: High site-fidelity in common bottlenose dolphins despite low salinity exposure and associated indicators of compromised health
Source: PLoS One. 2021 Sep 30;16(9):e0258031. doi: 10.1371/journal.pone.0258031 (PMC8483354; doi:10.1371/journal.pone.0258031)
Supplement: S1 File — This document contains S1–S13 Figs and S1–S4 Tables. (PDF) [file pone.0258031.s001.pdf]

High site-fidelity in common bottlenose dolphins despite low salinity exposure and associated indicators of compromised health

R Takeshita*[1], BC Balmer[1], F Messina[2], ES Zolman[1], L Thomas[3], RS Wells[4], CR Smith[1], TK Rowles[5], and LH Schwacke[1]

Supplemental Figures and Tables

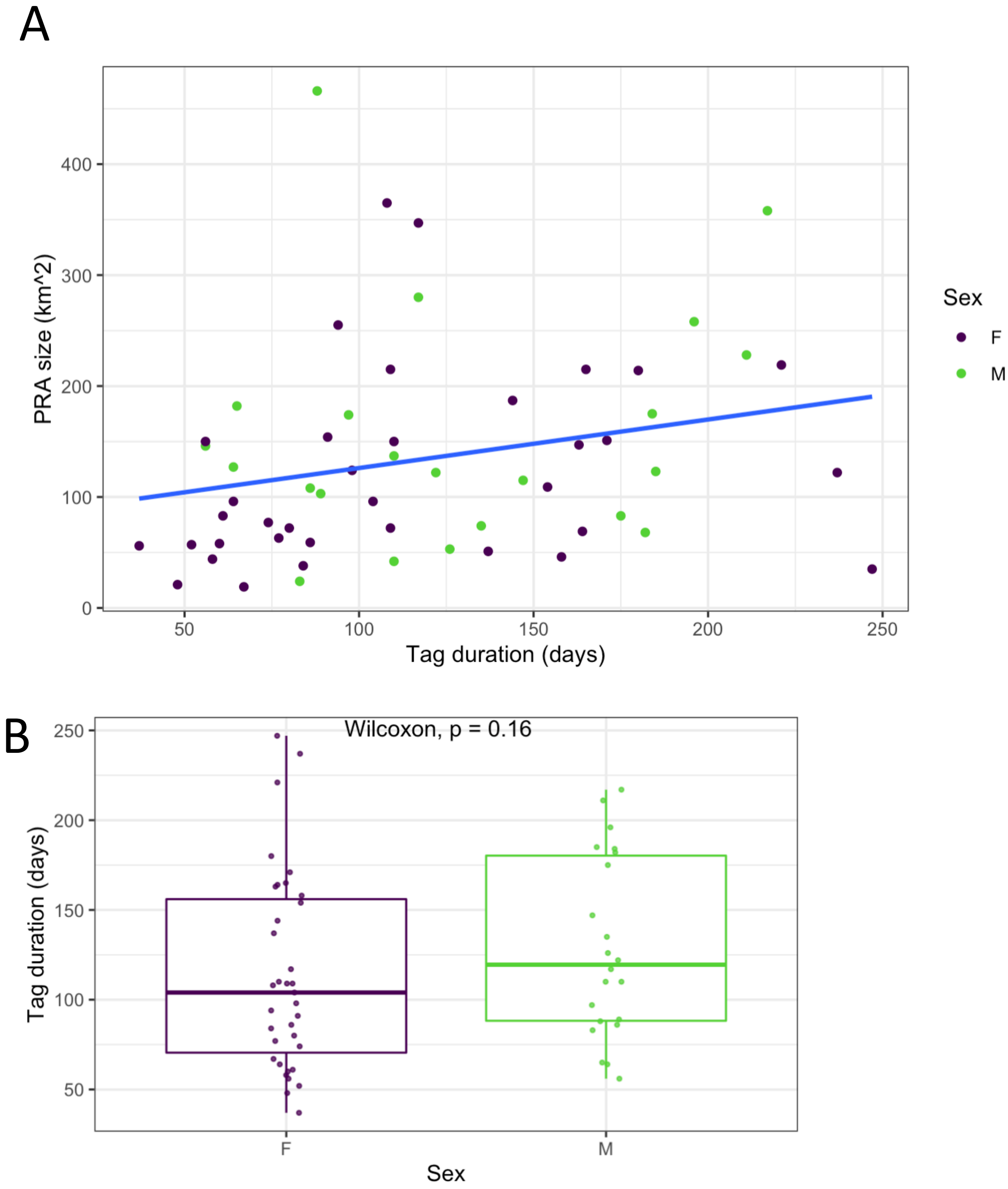

Figure S1: Comparing the length of time each tag transmitted with the respective PRA size for each individual dolphin and by sex. (A) Tag duration is not a strong predictor of PRA size (R^2 = 0.06; $F(1,55) = 3.5$; $p < 0.068$). (B) There was little difference between how long tags transmitted for males (purple) and females (green) (Wilcoxon test $p < 0.16$).

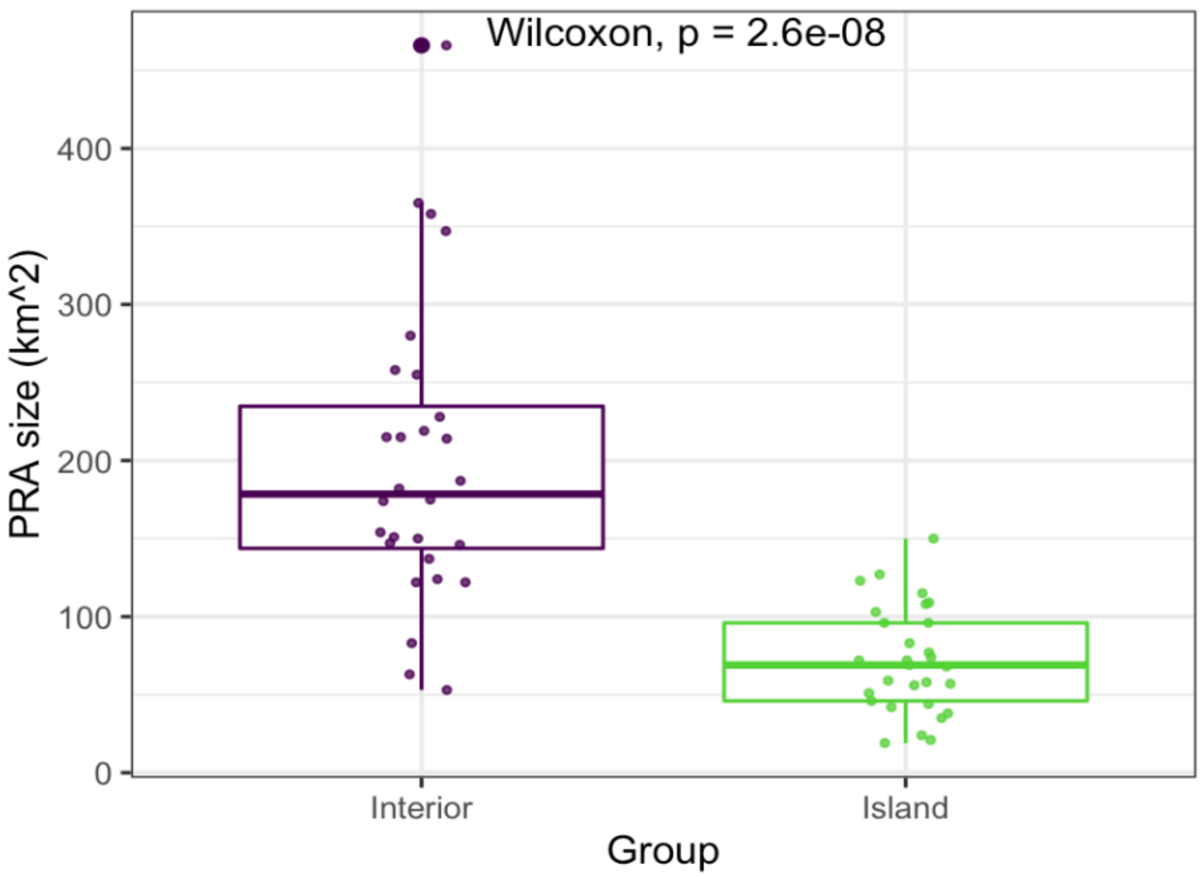

Figure S2: Comparing the PRA sizes of dolphins associated with the barrier islands with dolphins that spent time in the interior of the basin. Barrier island-associated dolphins were more likely to have smaller PRA sizes than interior dolphins (Wilcoxon test $p < 0.000$).

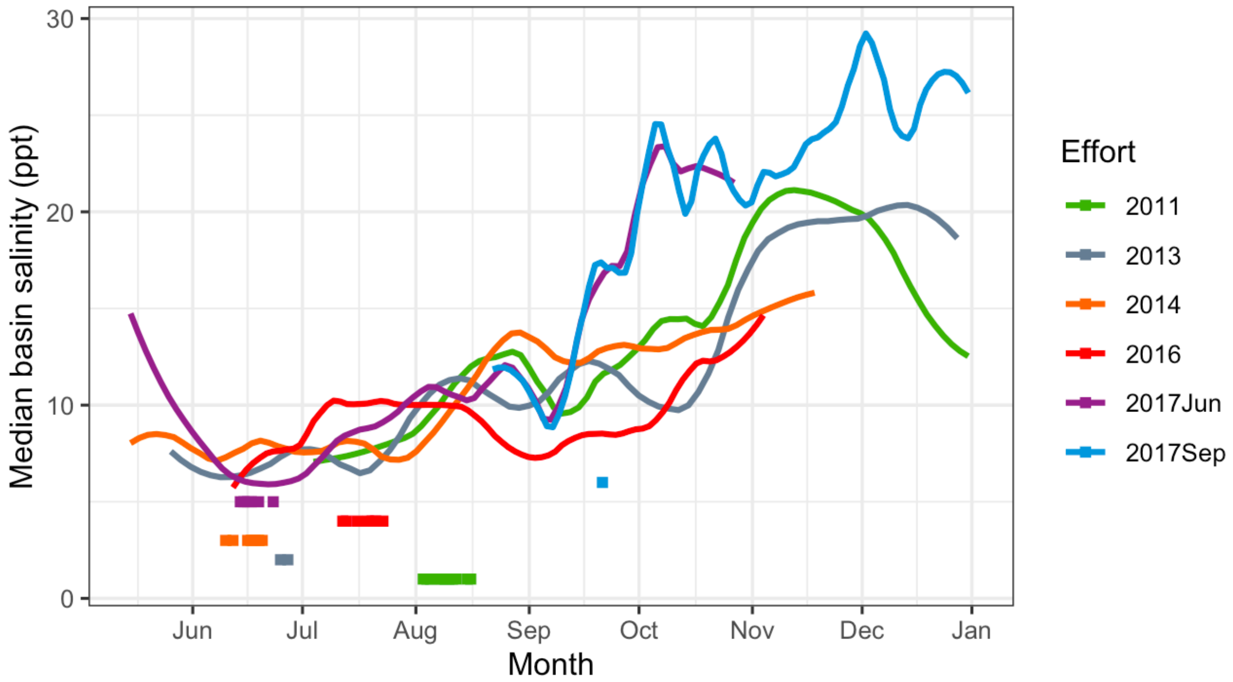

Figure S3: Median basin-wide salinity estimates over time. Loess curves (span = 20%) are provided for the month prior to tagging and then the duration of the longest-lived tags for each field effort. Colored bars at the bottom of the plot denote the days when each tagging/health assessment effort took place.

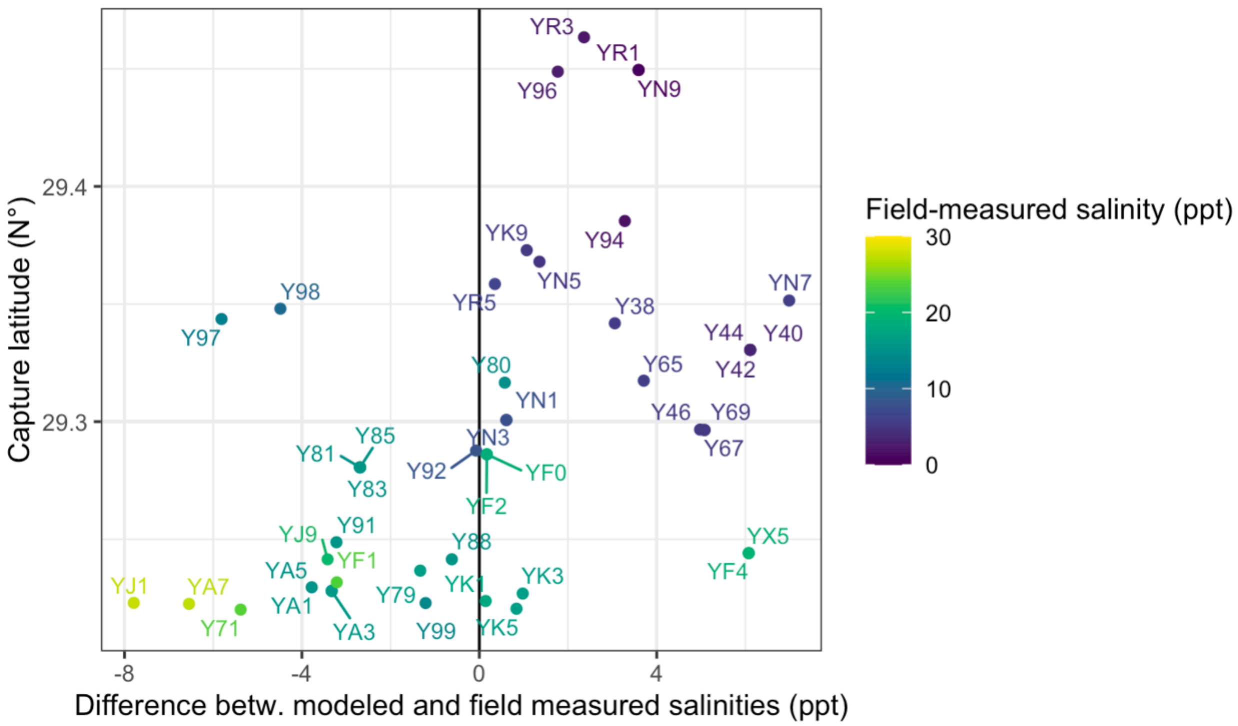

Figure S4: Comparing modeled salinity estimates with salinity measurements in the field. The Delft3D model tended to underestimate salinities for dolphins captured near the barrier islands in higher saline waters and overestimate salinities for dolphins captured in the northern part of the study area in lower saline waters, when compared to the salinity measurements taken during the field activities.

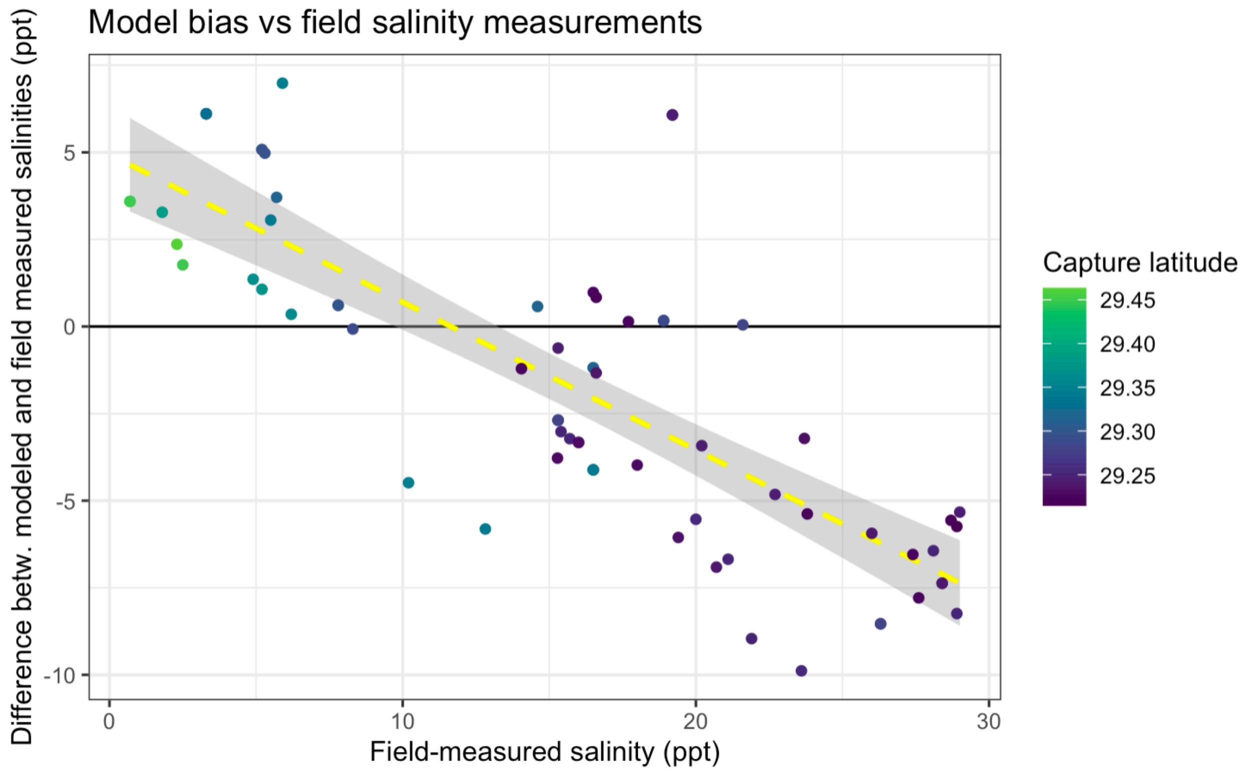

Figure S5: The Delft3D model overestimated salinity in higher latitudes (where salinities were below 10 ppt during capture-release activities based on field measurements), but the model underestimated salinity in lower latitudes (where salinities were above 10 ppt based on field measurements). There is a strong linear relationship between the model bias and the field-measured salinity (β = -0.42; R^2 = 0.63; F(1,68) = 117.4; p < 0.000).

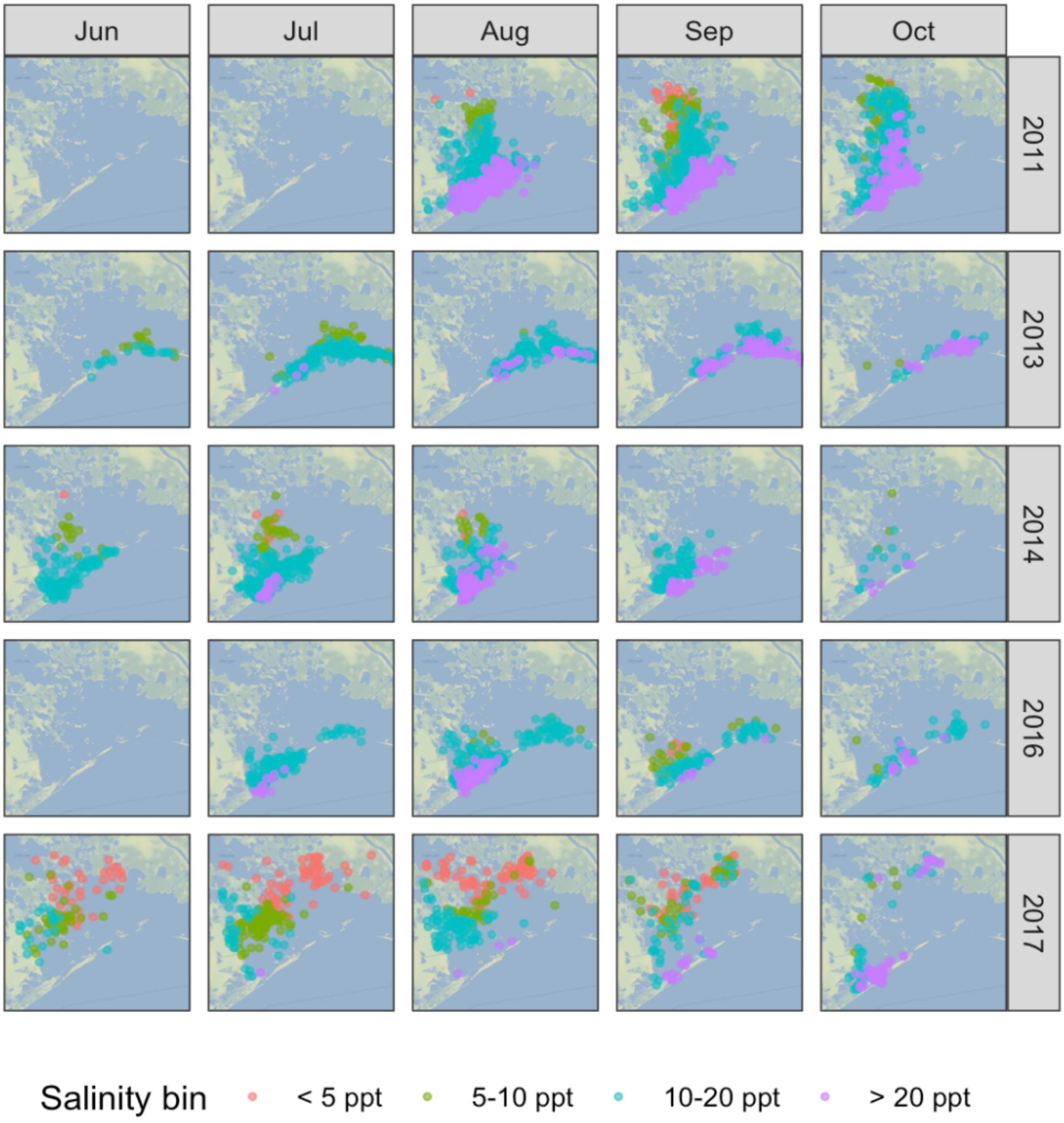

Figure S6: The estimated salinity at each telemetry location by year and month. We binned the Delft3D modeled salinity for each telemetry transmission and mapped all individuals. Dolphins predicted to be in low salinities (e.g., < 5 ppt) tend to be in the northern part of the basin during the summer months, but only a small number of tagged dolphins moved to the north prior to the June 2017 effort. Capture/tagging/release locations were selected for each effort based on different study questions and priorities.

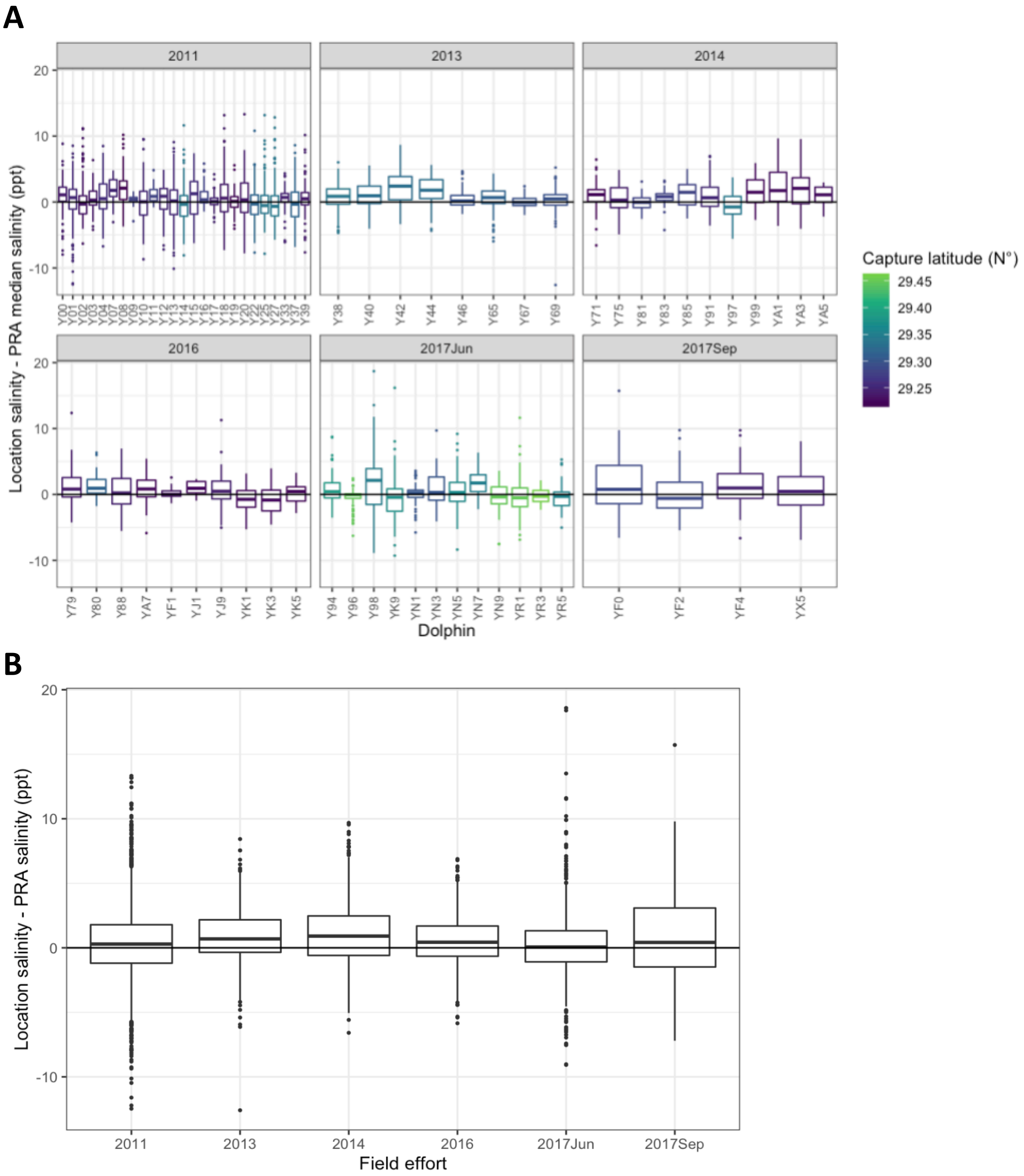

Figure S7: Dolphin salinity exposures compared to their potential ranging area (PRA) median salinity. We compared each dolphin's salinity exposure (based on their telemetry locations) to the median salinity within their potential ranging area (A). Across the duration of their tags, dolphins were typically in salinities within ± 2 ppt of their potential ranging area median salinity, and were equally likely to be above or below the median salinity, regardless of the latitude at which they were caught (A) or effort (B).

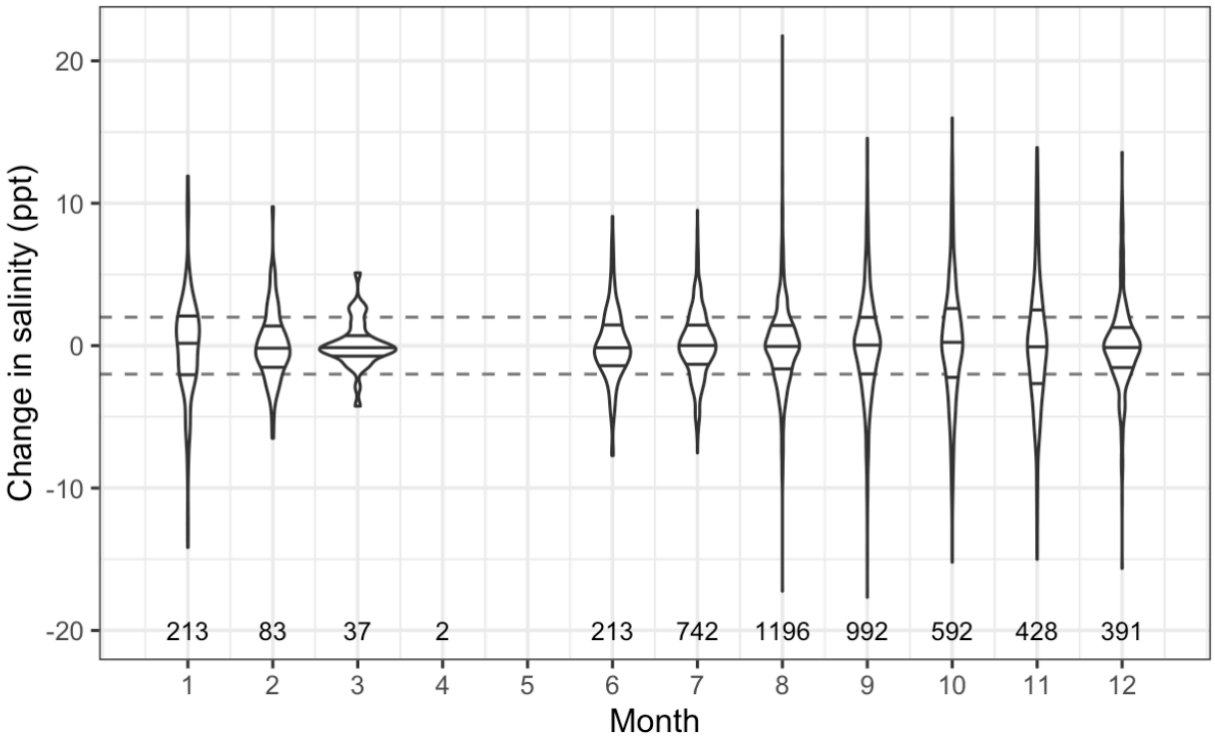

Figure S8: Dolphin movements in relation to salinity gradients. Between consecutive telemetry locations, dolphins typically moved into waters with similar salinities (dashed lines mark ± 2 ppt), but when they did move into waters with larger differences in salinity, they were about equally likely to move into higher or lower salinity, regardless of whether the median basin salinity (red) was rising (June–November) or falling (December–June). The total number of consecutive transmission pairs for each month is given at the bottom of the plot, but each violin is scaled proportionally so that they all have the same area. There were only two transmissions in April and no transmissions in May. The horizontal lines within each violin represent the interquartile range and the median. If dolphins preferred to move into higher salinity waters, we would expect to see the most density within each violin above the 0 ppt line.

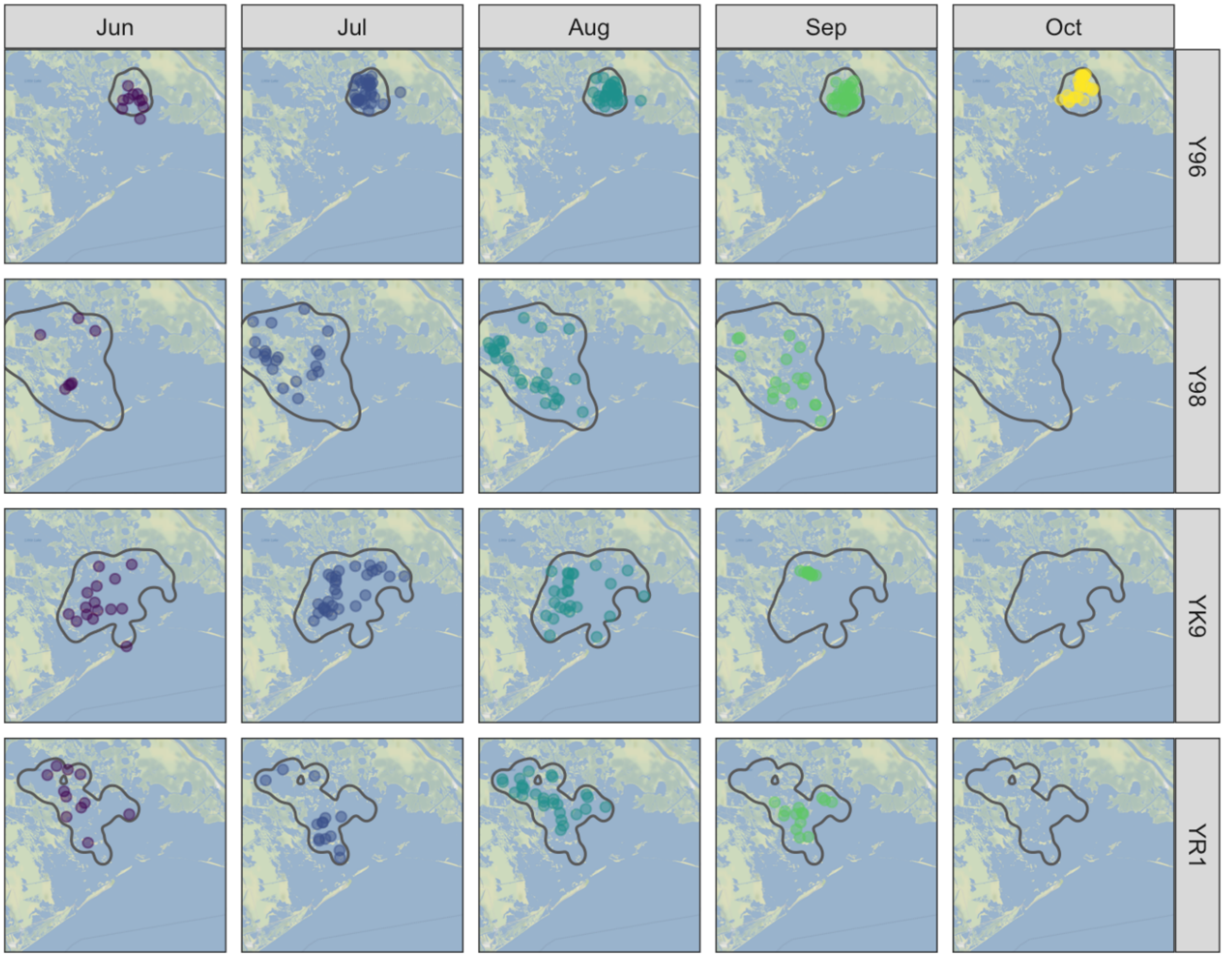

Figure S9: Dolphin locations within their potential ranging area by month. Individual dolphins used the extent of their potential ranging area equally across months. There was no obvious trend where dolphins preferred lower latitudes within their potential ranging area during the spring/summer runoff.

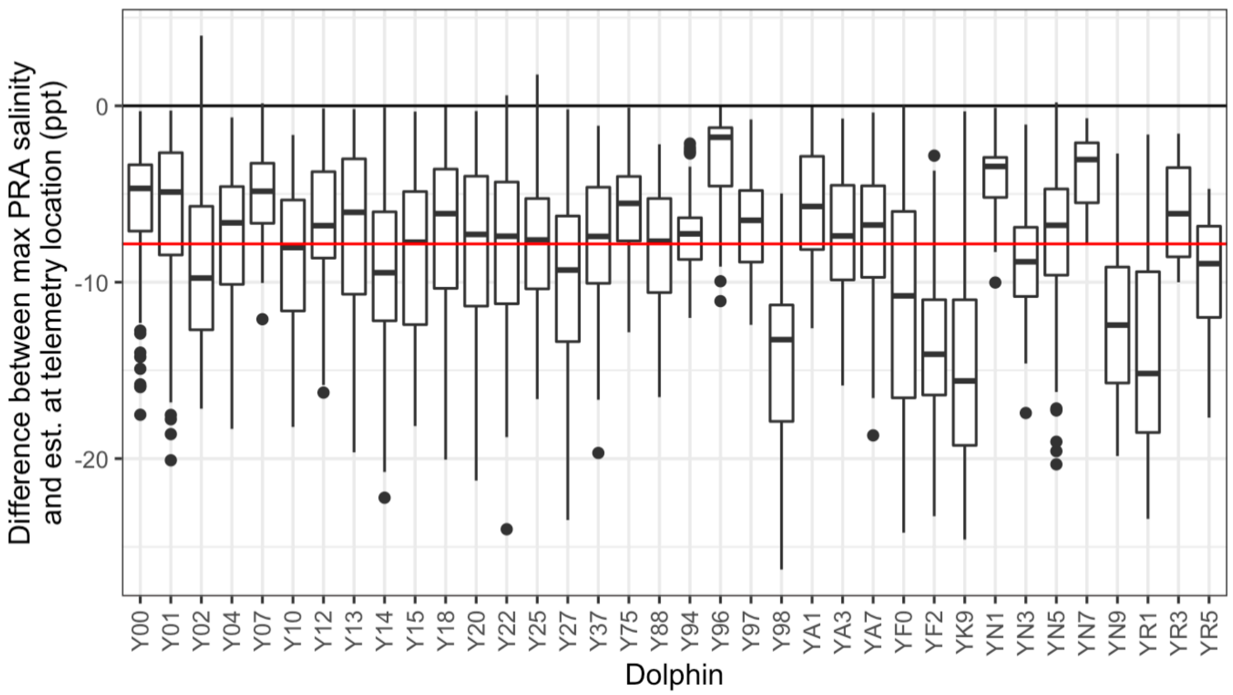

Figure S10: Boxplots of the difference between the modeled salinity at each dolphin telemetry location and the maximum salinity within each dolphin's PRA for that day. On average, dolphins were in waters 7.8 ppt (red line) below the maximum "available" salinity within their respective PRAs.

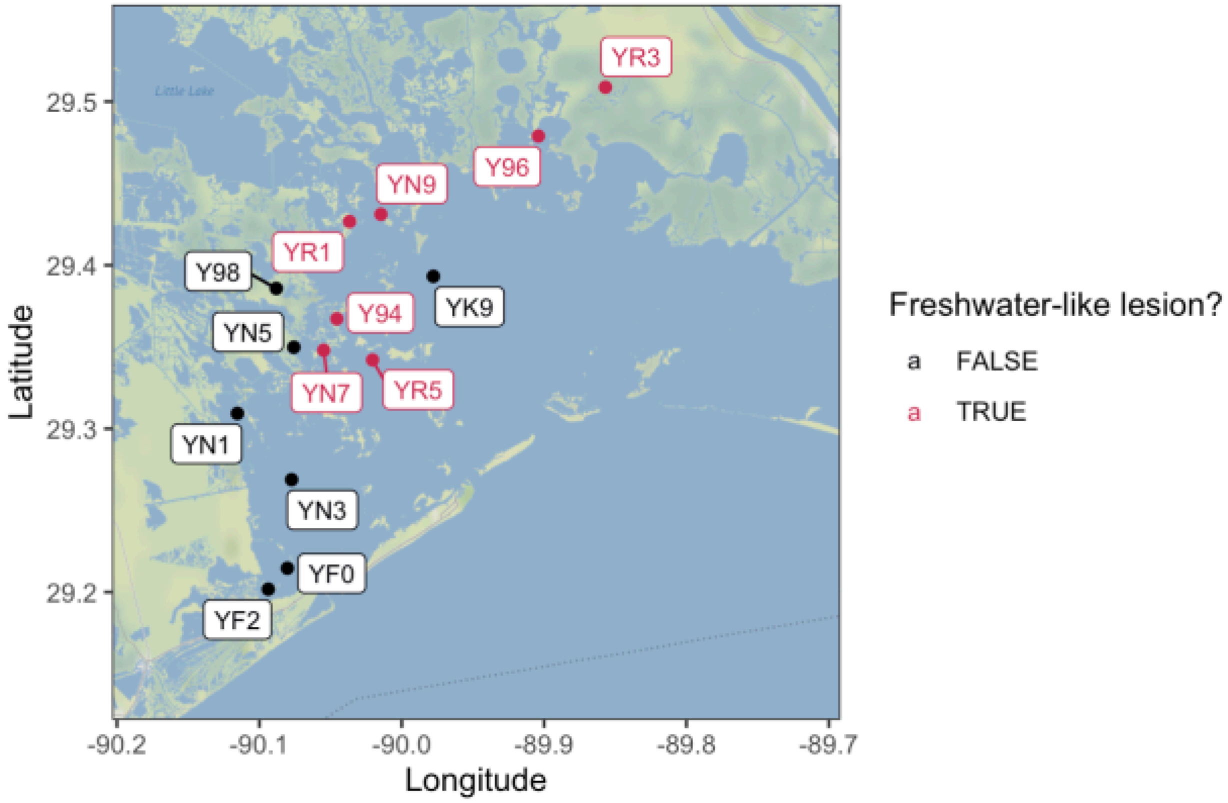

Figure S11: Mean telemetry locations of dolphins given skin assessments using the form in the Supplementary Information. Only individuals with movements mostly north of Bassa Bassa Bay (~ 29.34 N°) had freshwater-like lesions (red). Individuals that were mostly associated with the barrier islands or Caminada Bay (e.g., YF0 and YF2) did not have freshwater-like lesions (black).

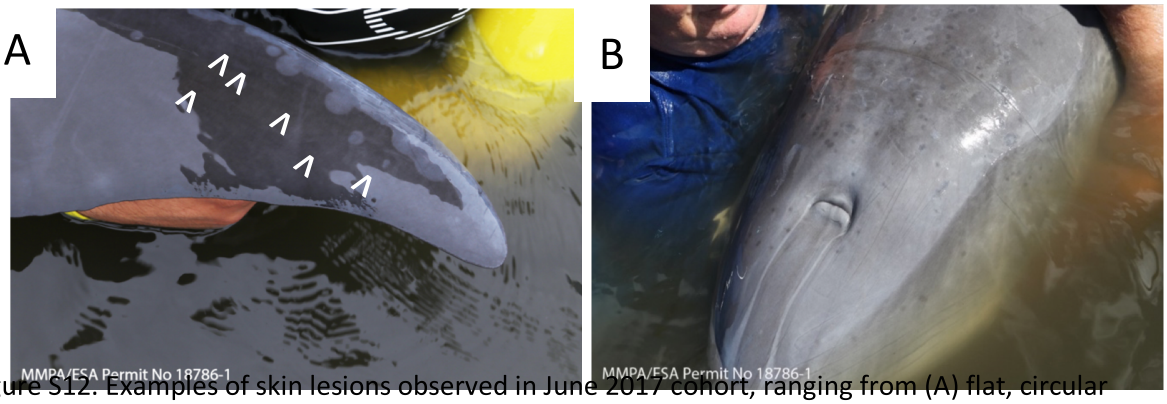

Figure S12. Examples of skin lesions observed in June 2017 cohort, ranging from (A) flat, circular areas of discoloration (white arrowheads) seen in YR1; to (B) more severe, depressed lesions of varying color and size observed in YR3.

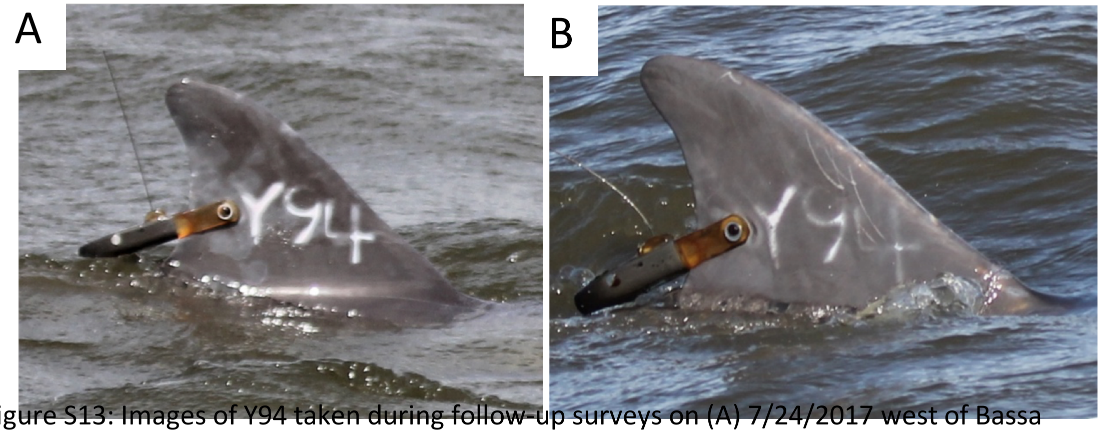

Figure S13: Images of Y94 taken during follow-up surveys on (A) 7/24/2017 west of Bassa Bassa Bay (salinity = 5.55 ppt), and (B) 11/20/2017 in Hackberry Bay (salinity = 10.1 ppt). Skin lesions observed in July appear to be resolving by November following a period of increasing salinity. Photo credit: LDWF.

Table S1: Summary of dolphins with satellite-linked telemetry tag data from 2011 to 2017. FB: Dolphin identification; Lat: Latitude; Long: Longitude; Tag mft: Tag manufacturer; WC: Wildlife Computers; ST: Sirtrack Ltd.

FB	Date	Lat.	Long.	Sex	Length (cm)	Salinity at capture	Tag mft.	Tag type	# of included transmissions	Duration (days)
August 2011										
Y00	8/4/11	29.238	-90.013	M	232	20.7	WC	SPOT-100	126	110
Y01	8/3/11	29.250	-89.985	F	243	15.4	WC	SPOT-100	236	237
Y02	8/4/11	29.231	-90.091	M	263	18.0	WC	SPOT-100	98	97
Y03	8/3/11	29.234	-90.015	F	221	19.4	WC	SPOT-100	65	74
Y04	8/7/11	29.256	-89.995	M	255	20.0	WC	SPOT-100	94	65
Y07	8/5/11	29.248	-89.993	F	199	21.9	WC	SPOT-100	170	180
Y08	8/9/11	29.215	-90.030	M	274	28.9	WC	SPOT-100	73	64
Y09	8/5/11	29.280	-89.929	F	241	26.3	WC	SPOT-100	64	48
Y10	8/9/11	29.249	-90.023	M	258	28.9	WC	SPOT-100	167	217
Y11	8/5/11	29.280	-89.929	F	253	26.3	WC	SPOT-100	62	52
Y12	8/10/11	29.249	-90.025	M	261	28.1	WC	SPOT-100	52	175
Y13	8/7/11	29.251	-90.005	F	230	21.1	WC	SPOT-100	202	171
Y14	8/11/11	29.343	-89.990	M	216	16.5	WC	SPOT-100	223	211
Y15	8/7/11	29.255	-90.014	F	198	23.6	WC	SPOT-100	43	147
Y16	8/12/11	29.280	-89.942	M	273	21.6	WC	SPOT-100	62	110
Y17	8/8/11	29.244	-90.010	F	209	22.7	WC	SPOT-100	202	247
Y18	8/15/11	29.238	-90.012	M	271	28.4	WC	SPOT-100	245	196
Y19	8/9/11	29.218	-90.028	F	227	28.7	WC	SPOT-100	44	71
Y20	8/15/11	29.238	-90.012	M	267	28.4	WC	SPOT-100	151	117
Y22	8/16/11	29.318	-89.980	M	234	16.5	WC	SPOT-100	182	184
Y25	8/11/11	29.343	-89.990	F	239	16.5	WC	SPOT-100	182	163
Y27	8/11/11	29.343	-89.990	F	239	16.5	WC	SPOT-100	225	221
Y33	8/12/11	29.245	-89.966	F	228	29.0	WC	SPOT-100	144	158
Y37	8/16/11	29.318	-89.980	F	225	16.5	WC	SPOT-100	180	165
Y39	8/16/11	29.238	-90.010	F	242	26.0	WC	SPOT-100	150	137
June 2013										
Y38	6/25/13	29.342	-89.832	M	226	5.5	WC	SPOT-299B	166	147
Y40	6/25/13	29.331	-89.827	M	211	3.3	WC	SPOT-299B	149	135
Y42	6/25/13	29.331	-89.827	M	226	3.3	WC	SPOT-299B	142	185
Y44	6/25/13	29.331	-89.827	M	221	3.3	WC	SPOT-299B	105	86
Y46	6/27/13	29.297	-89.910	M	206	5.3	WC	SPOT-299B	84	83
Y65	6/27/13	29.317	-89.872	F	236	5.7	WC	SPOT-299B	90	80
Y67	6/27/13	29.296	-89.912	F	223	5.2	WC	SPOT-299B	82	67
Y69	6/27/13	29.296	-89.912	F	213	5.2	WC	SPOT-299B	128	109

June 2014										
Y71	6/10/14	29.220	-90.026	F	239	23.8	WC	SPOT-299B	78	84
Y75	6/12/14	29.240	-90.016	F	241	ND	WC	SPOT-299B	123	91
Y81	6/16/14	29.281	-89.942	F	241	15.3	WC	SPOT-299B	54	86
Y83	6/16/14	29.281	-89.942	F	243	15.3	WC	SPOT-299B	43	97
Y85	6/16/14	29.281	-89.942	F	230	15.3	WC	SPOT-299B	51	64
Y91	6/17/14	29.249	-89.991	F	243	15.7	WC	SPOT-299B	140	154
Y97	6/18/14	29.344	-89.991	F	229	12.8	WC	SPOT-299B	75	144
Y99	6/19/14	29.223	-90.023	F	259	14.1	WC	SPOT-299B	90	56
YA1	6/19/14	29.228	-90.020	F	251	16.0	WC	SPOT-299B	158	109
YA3	6/19/14	29.228	-90.020	F	241	16.0	WC	SPOT-299B	143	117
YA5	6/20/14	29.230	-90.021	F	242	15.3	WC	SPOT-299B	46	29
July 2016										
Y79	7/22/16	29.237	-90.017	F	224	16.6	WC	SPOT-299A	167	104
Y80	7/20/16	29.317	-89.874	M	249	14.6	WC	SPOT-299A	134	89
Y88	7/22/16	29.241	-90.008	M	245	15.3	WC	SPOT-299A	47	49
YA7	7/11/16	29.223	-90.023	F	213	27.4	WC	SPOT-299A	143	98
YF1	7/11/16	29.232	-90.019	F	237	23.7	WC	SPOT-299A	70	37
YJ1	7/12/16	29.223	-90.024	F	243	27.6	WC	SPOT-299A	16	14
YJ9	7/15/16	29.241	-90.007	F	226	20.2	WC	SPOT-299A	107	61
YK1	7/18/16	29.224	-90.024	F	235	17.7	WC	SPOT-299A	47	48
YK3	7/18/16	29.227	-90.021	F	264	16.5	WC	SPOT-299A	78	60
YK5	7/19/16	29.220	-90.026	F	235	16.6	WC	SPOT-299A	66	58
June 2017										
Y92	6/15/17	29.288	-90.030	M	231	8.3	ST	KS202	3	12
Y94	6/16/17	29.385	-90.048	M	213	1.8	WC	SPOT-299A	55	56
Y96	6/19/17	29.449	-89.928	M	185	2.5	WC	SPOT-299A	125	126
Y98	6/23/17	29.348	-89.988	M	256	10.2	WC	SPOT-299A	75	88
YK9	6/14/17	29.373	-90.008	F	214	5.2	WC	SPOT-299A	83	108
YN1	6/15/17	29.301	-90.045	F	186	7.8	WC	SPOT-299A	77	77
YN3	6/15/17	29.301	-90.045	F	193	7.8	ST	KS202	20	60
YN5	6/16/17	29.368	-90.049	F	214	4.9	WC	SPOT-299A	110	110
YN7	6/16/17	29.352	-90.066	F	225	5.9	ST	KS202	10	46
YN9	6/17/17	29.450	-90.070	F	222	0.7	ST	KS202	21	132
YR1	6/17/17	29.450	-90.070	F	197	0.7	WC	SPOT-299A	66	94
YR3	6/19/17	29.463	-89.919	F	234	2.3	ST	KS202	10	63
YR5	6/23/17	29.359	-90.050	F	197	6.2	ST	KS202	24	77
September 2017										
YF0	9/21/17	29.286	-90.034	M	232	18.9	WC	SPOT-299A	119	122
YF2	9/21/17	29.286	-90.034	M	238	18.9	WC	SPOT-299A	47	48

YF4	9/21/17	29.244	-90.001	M	203	19.2	WC	SPOT-299A	181	182
YX5	9/21/17	29.244	-90.001	F	245	19.2	WC	SPOT-299A	164	164

Table S2: Summary of telemetry locations and potential ranging area size for dolphins from 2011 to 2017. FB: Dolphin identification; Mean Long: Mean longitude from telemetry locations; Mean Lat: Mean latitude from telemetry locations.

FB	Sex	Group	# of included transmissions	Mean Lon.	Mean Lat.	Max. distance betw. Points (km)	PRA size (km^2)	Bandwidth (h_{ah})
August 2011								
Y00	M	Interior	290	-90.001	29.258	17.5	138	1285
Y01	F	Interior	472	-90.019	29.277	20.4	125	797
Y02	M	Interior	234	-90.116	29.188	22.9	173	1614
Y03	F	Barrier island	135	-90.042	29.227	15	75	810
Y04	M	Interior	200	-90.030	29.250	20.1	177	1400
Y07	F	Interior	431	-90.157	29.168	35.8	214	2416
Y08	M	Barrier island	136	-90.027	29.222	23	130	1700
Y09	F	Barrier island	105	-89.952	29.273	8.4	21	566
Y10	M	Interior	279	-90.094	29.299	27.8	357	2187
Y11	F	Barrier island	125	-89.928	29.281	14.9	59	1192
Y12	M	Interior	112	-90.053	29.238	14	102	1025
Y13	F	Interior	530	-89.999	29.326	24.7	151	735
Y14	M	Interior	564	-90.014	29.426	27.1	193	1169
Y15	F	Interior	69	-90.006	29.278	17.9	188	1236
Y16	M	Barrier island	154	-89.954	29.264	11.6	43	827
Y17	F	Barrier island	492	-89.973	29.260	13.5	36	592
Y18	M	Interior	524	-90.006	29.300	35.7	267	1125
Y19	F	Barrier island	61	-89.972	29.256	10.3	29	607
Y20	M	Interior	356	-90.019	29.293	29.7	282	1386
Y22	M	Interior	397	-90.008	29.344	23.2	175	1164
Y25	F	Interior	436	-90.025	29.431	25.4	149	996
Y27	F	Interior	533	-90.020	29.433	34.7	219	1101
Y33	F	Barrier island	302	-90.011	29.218	22.5	47	800
Y37	F	Interior	520	-90.056	29.398	21.2	232	1682
June 2013								
Y38	M	Barrier island	580	-89.829	29.335	18.5	99	865
Y39	F	Barrier island	303	-90.033	29.217	17.3	42	475
Y40	M	Barrier island	545	-89.780	29.312	30.7	66	848

Y42	M	Barrier island	420	-89.786	29.314	30.4	108	1337
Y44	M	Barrier island	376	-89.748	29.303	30	115	1188
Y46	M	Barrier island	287	-89.949	29.269	8.6	23	519
Y65	F	Barrier island	348	-89.875	29.324	14.6	69	773
Y67	F	Barrier island	281	-89.923	29.287	8.3	19	564
Y69	F	Barrier island	321	-89.950	29.276	19.6	71	1042
June 2014								
Y71	F	Barrier island	111	-90.058	29.202	16.3	38	851
Y75	F	Interior	203	-90.027	29.259	17.6	147	905
Y81	F	Barrier island	305	-89.936	29.283	12.9	59	1133
Y83	F	Barrier island	252	-89.960	29.273	13.6	63	1046
Y85	F	Barrier island	113	-90.050	29.208	32.4	96	895
Y91	F	Barrier island	64	-90.049	29.221	24.3	107	1289
Y97	F	Interior	55	-90.030	29.344	20.9	186	1399
Y99	F	Barrier island	80	-90.035	29.219	25.9	148	1913
YA1	F	Interior	305	-90.037	29.231	34.7	216	1588
YA3	F	Interior	185	-90.082	29.225	37.3	347	2619
YA5	F	Barrier island	438	-90.027	29.220	20.9	85	1211
July 2016								
Y79	F	Barrier island	109	-89.997	29.251	15.2	96	845
Y80	M	Barrier island	241	-89.831	29.331	18.2	102	973
Y88	M	Interior	138	-90.029	29.269	16	180	1350
YA7	F	Interior	299	-90.039	29.246	15	124	1010
YF1	F	Barrier island	230	-89.988	29.261	9.7	56	956
YJ1	F	Barrier island	58	-89.982	29.245	13.7	36	759
YJ9	F	Barrier island	107	-90.036	29.225	24.4	83	1034
YK1	F	Barrier island	252	-90.023	29.235	13.6	63	1000
YK3	F	Barrier island	393	-90.008	29.235	16.6	61	838
YK5	F	Barrier island	158	-90.044	29.216	13.8	44	581
June 2017								
Y94	M	Interior	137	-90.043	29.367	19.1	125	1213
Y96	M	Interior	597	-89.903	29.479	13.1	53	946
Y98	M	Interior	27	-90.089	29.383	33	482	2526
YK9	F	Interior	106	-89.977	29.387	26.4	379	1969
YN1	F	Interior	164	-90.116	29.310	14	86	977
YN3	F	Interior	134	-90.080	29.269	15.3	134	996
YN5	F	Interior	253	-90.076	29.350	18.8	151	1379
YN7	F	Interior	287	-90.055	29.350	7	33	684
YN9	F	Interior	22	-90.014	29.434	26.3	193	1307
YR1	F	Interior	448	-90.028	29.427	26.2	266	1360

YR3	F	Interior	13	-89.880	29.514	20.6	123	1624
YR5	F	Interior	24	-90.015	29.344	19	193	1632
September 2017								
YF0	M	Interior	165	-90.085	29.212	19.4	122	1040
YF2	M	Interior	13	-90.097	29.196	20	142	1416
YF4	M	Barrier island	35	-90.051	29.197	28.3	69	1176
YX5	F	Barrier island	616	-90.050	29.198	28.4	78	1260

Table S3: Comparing PRA sizes

Group	Sex	# of dolphins	Mean lat.	Median PRA size (km^2)	Median tag duration (days)	PRA size : tag duration (IQR)
Interior	F	15	29.3213	187	117	1.30 (0.95 - 1.83)
Interior	M	13	29.3103	175	122	1.32 (1.00 - 2.39)
Island	F	20	29.2436	58	77	0.73 (0.43 - 1.05)
Island	M	9	29.2837	103	110	0.66 (0.38 - 1.16)
Interior	All	28	29.3162	178	120	1.30 (0.98 - 2.08)
Island	All	29	29.2560	69	86	0.71 (0.42 - 1.10)

Table S4: Dolphins with at least two days of low salinity exposure in the week prior to health assessments. These dolphins represent the “low salinity exposure cohort”. PRA: Potential ranging area.

FB	Sex	Effort	Capture latitude (N°)	Mean telemetry latitude (N°)	Average daily PRA median salinity (ppt)	Days PRA median below 5 ppt
YR3	F	Jun 2017	29.463	29.509	4.9	6
YN9	F	Jun 2017	29.45	29.431	4.6	5
YR1	F	Jun 2017	29.45	29.427	4.8	4
Y25	F	Aug 2011	29.343	29.43	5.5	3
Y96	M	Jun 2017	29.449	29.479	5.2	3
Y98	M	Jun 2017	29.348	29.386	5.7	2
YR5	F	Jun 2017	29.359	29.344	5.7	2
