# Supplementary material for: High site-fidelity in common bottlenose dolphins despite low salinity exposure and associated indicators of compromised health
Source: PLoS One. 2021 Sep 30;16(9):e0258031. doi: 10.1371/journal.pone.0258031 (PMC8483354; doi:10.1371/journal.pone.0258031)
Supplement: S2 File — These plots combine information about each dolphin’s 1) history of photographic identification survey observations (points colored by year of observation), 2) locations received during deployment of satellite telemetry tags (black points), and 3) the potential ranging area (PRA) determined by the satellite transmissions (black contour). The dolphin ID is provided at the top of each plot along with the general pattern of usage (either Island or Interior). (PDF) [file pone.0258031.s002.pdf]

Provided as supplemental material for Takeshita et al (in submission)

Authors: Todd Speakman, Ryan Takeshita, Brian C. Balmer, Francesca Messina, Eric S. Zolman, Len Thomas, Randall S. Wells, Cynthia R. Smith, Teresa K. Rowles, and Lori H. Schwacke

Description: These plots combine information about each dolphin’s 1) history of photographic identification survey observations (points colored by year of observation), 2) locations received during deployment of satellite telemetry tags (black points), and 3) the potential ranging area (PRA) determined by the satellite transmissions (black contour). The dolphin ID is provided at the top of each plot along with the general pattern of usage (either Island or Interior).

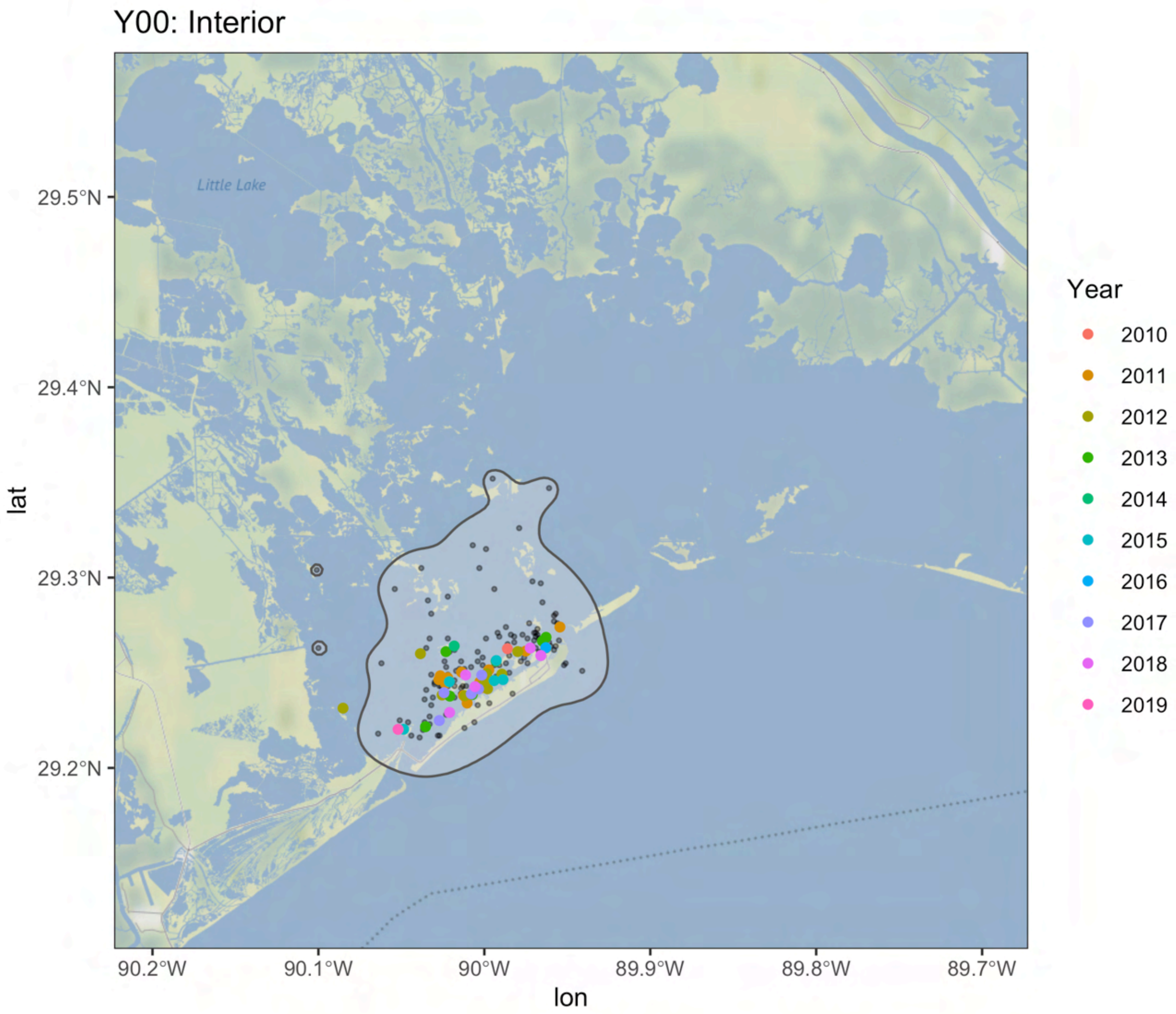
Y00: Interior
Little Lake
29.5°N
29.4°N
29.3°N
29.2°N
lat
90.2°W
90.1°W
90°W
89.9°W
89.8°W
89.7°W
lon
Year
2010
2011
2012
2013
2014
2015
2016
2017
2018
2019

Y01: Interior

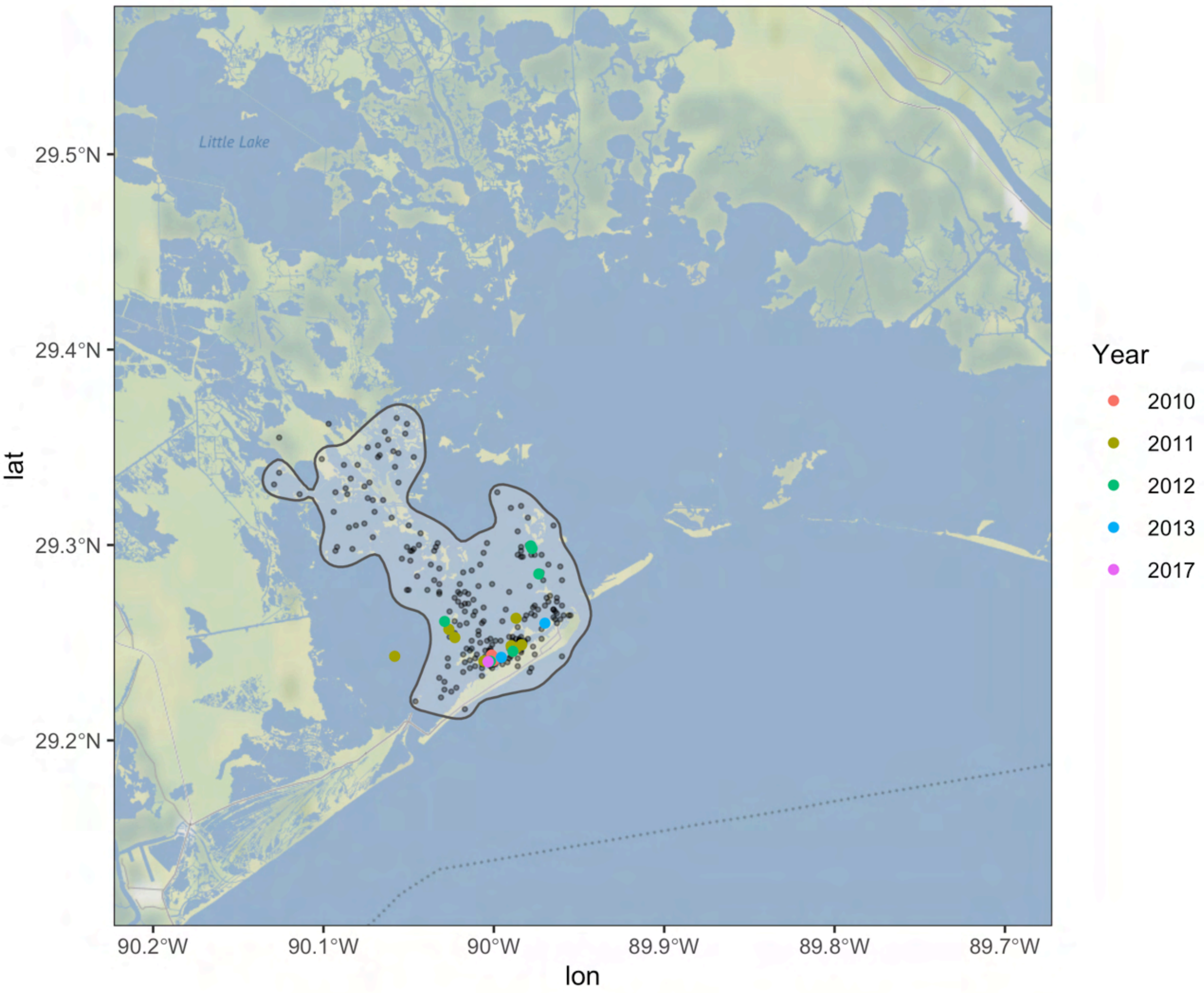

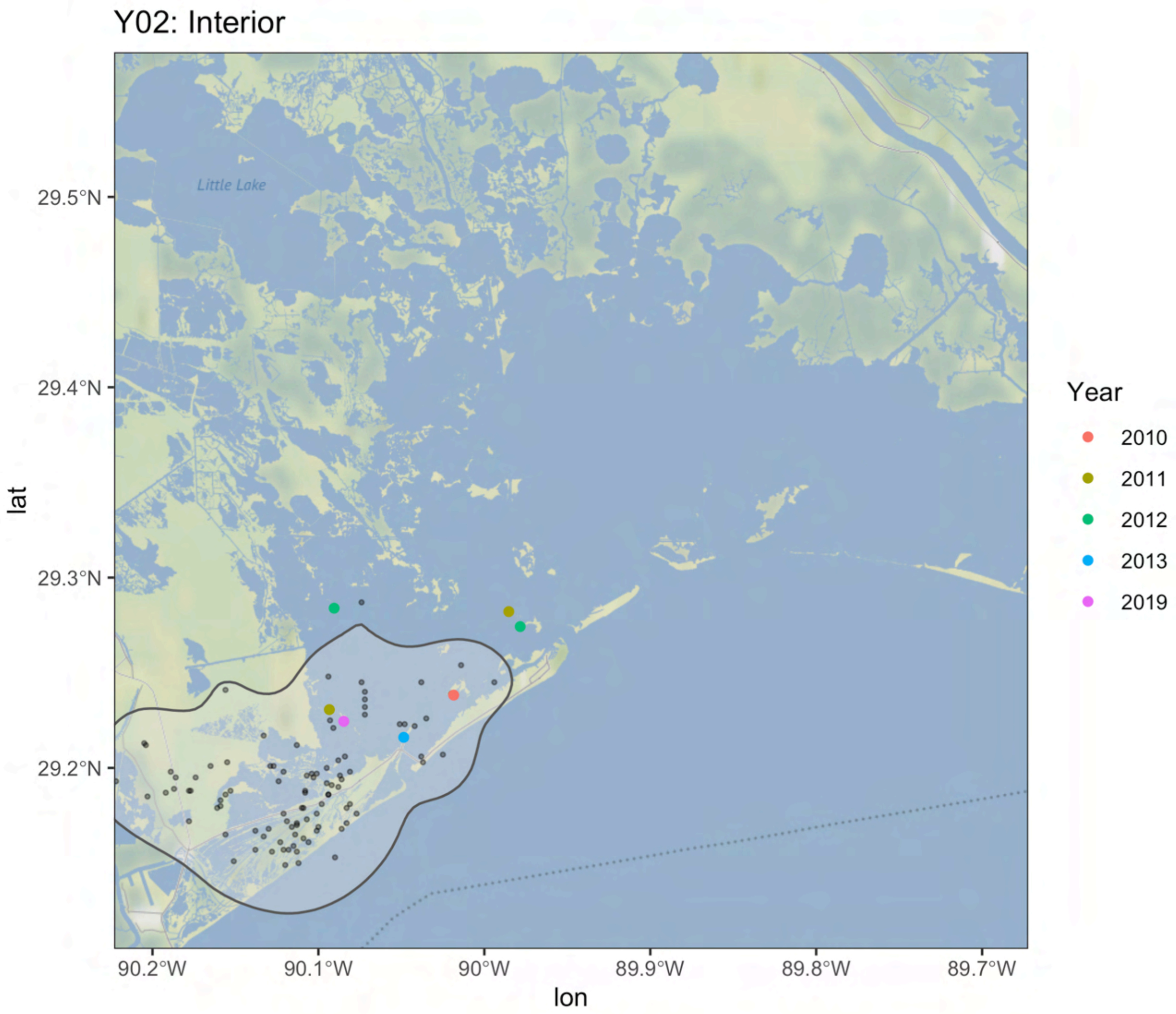
Y02: Interior
Little Lake
29.5°N
29.4°N
29.3°N
29.2°N
lat
90.2°W
90.1°W
90°W
89.9°W
89.8°W
89.7°W
lon
Year
2010
2011
2012
2013
2019

Y03: Island

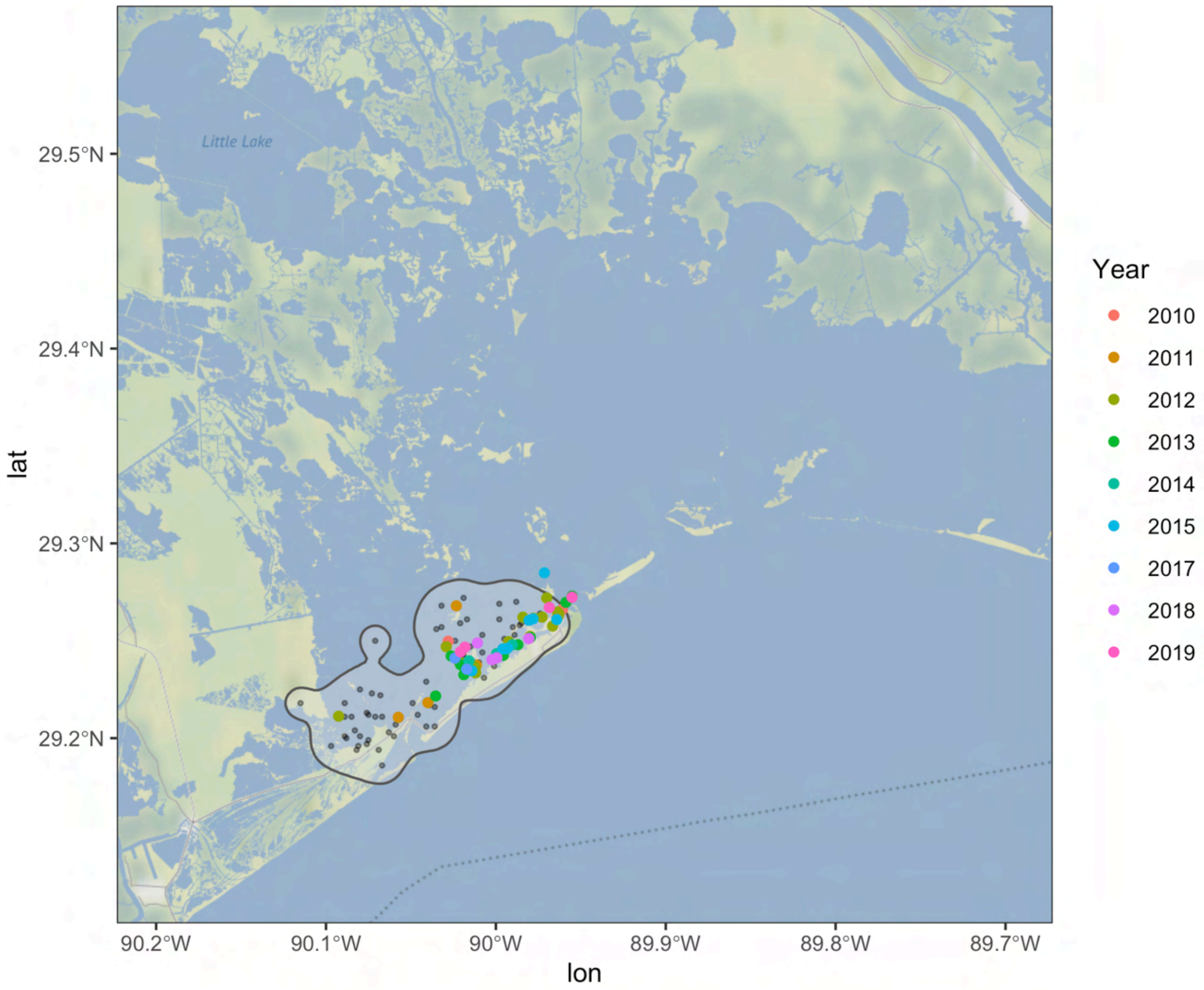
Little Lake
29.5°N
29.4°N
29.3°N
29.2°N
lat
90.2°W
90.1°W
90°W
89.9°W
89.8°W
89.7°W
lon
Year
2010
2011
2012
2013
2014
2015
2017
2018
2019

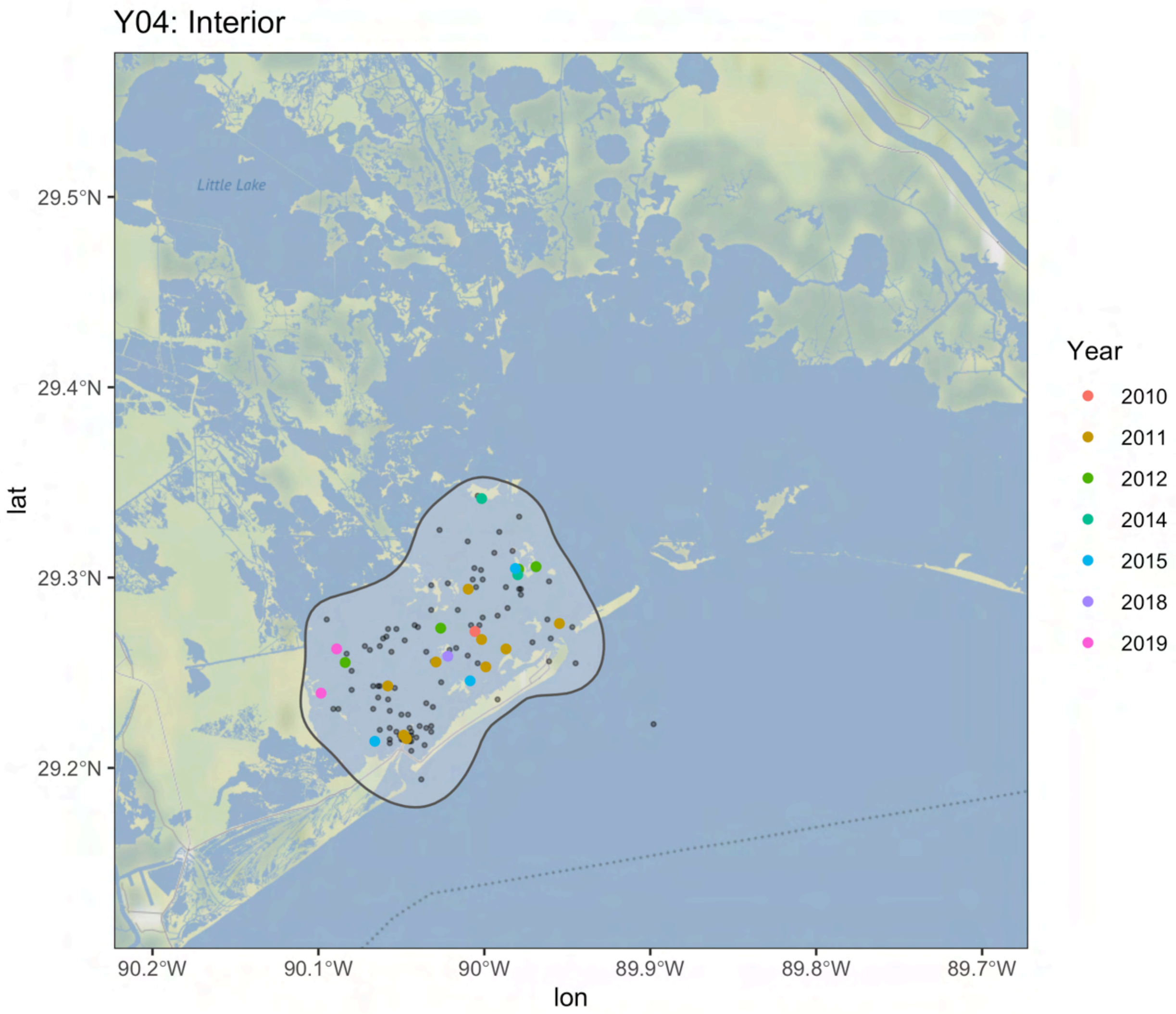

Y04: Interior
Little Lake
29.5°N
29.4°N
29.3°N
29.2°N
lat
90.2°W
90.1°W
90°W
89.9°W
89.8°W
89.7°W
lon
Year
2010
2011
2012
2014
2015
2018
2019

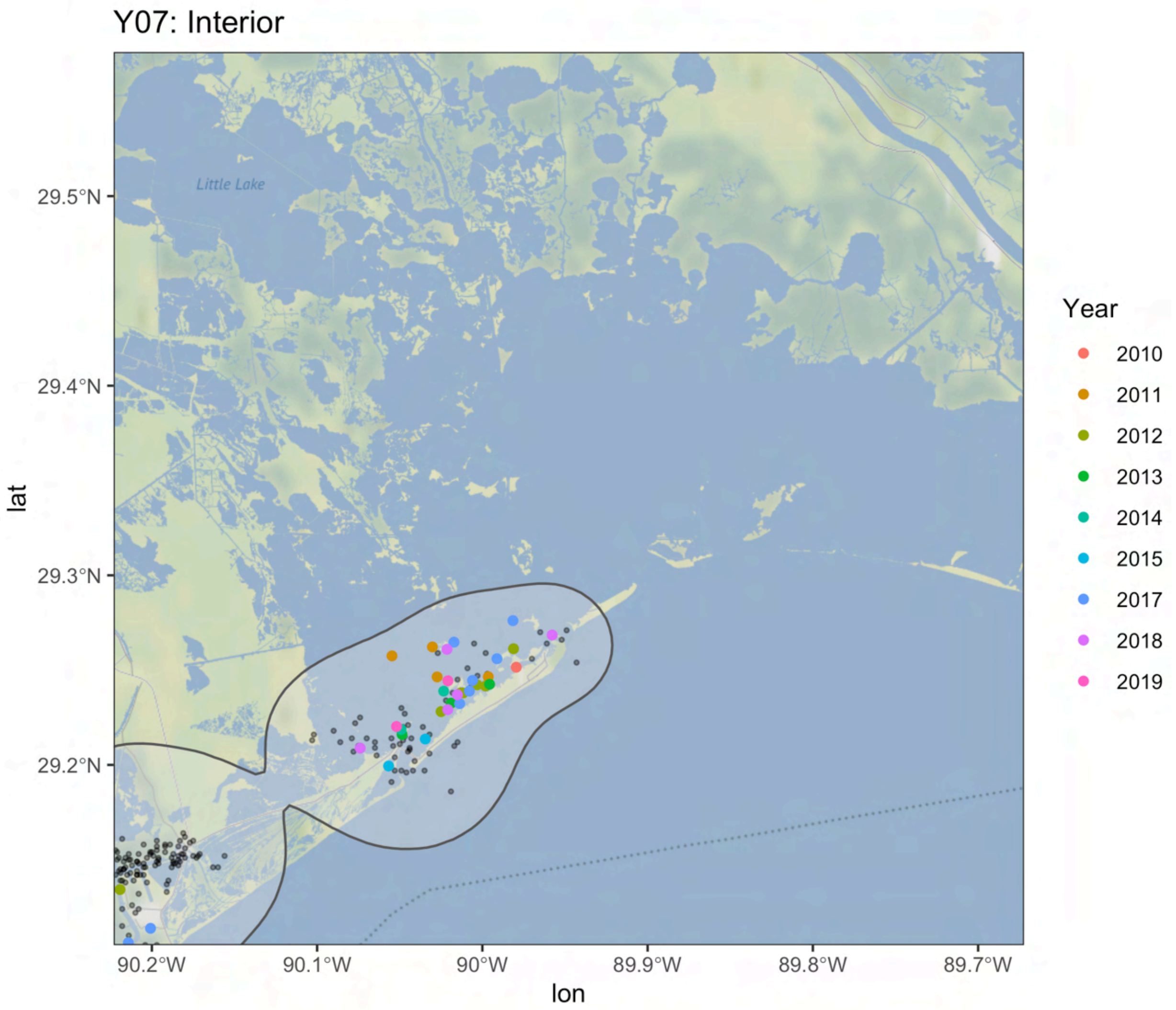
Y07: Interior
Little Lake
29.5°N
29.4°N
29.3°N
29.2°N
lat
90.2°W
90.1°W
90°W
89.9°W
89.8°W
89.7°W
lon
Year
2010
2011
2012
2013
2014
2015
2017
2018
2019

Y08: Island

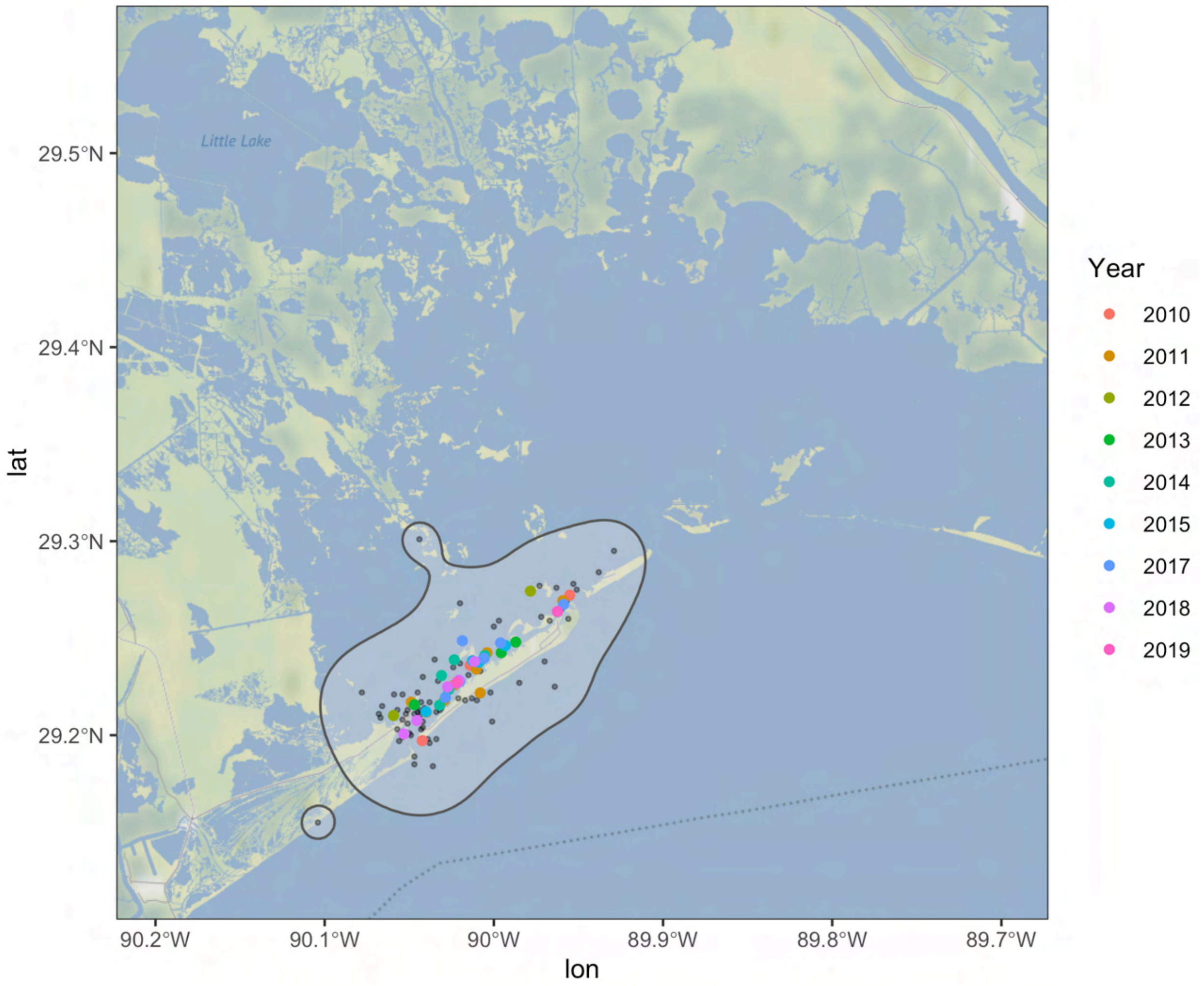

Little Lake
29.5°N
29.4°N
29.3°N
29.2°N
lat
90.2°W
90.1°W
90°W
89.9°W
89.8°W
89.7°W
lon
Year
2010
2011
2012
2013
2014
2015
2017
2018
2019

Y09: Island

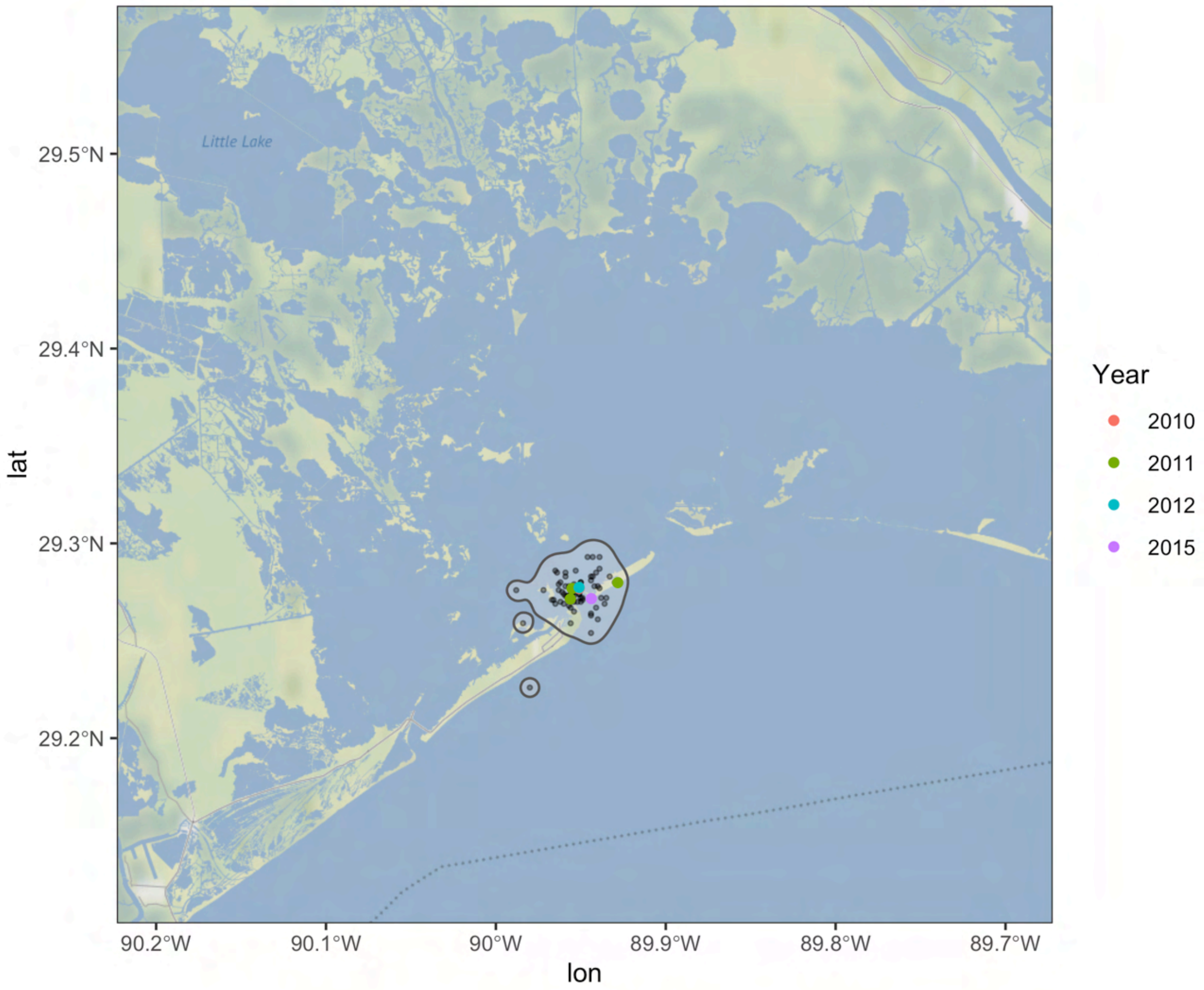

Little Lake
29.5°N
29.4°N
29.3°N
29.2°N
lat
90.2°W
90.1°W
90°W
89.9°W
89.8°W
89.7°W
lon
Year
2010
2011
2012
2015

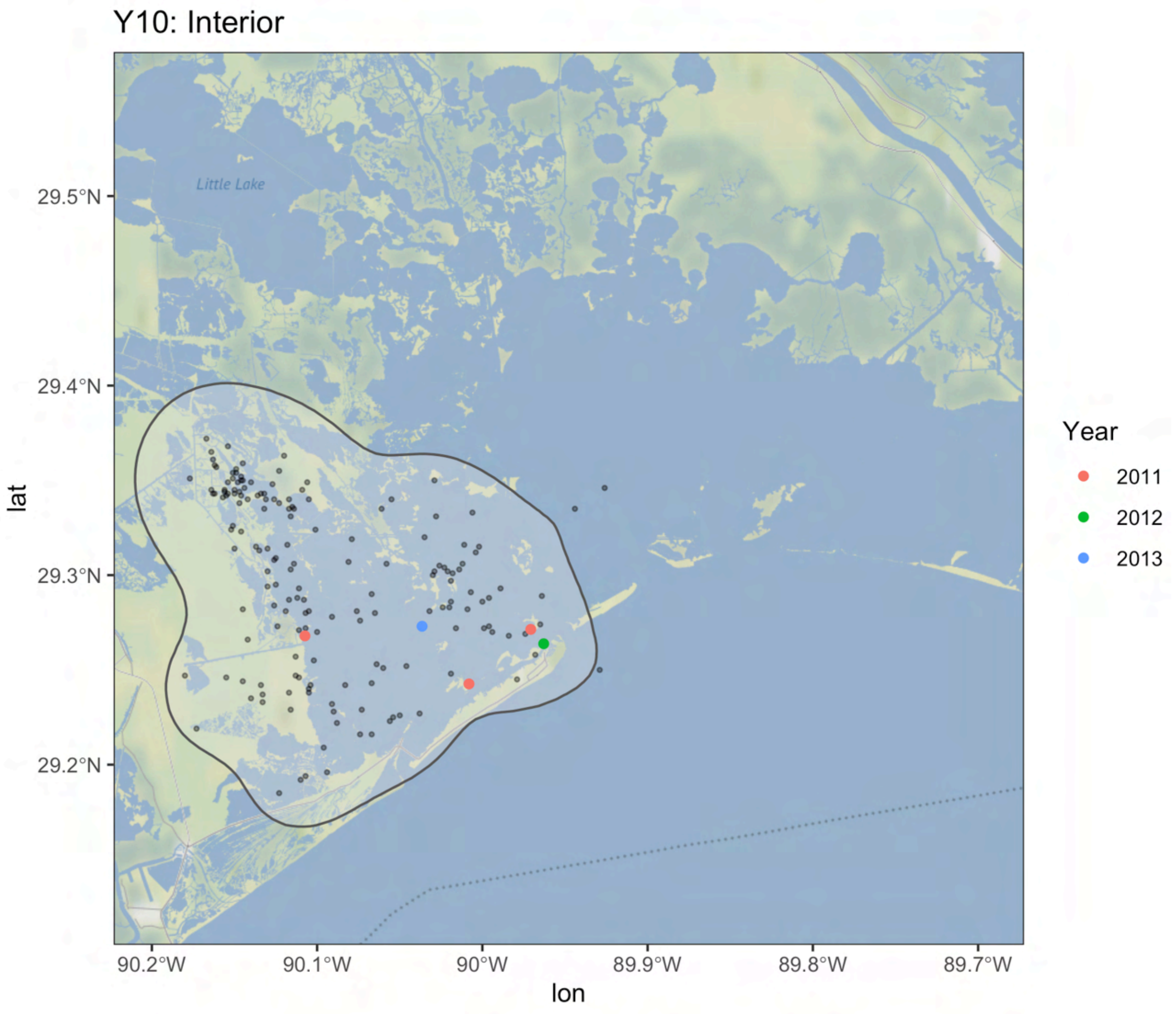

Y10: Interior
Little Lake
29.5°N
29.4°N
29.3°N
29.2°N
lat
90.2°W
90.1°W
90°W
89.9°W
89.8°W
89.7°W
lon
Year
2011
2012
2013

Y11: Island

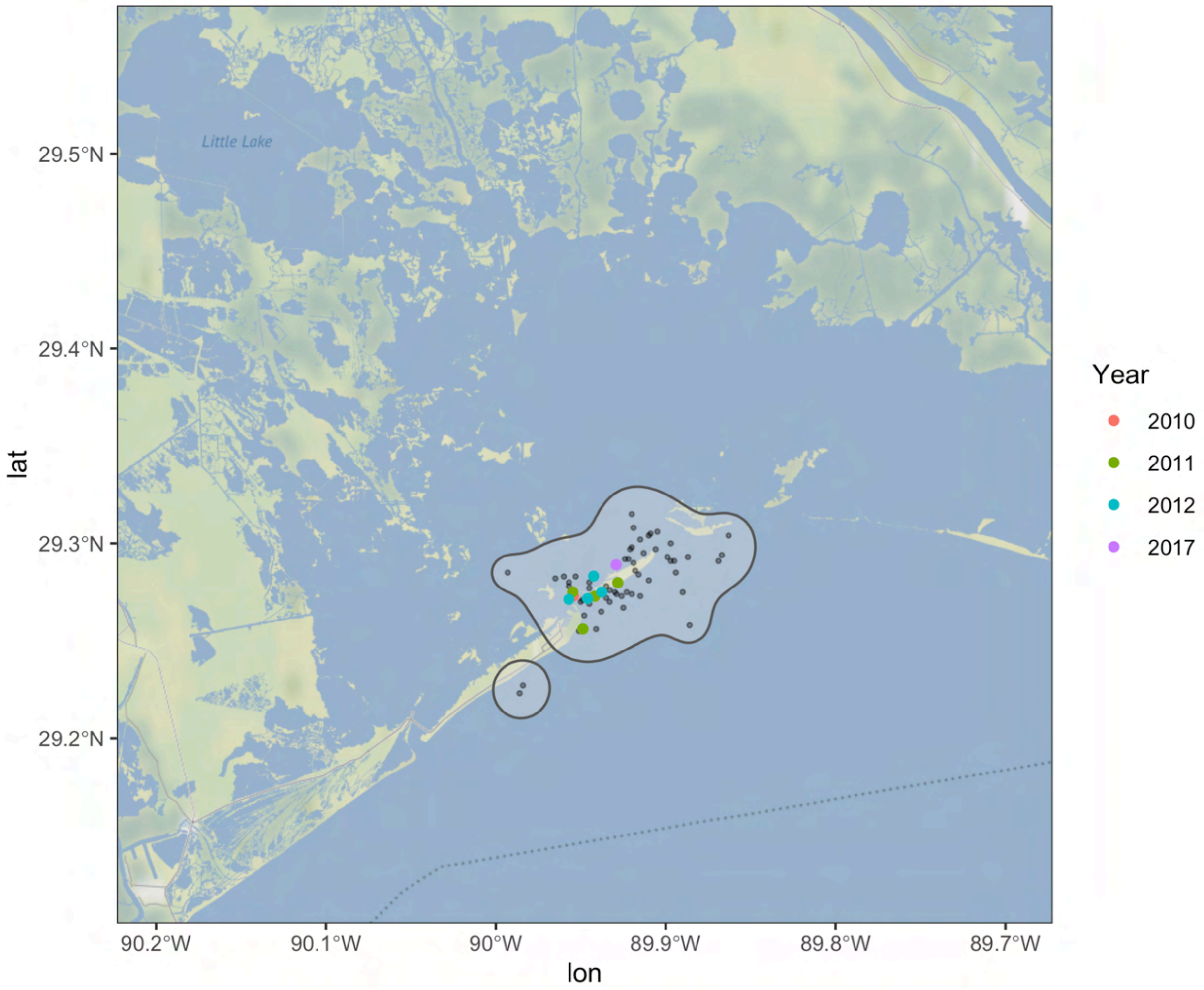

Little Lake
29.5°N
29.4°N
29.3°N
29.2°N
lat
90.2°W
90.1°W
90°W
89.9°W
89.8°W
89.7°W
lon
Year
2010
2011
2012
2017

Y12: Interior

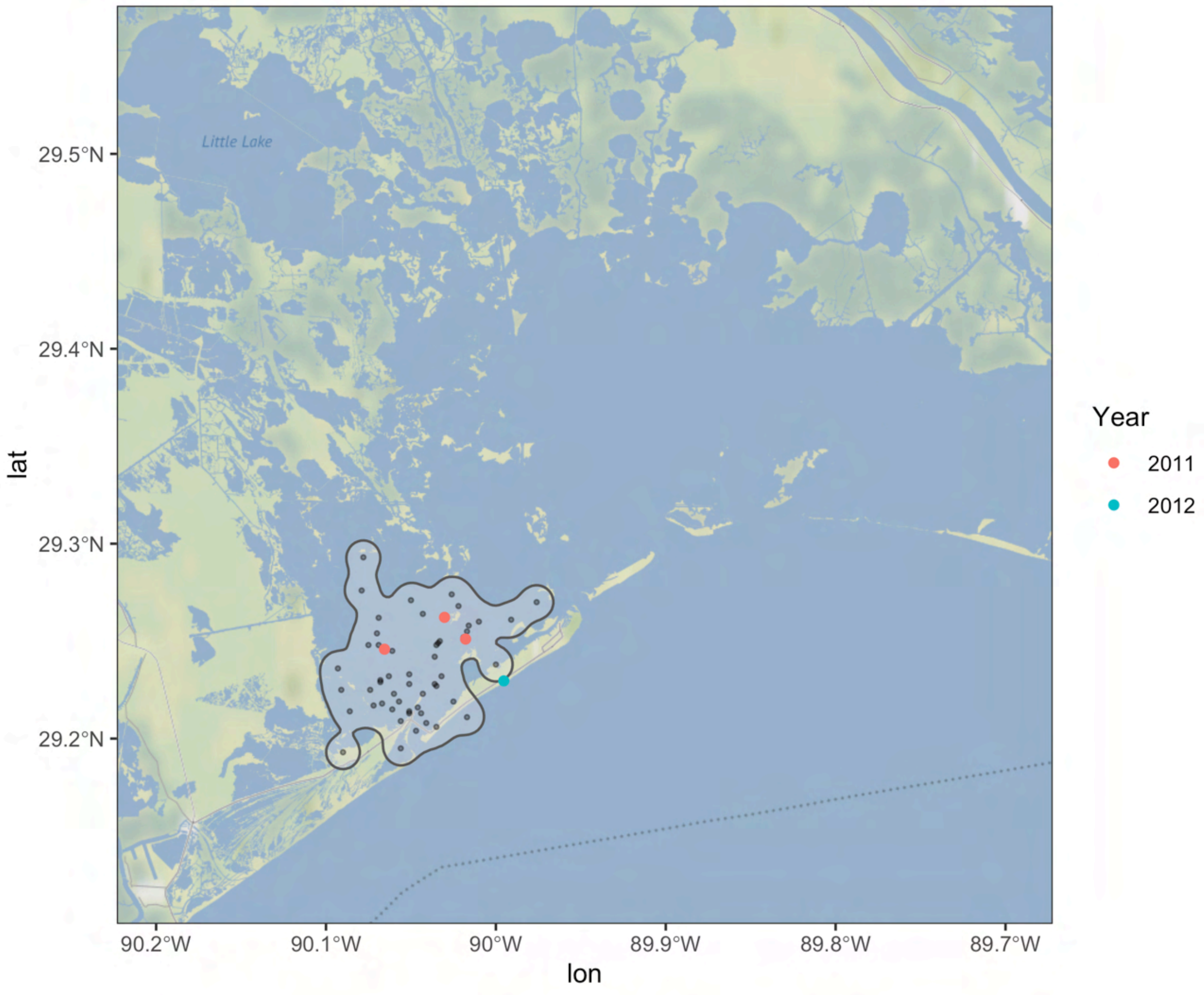

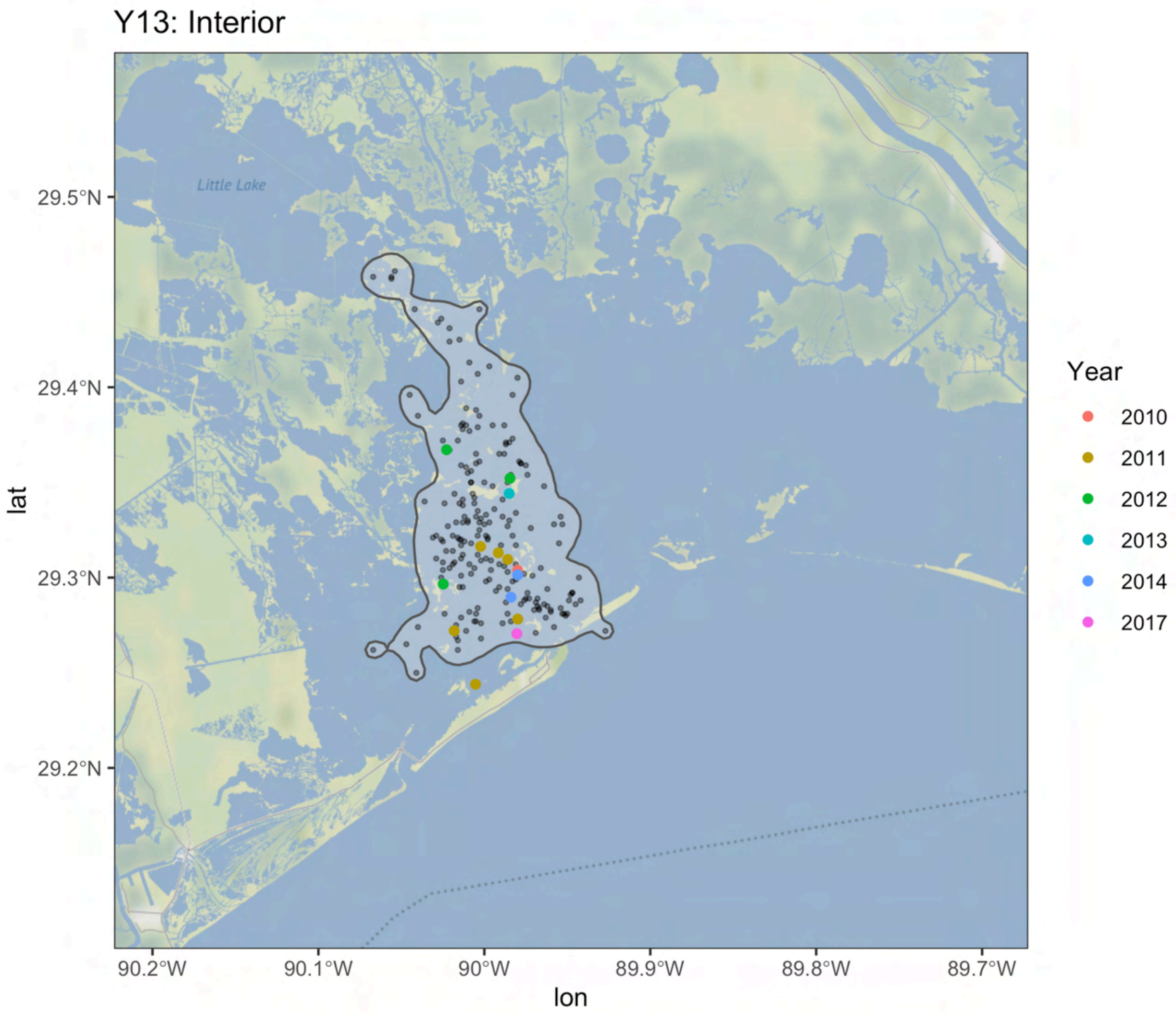
Y13: Interior
Little Lake
29.5°N
29.4°N
29.3°N
29.2°N
lat
90.2°W
90.1°W
90°W
89.9°W
89.8°W
89.7°W
lon
Year
2010
2011
2012
2013
2014
2017

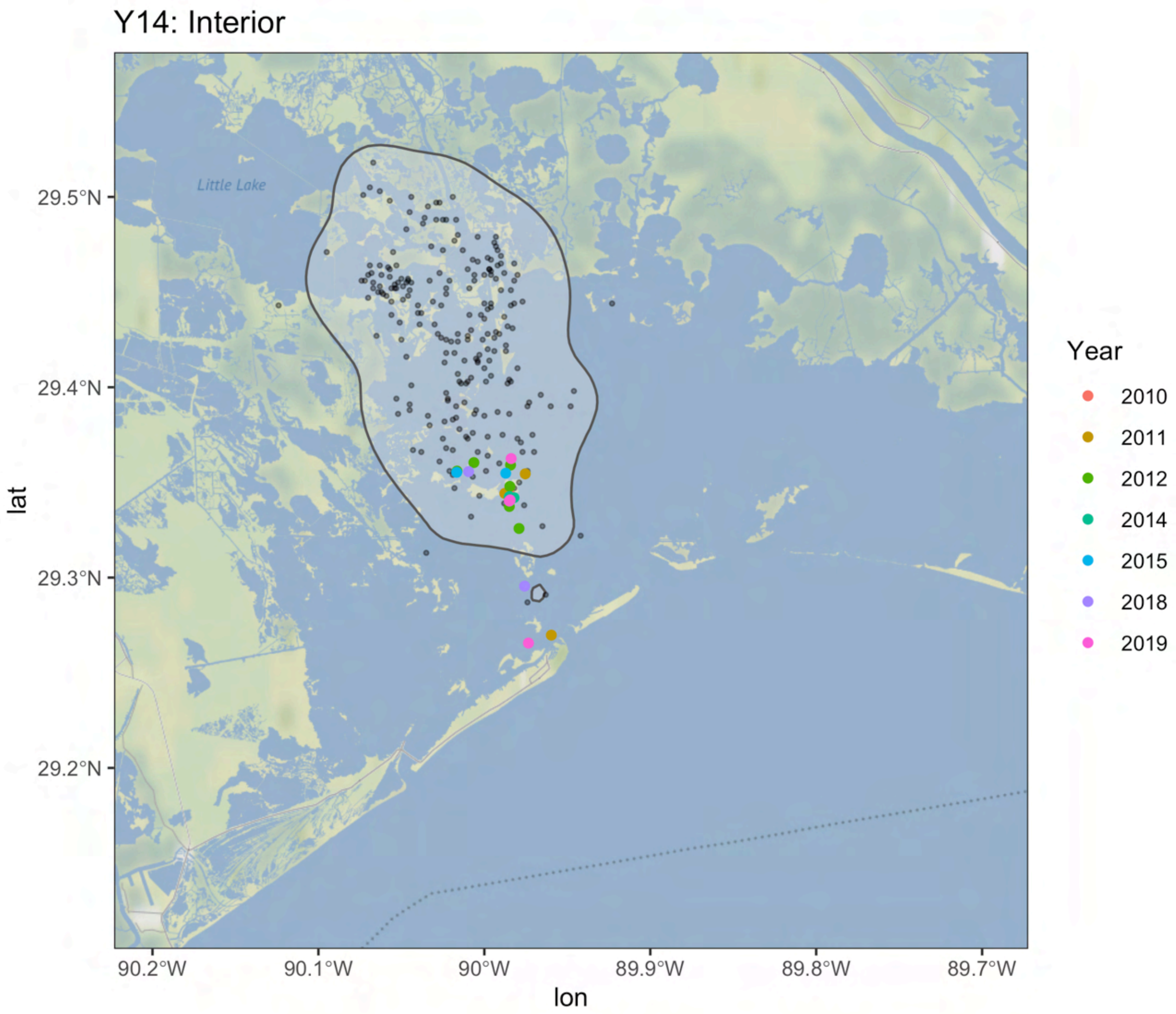

Y14: Interior
Little Lake
29.5°N
29.4°N
29.3°N
29.2°N
lat
90.2°W
90.1°W
90°W
89.9°W
89.8°W
89.7°W
lon
Year
2010
2011
2012
2014
2015
2018
2019

Y15: Interior

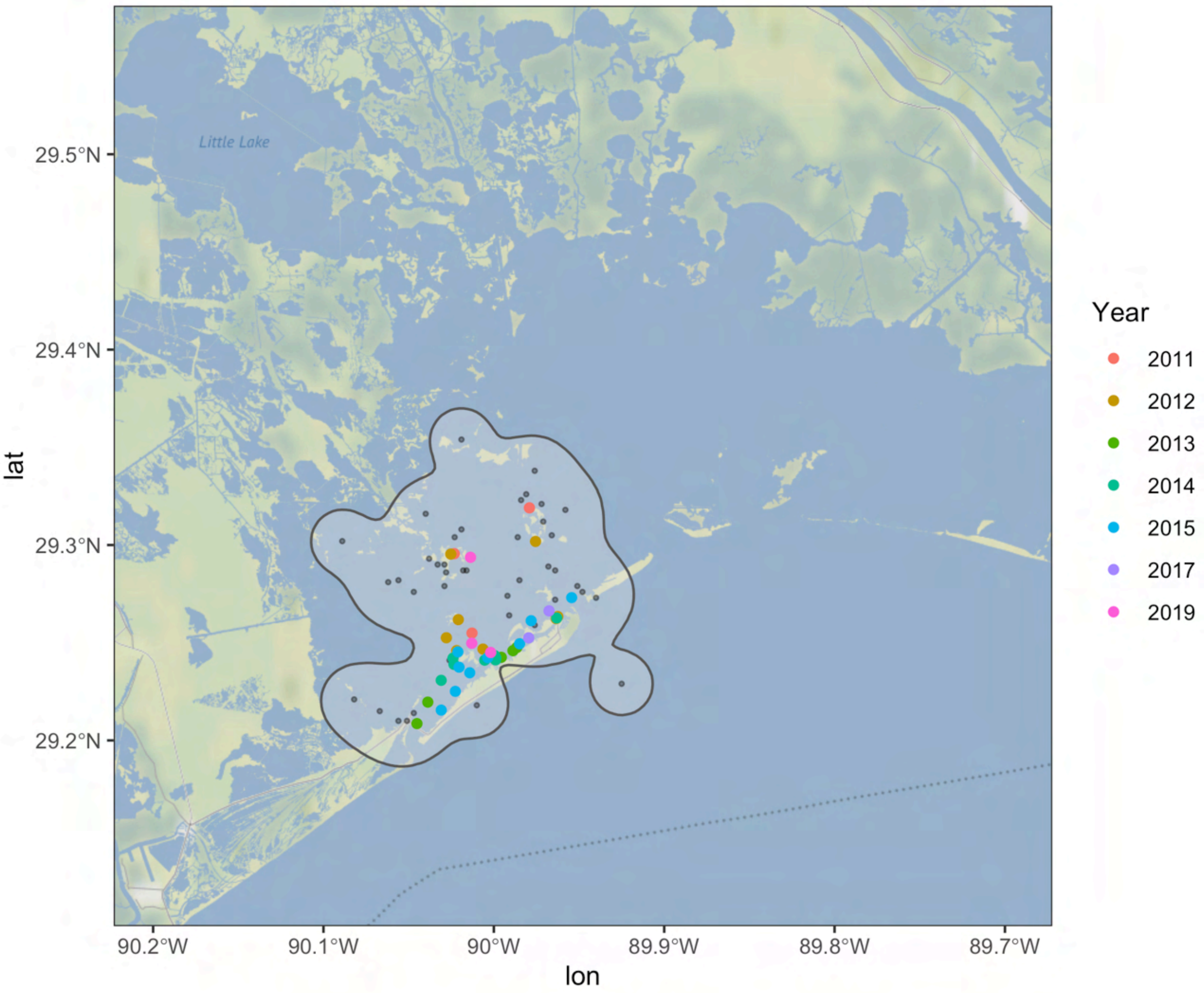

Y16: Island

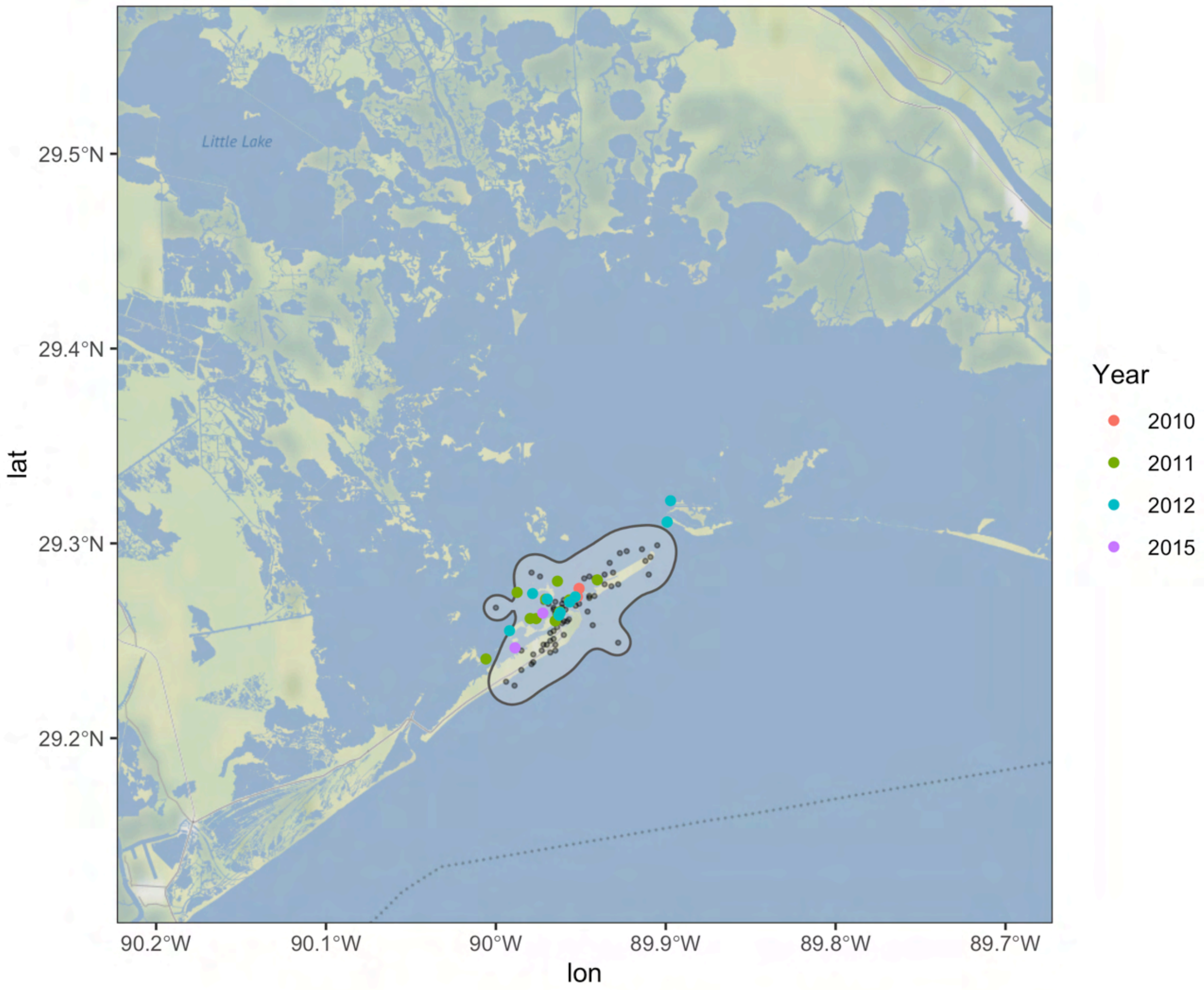

Little Lake
29.5°N
29.4°N
29.3°N
29.2°N
lat
90.2°W
90.1°W
90°W
89.9°W
89.8°W
89.7°W
lon
Year
2010
2011
2012
2015

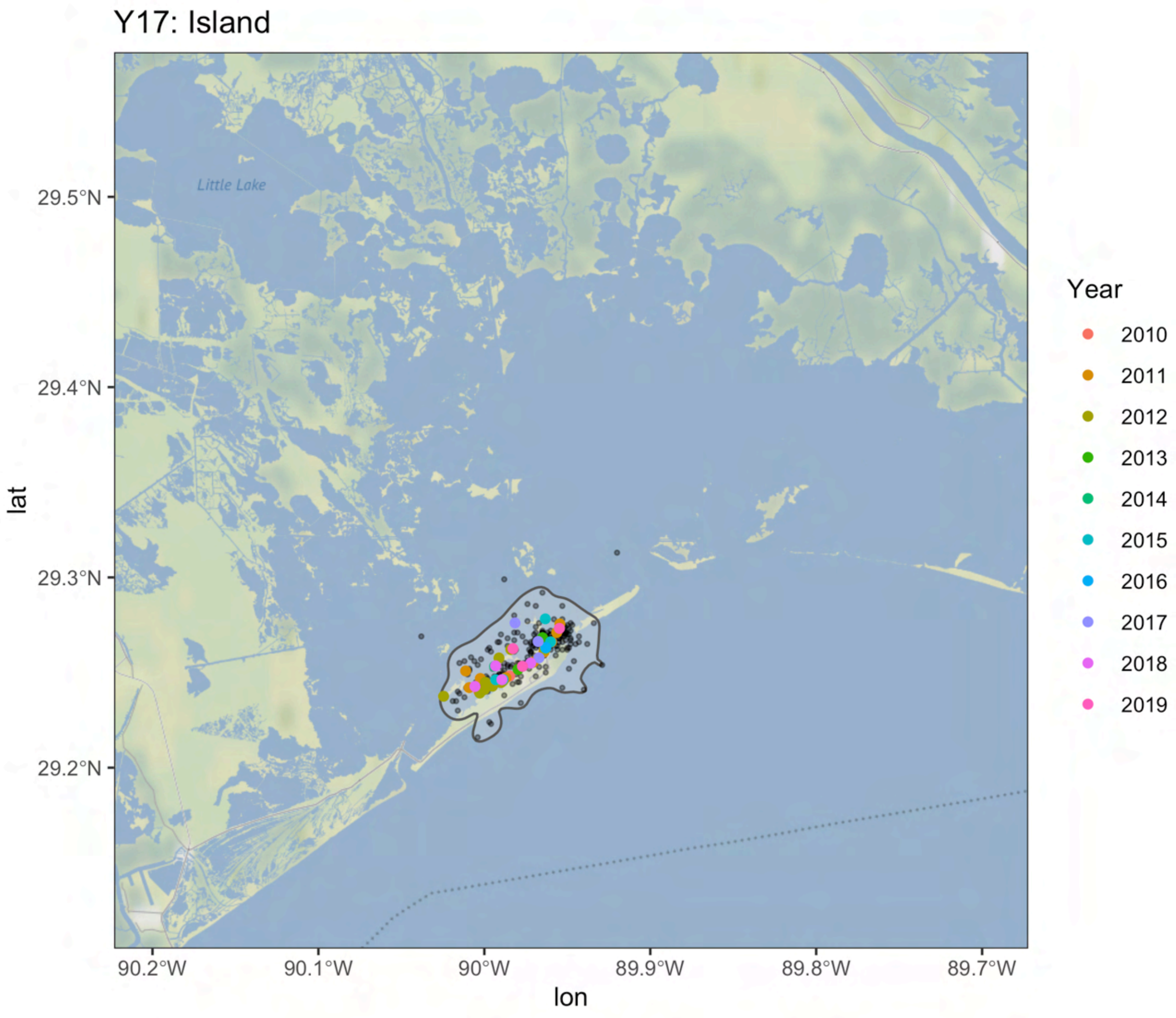
Y17: Island
Little Lake
29.5°N
29.4°N
29.3°N
29.2°N
lat
90.2°W
90.1°W
90°W
89.9°W
89.8°W
89.7°W
lon
Year
2010
2011
2012
2013
2014
2015
2016
2017
2018
2019

Y18: Interior

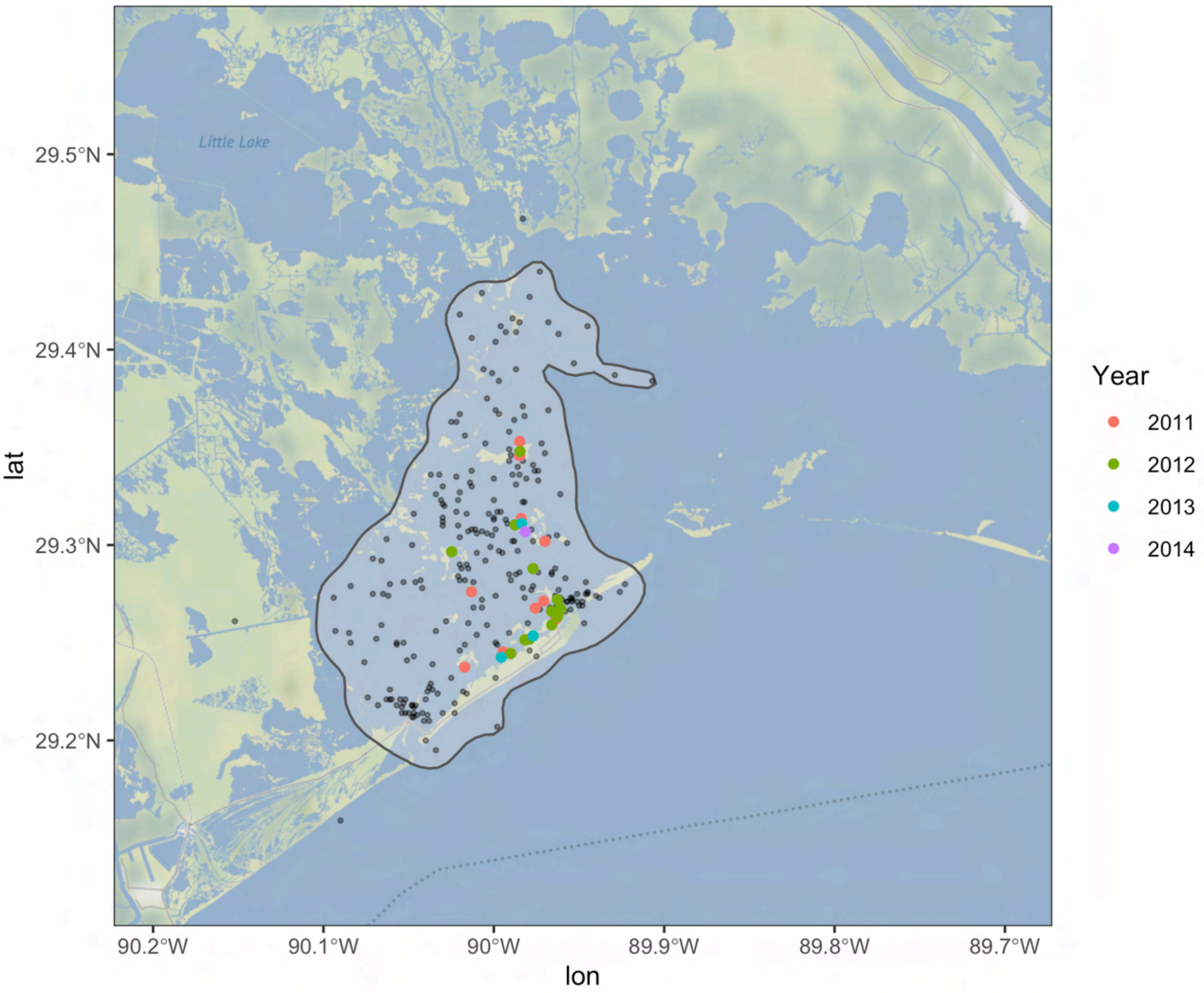

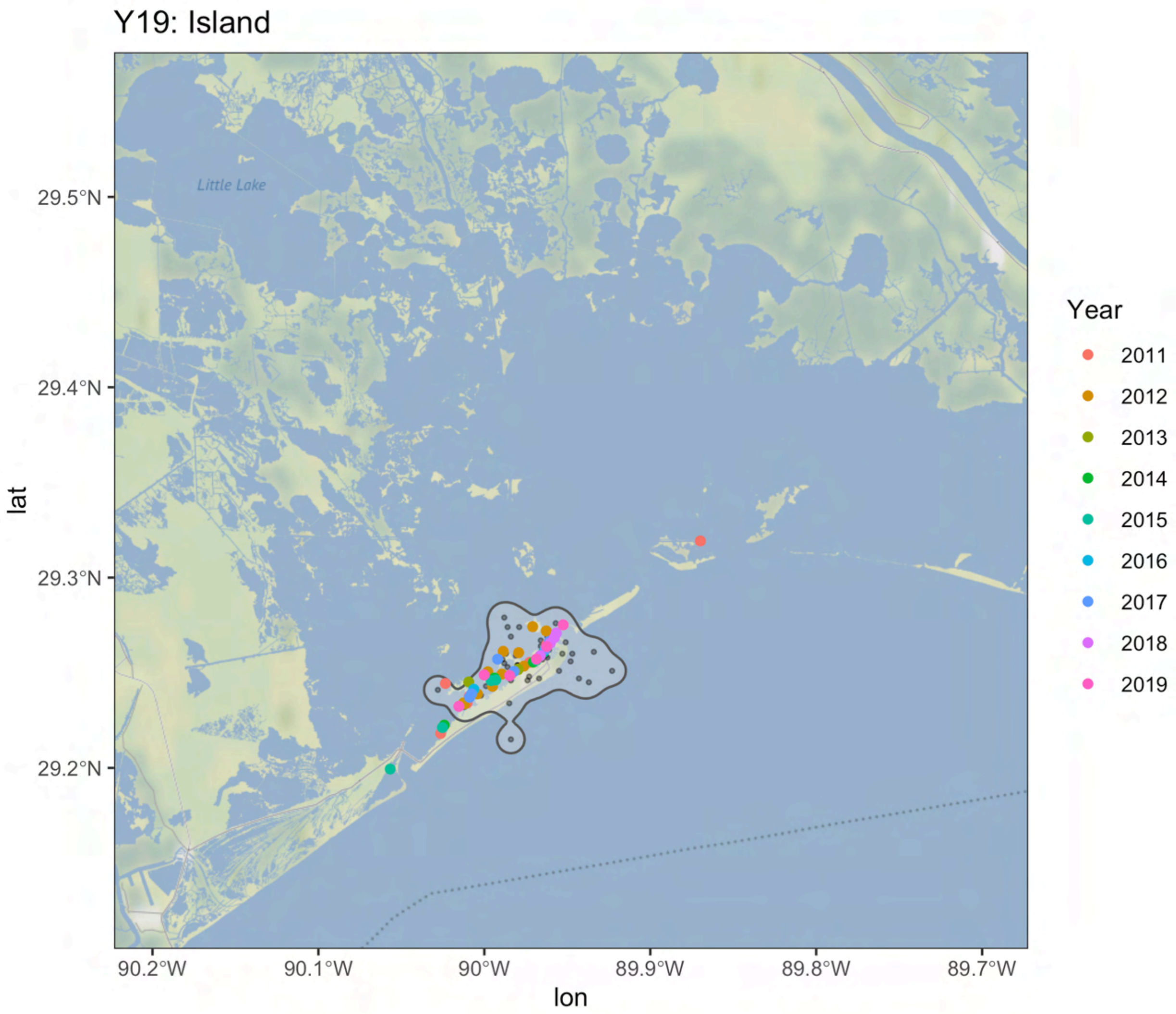
Y19: Island
Little Lake
29.5°N
29.4°N
29.3°N
29.2°N
lat
90.2°W
90.1°W
90°W
89.9°W
89.8°W
89.7°W
lon
Year
2011
2012
2013
2014
2015
2016
2017
2018
2019

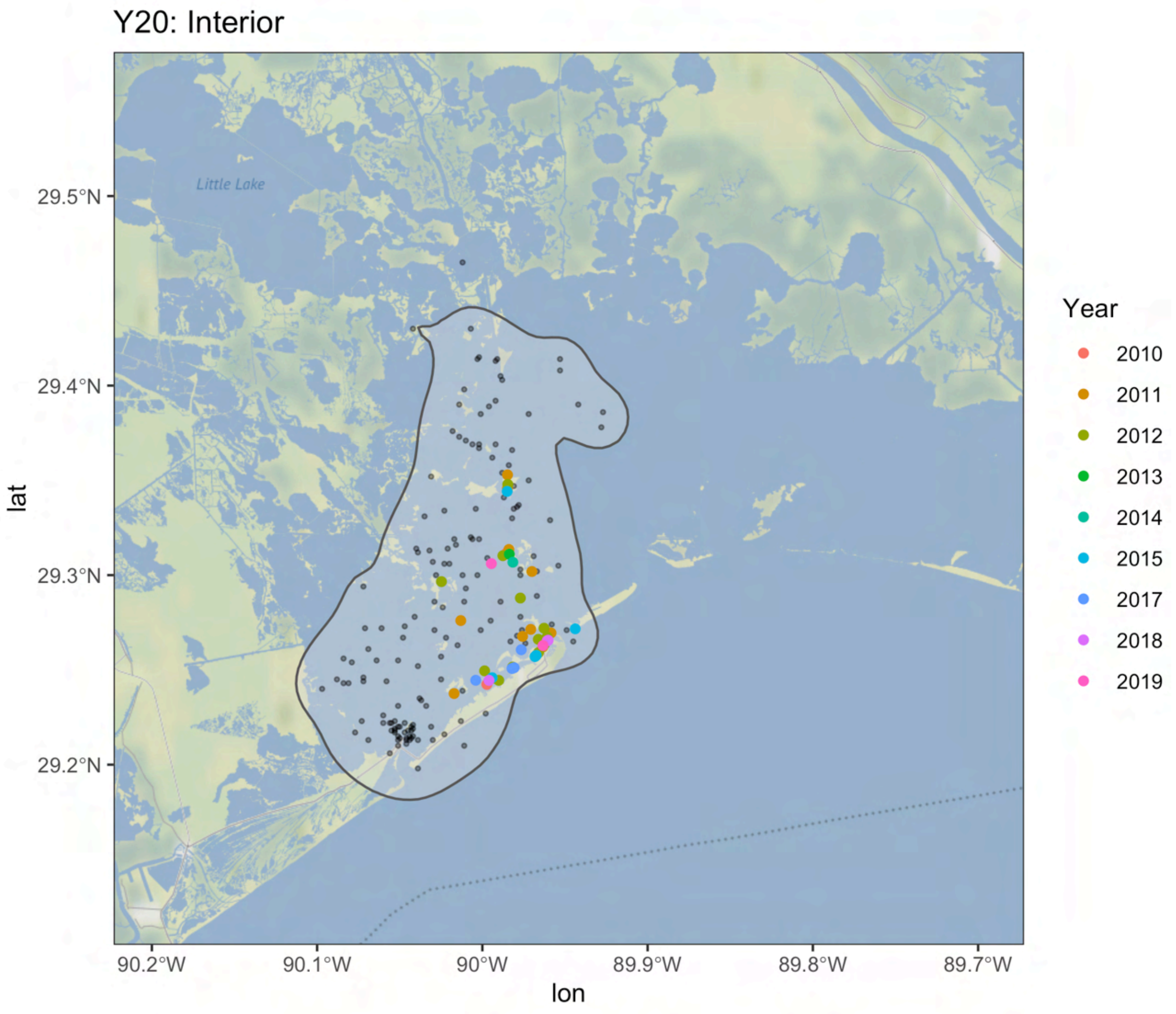
Y20: Interior
Little Lake
29.5°N
29.4°N
29.3°N
29.2°N
lat
90.2°W
90.1°W
90°W
89.9°W
89.8°W
89.7°W
lon
Year
2010
2011
2012
2013
2014
2015
2017
2018
2019

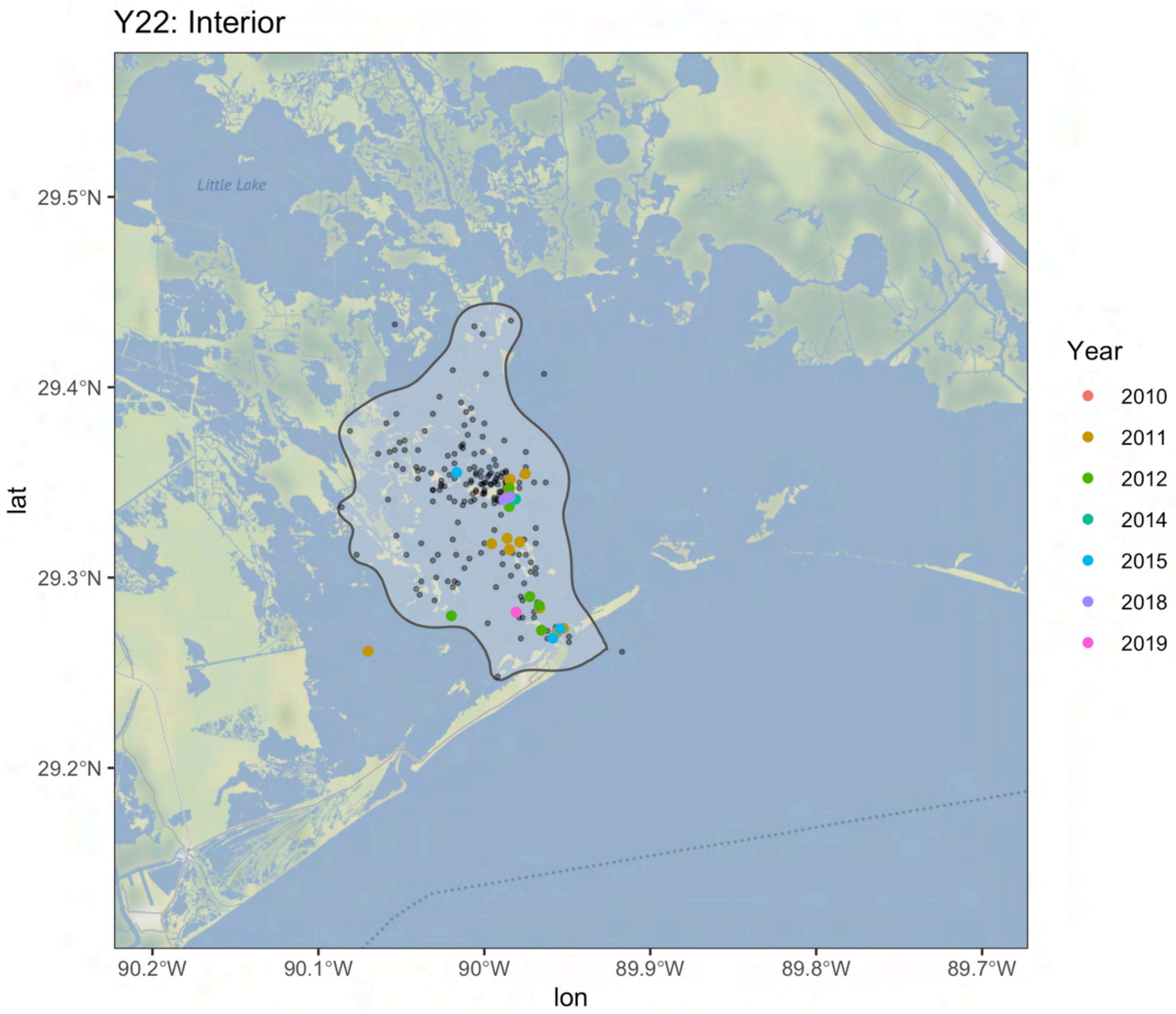
Y22: Interior
Little Lake
29.5°N
29.4°N
29.3°N
29.2°N
lat
90.2°W
90.1°W
90°W
89.9°W
89.8°W
89.7°W
lon
Year
2010
2011
2012
2014
2015
2018
2019

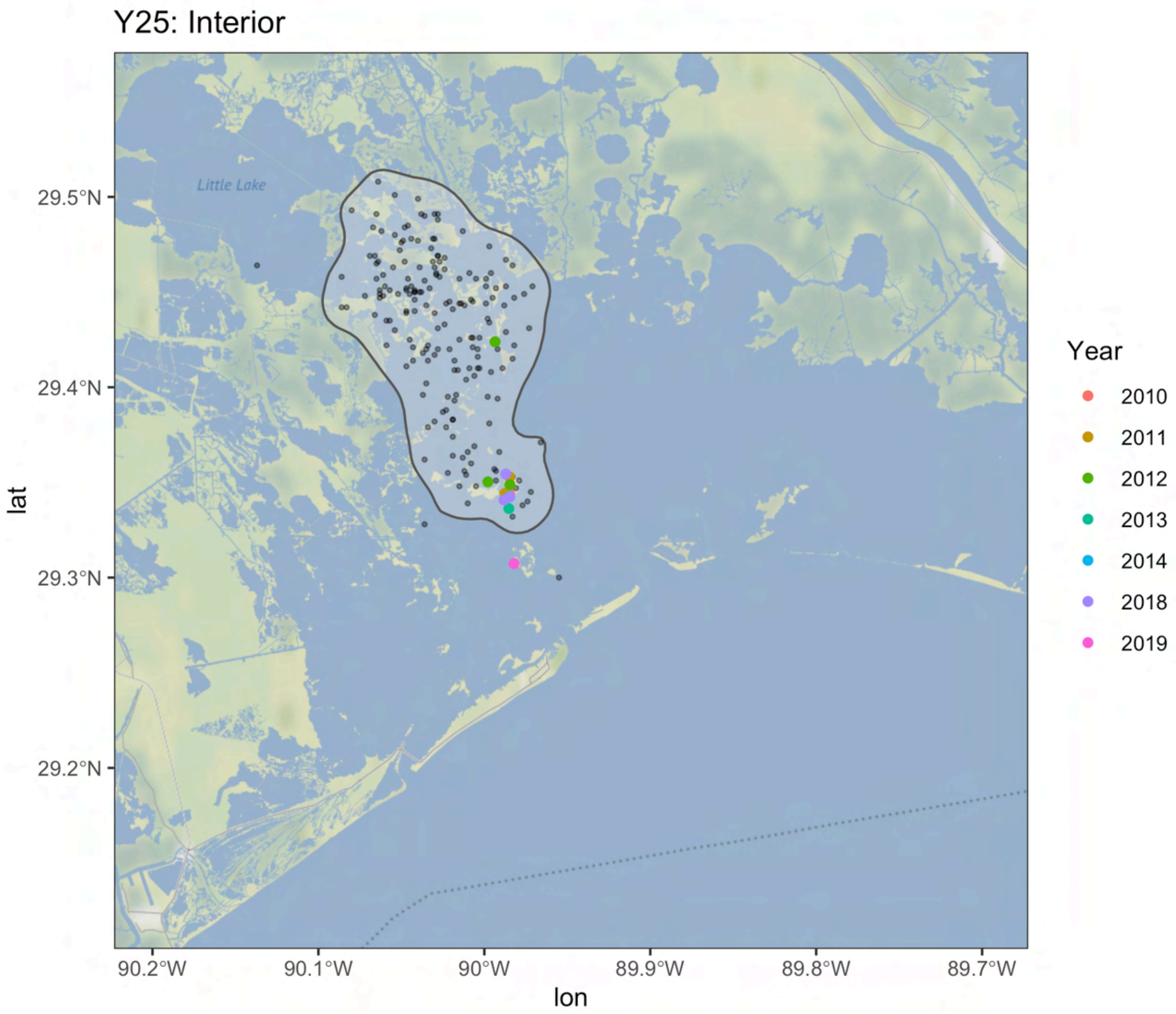
Y25: Interior
Little Lake
29.5°N
29.4°N
29.3°N
29.2°N
lat
90.2°W
90.1°W
90°W
89.9°W
89.8°W
89.7°W
lon
Year
2010
2011
2012
2013
2014
2018
2019

Y27: Interior

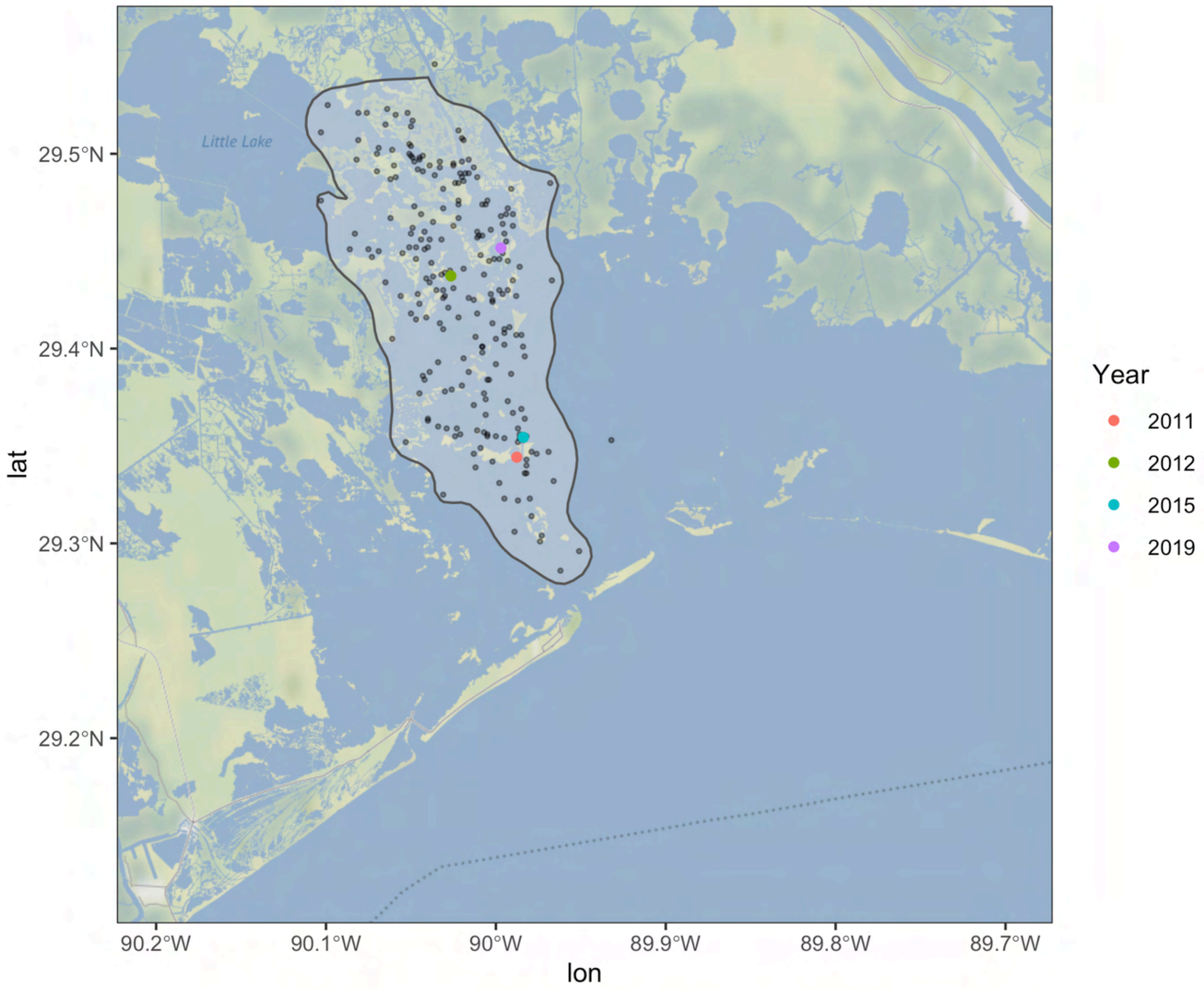

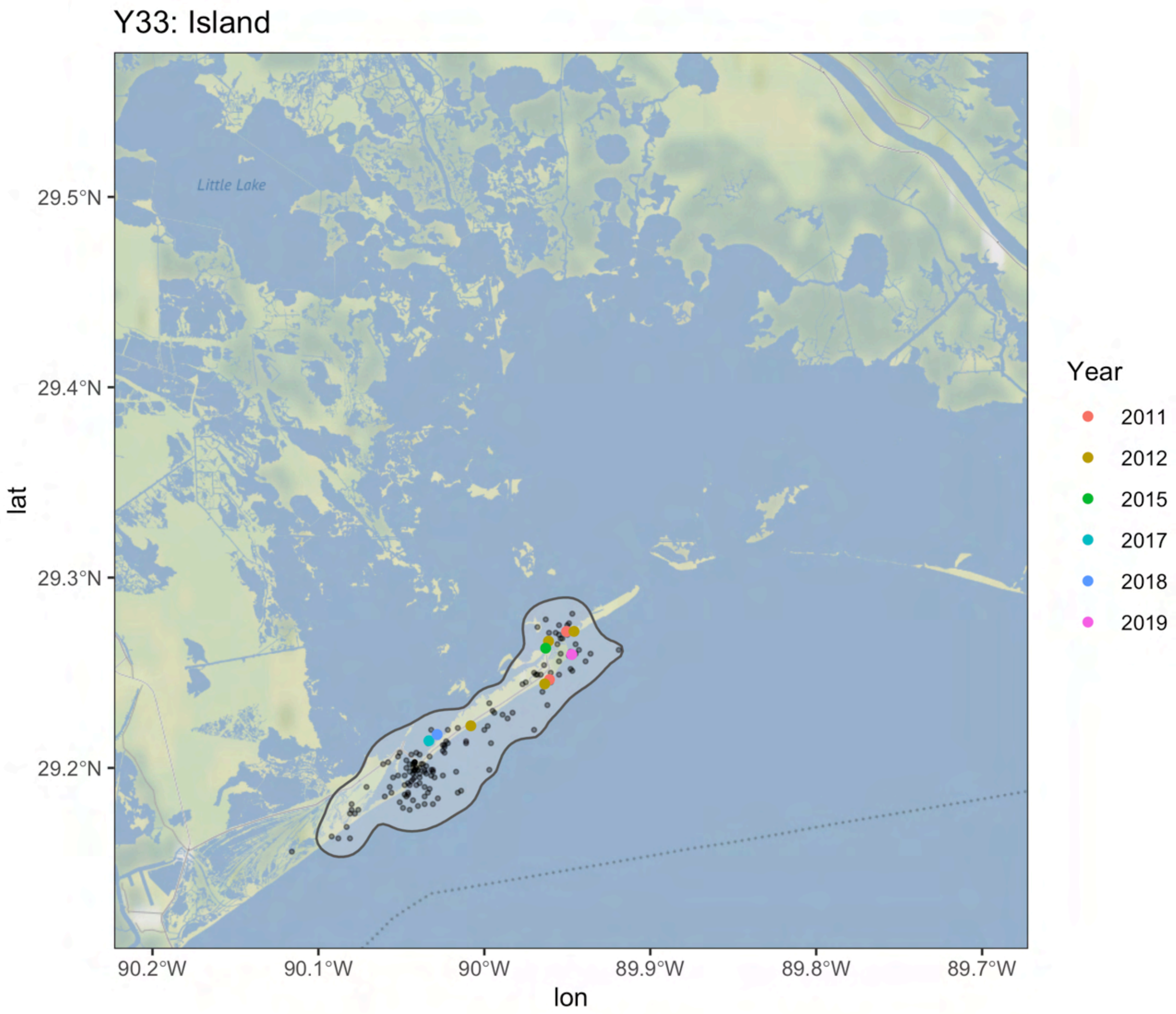
Y33: Island
Little Lake
29.5°N
29.4°N
29.3°N
29.2°N
lat
90.2°W
90.1°W
90°W
89.9°W
89.8°W
89.7°W
lon
Year
2011
2012
2015
2017
2018
2019

Y37: Interior

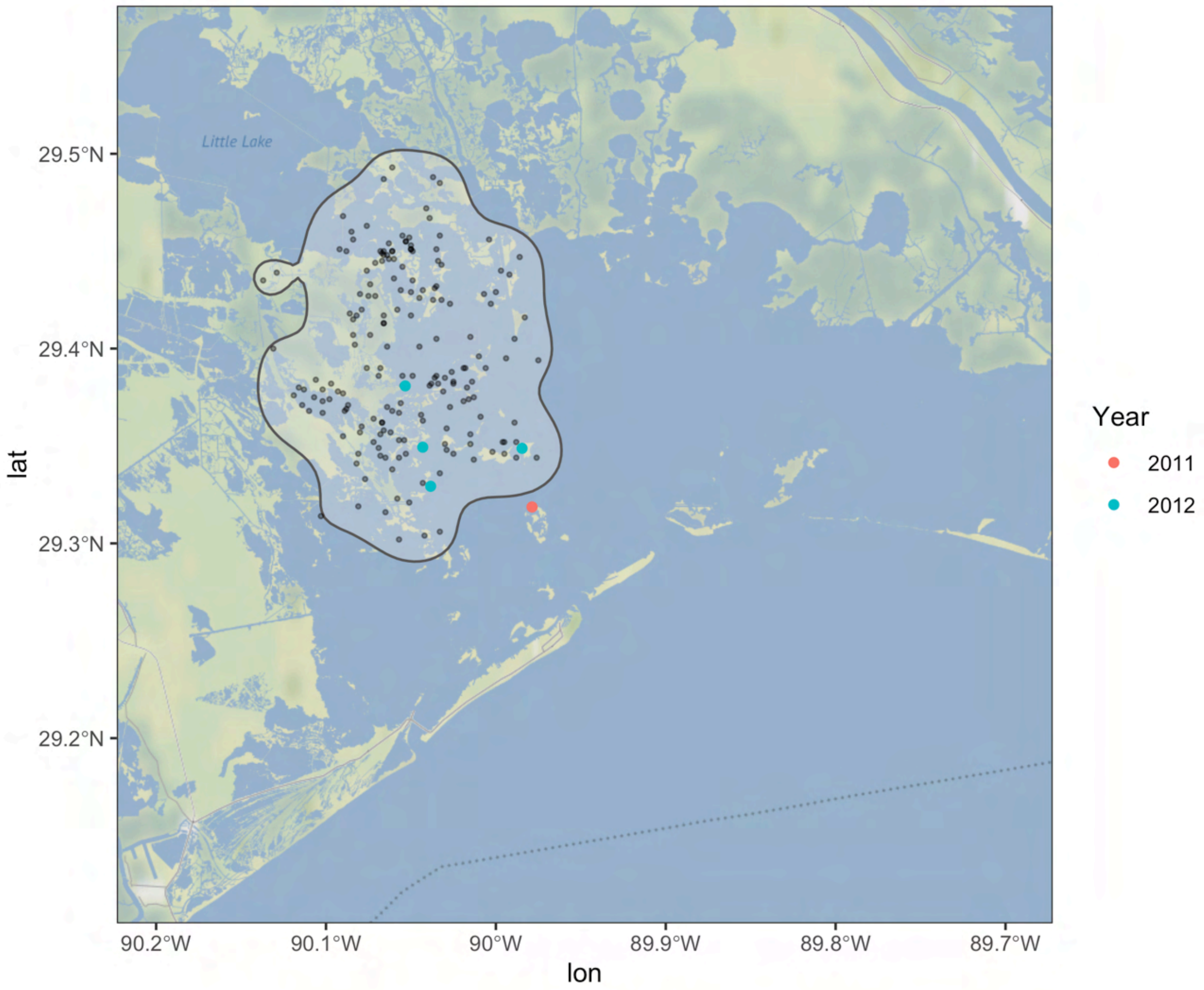

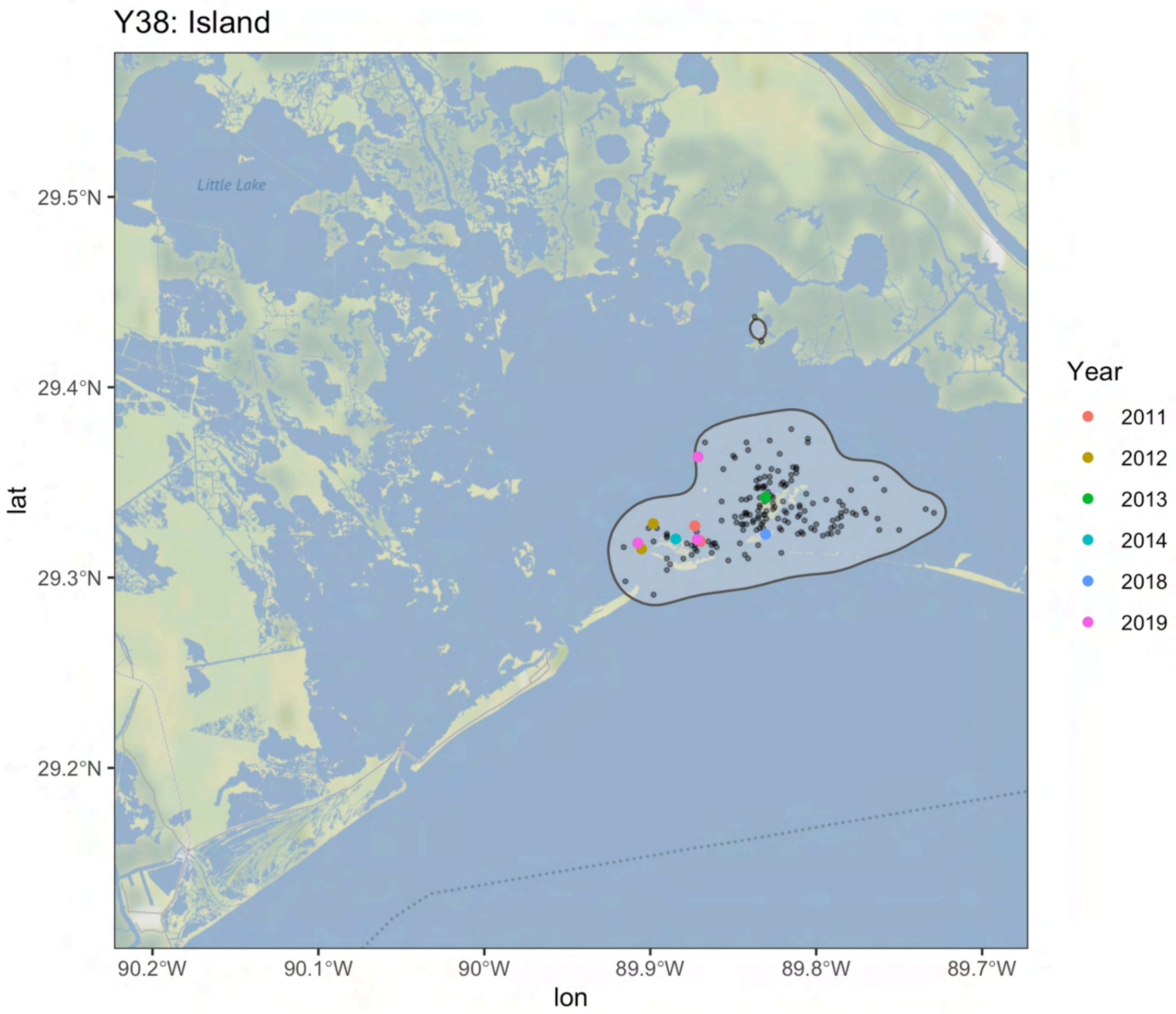
Y38: Island
Little Lake
29.5°N
29.4°N
29.3°N
29.2°N
lat
90.2°W
90.1°W
90°W
89.9°W
89.8°W
89.7°W
lon
Year
2011
2012
2013
2014
2018
2019

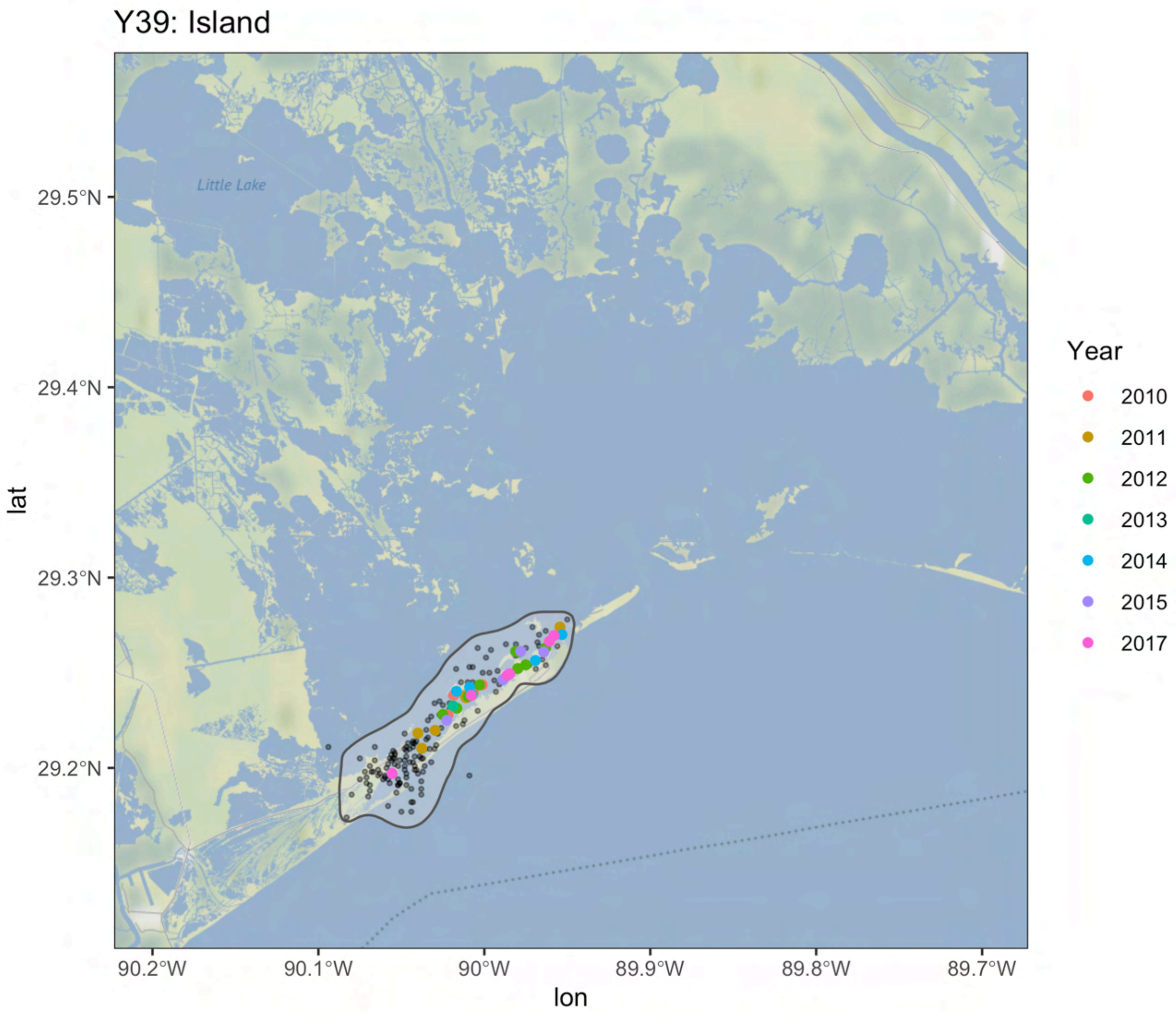
Y39: Island
Little Lake
29.5°N
29.4°N
29.3°N
29.2°N
lat
90.2°W
90.1°W
90°W
89.9°W
89.8°W
89.7°W
lon
Year
2010
2011
2012
2013
2014
2015
2017

Y40: Island

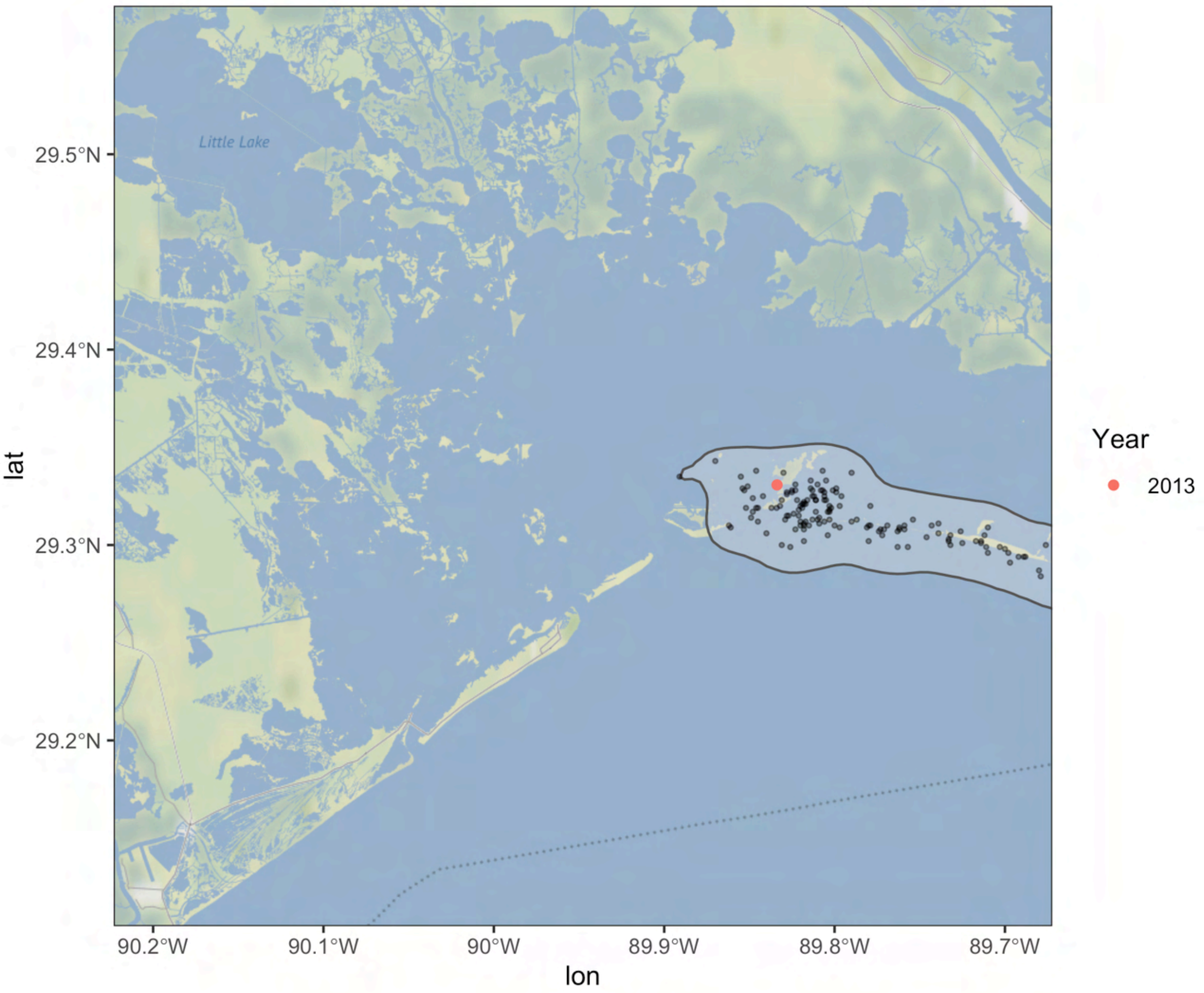

Little Lake
29.5°N
29.4°N
29.3°N
29.2°N
lat
90.2°W
90.1°W
90°W
89.9°W
89.8°W
89.7°W
lon
Year
2013

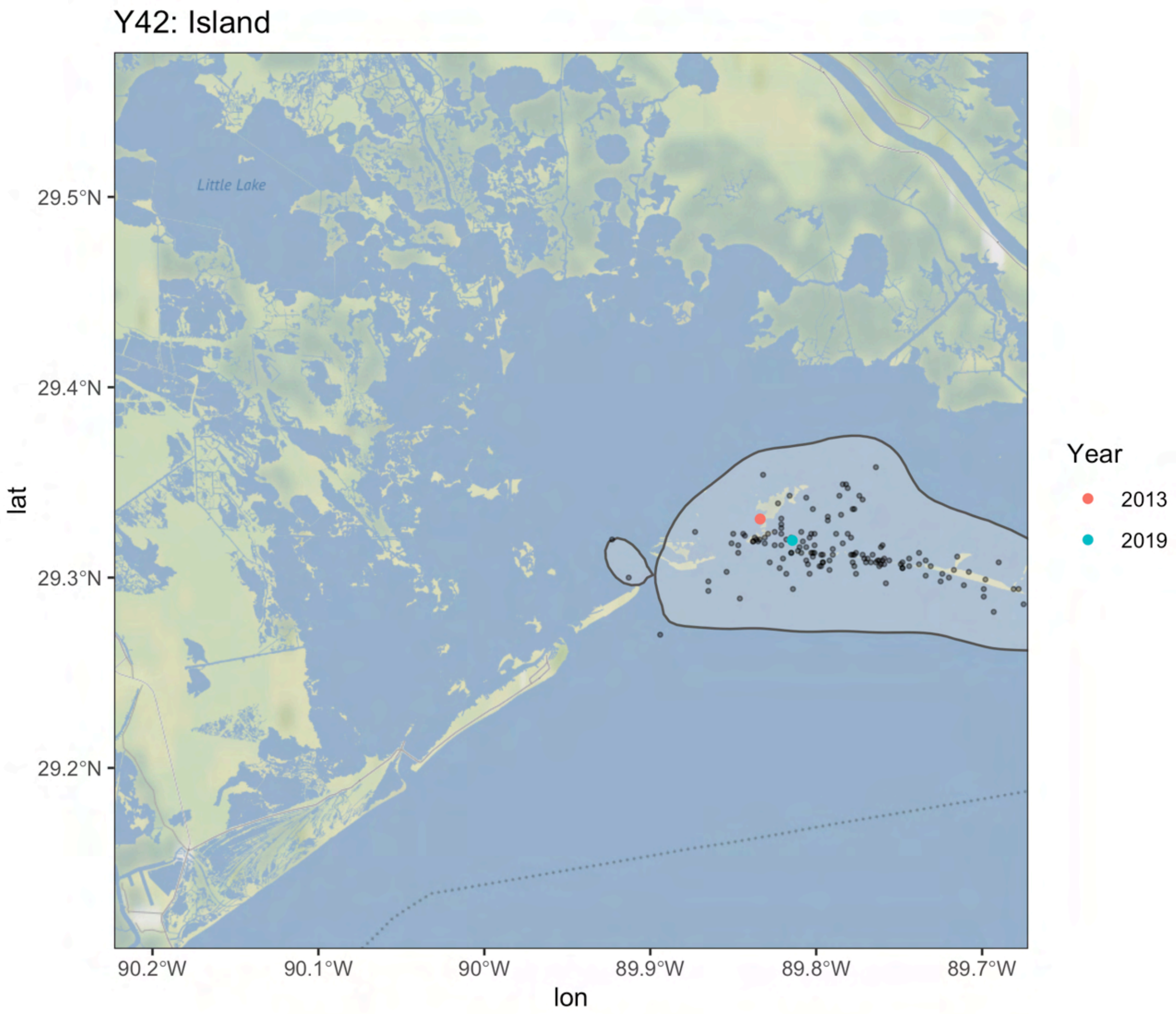
Y42: Island
Little Lake
29.5°N
29.4°N
29.3°N
29.2°N
lat
90.2°W
90.1°W
90°W
89.9°W
89.8°W
89.7°W
lon
Year
2013
2019

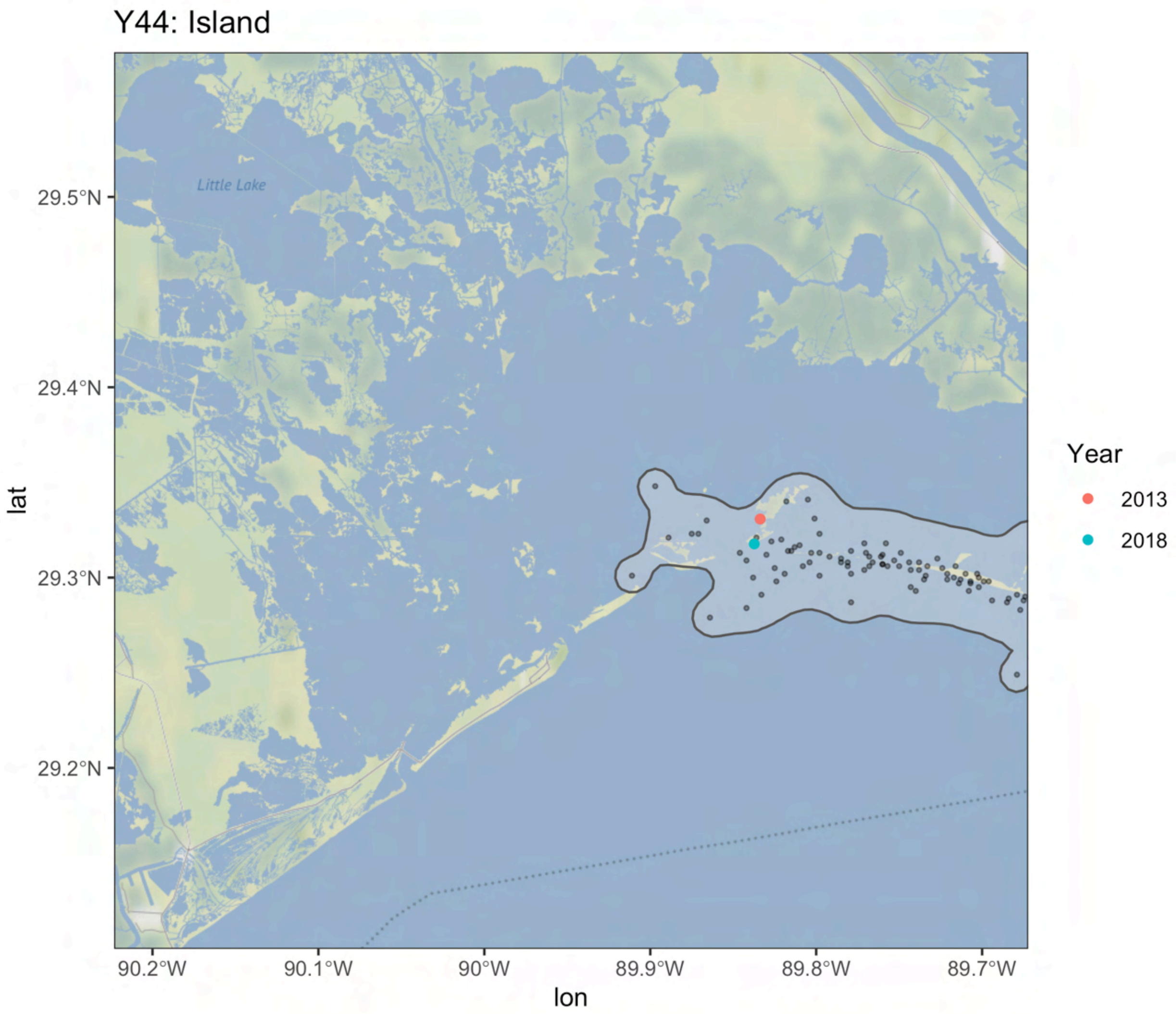
Y44: Island
Little Lake
29.5°N
29.4°N
29.3°N
29.2°N
lat
90.2°W
90.1°W
90°W
89.9°W
89.8°W
89.7°W
lon
Year
2013
2018

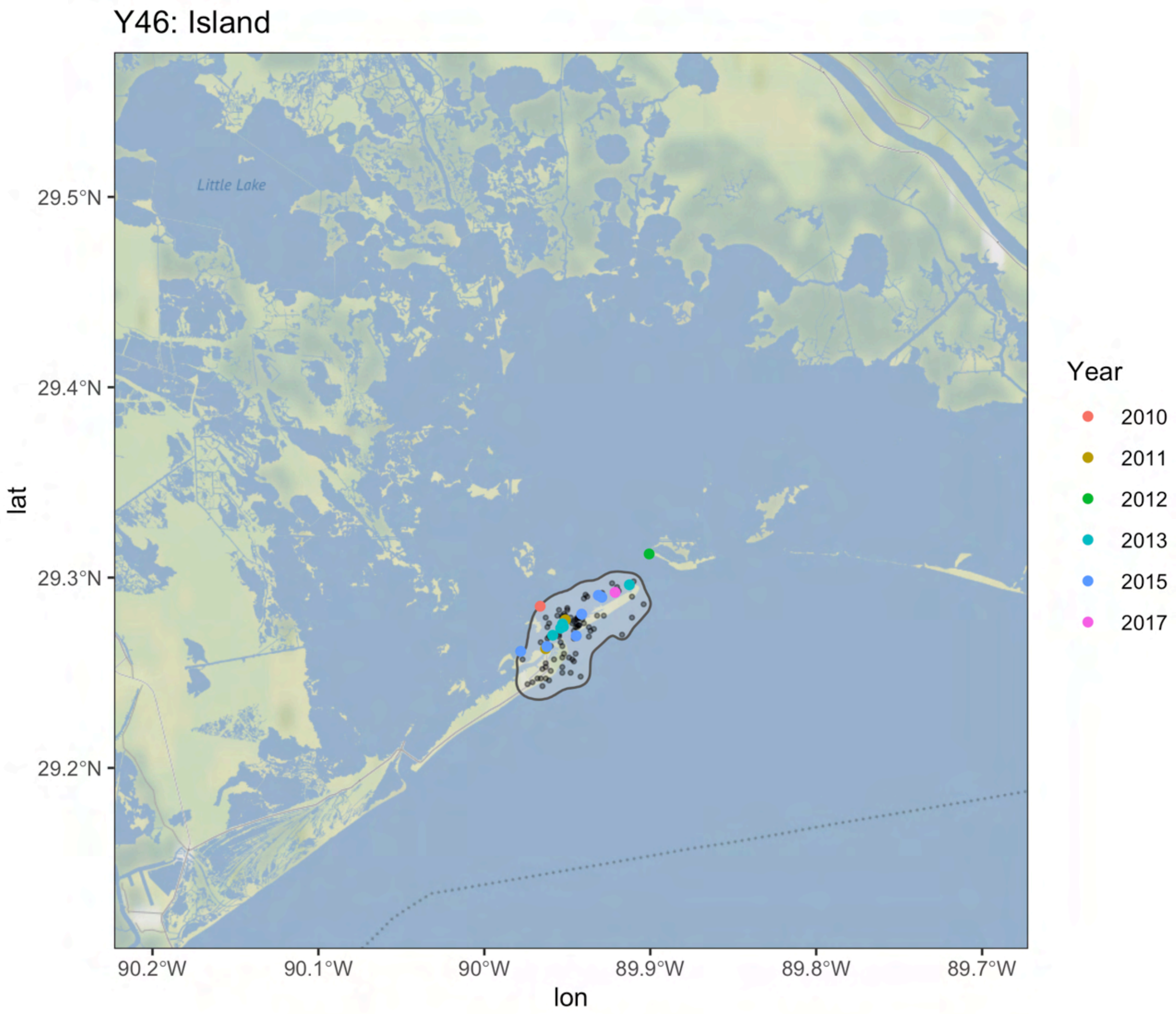
Y46: Island
Little Lake
29.5°N
29.4°N
29.3°N
29.2°N
lat
90.2°W
90.1°W
90°W
89.9°W
89.8°W
89.7°W
lon
Year
2010
2011
2012
2013
2015
2017

Y65: Island

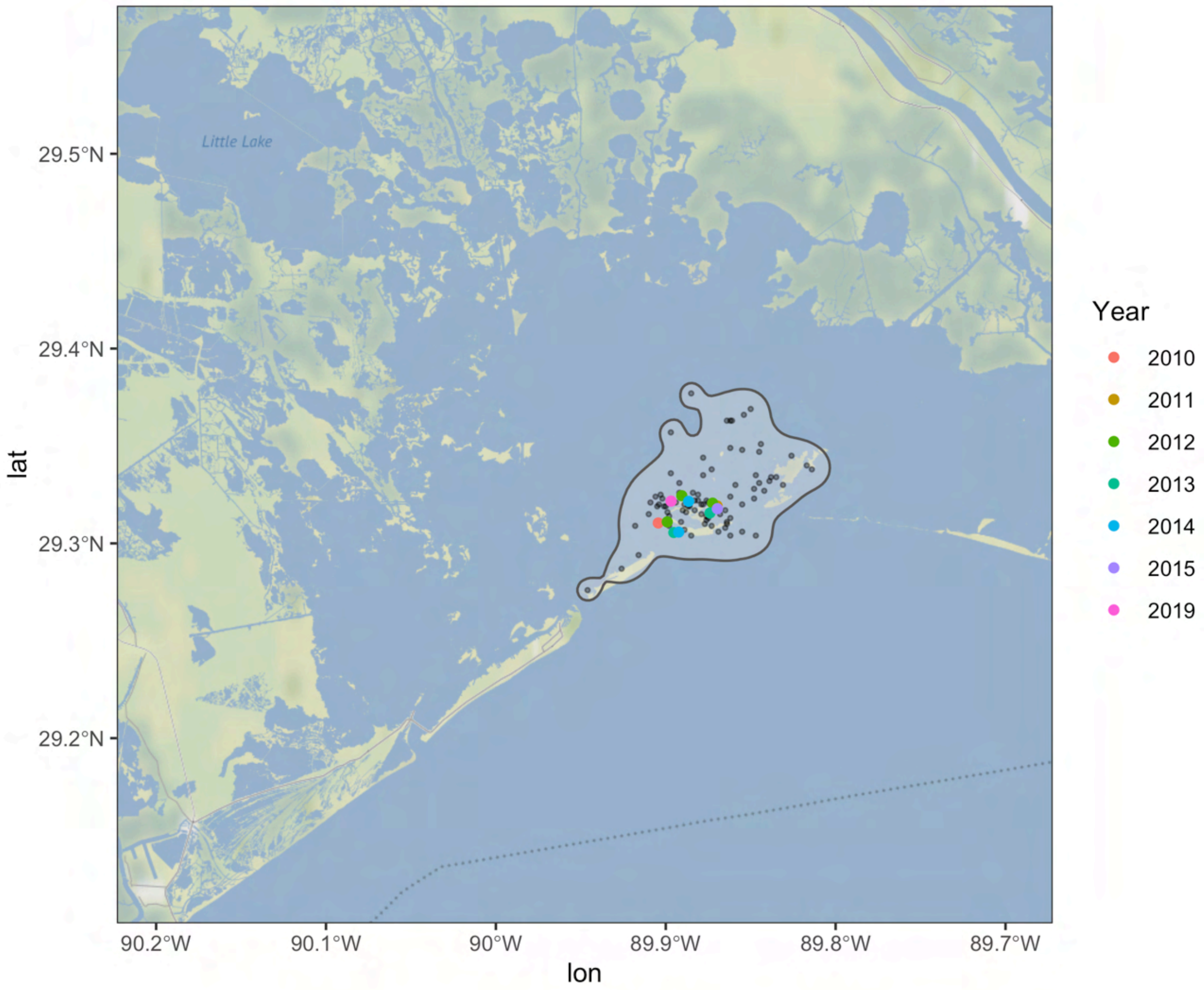

Little Lake
29.5°N
29.4°N
29.3°N
29.2°N
lat
90.2°W
90.1°W
90°W
89.9°W
89.8°W
89.7°W
lon
Year
2010
2011
2012
2013
2014
2015
2019

Y67: Island

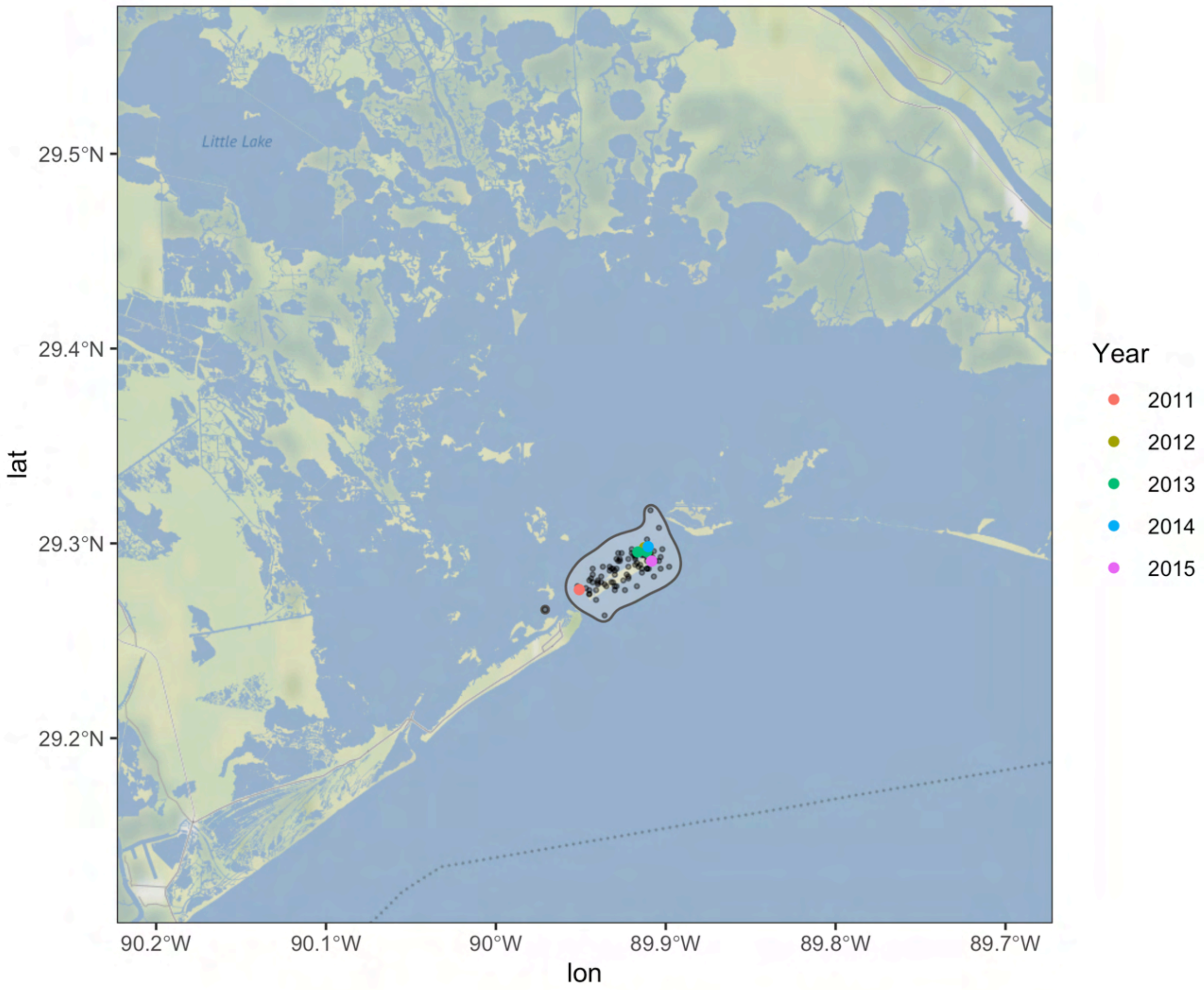
Little Lake
29.5°N
29.4°N
29.3°N
29.2°N
lat
90.2°W
90.1°W
90°W
89.9°W
89.8°W
89.7°W
lon
Year
2011
2012
2013
2014
2015

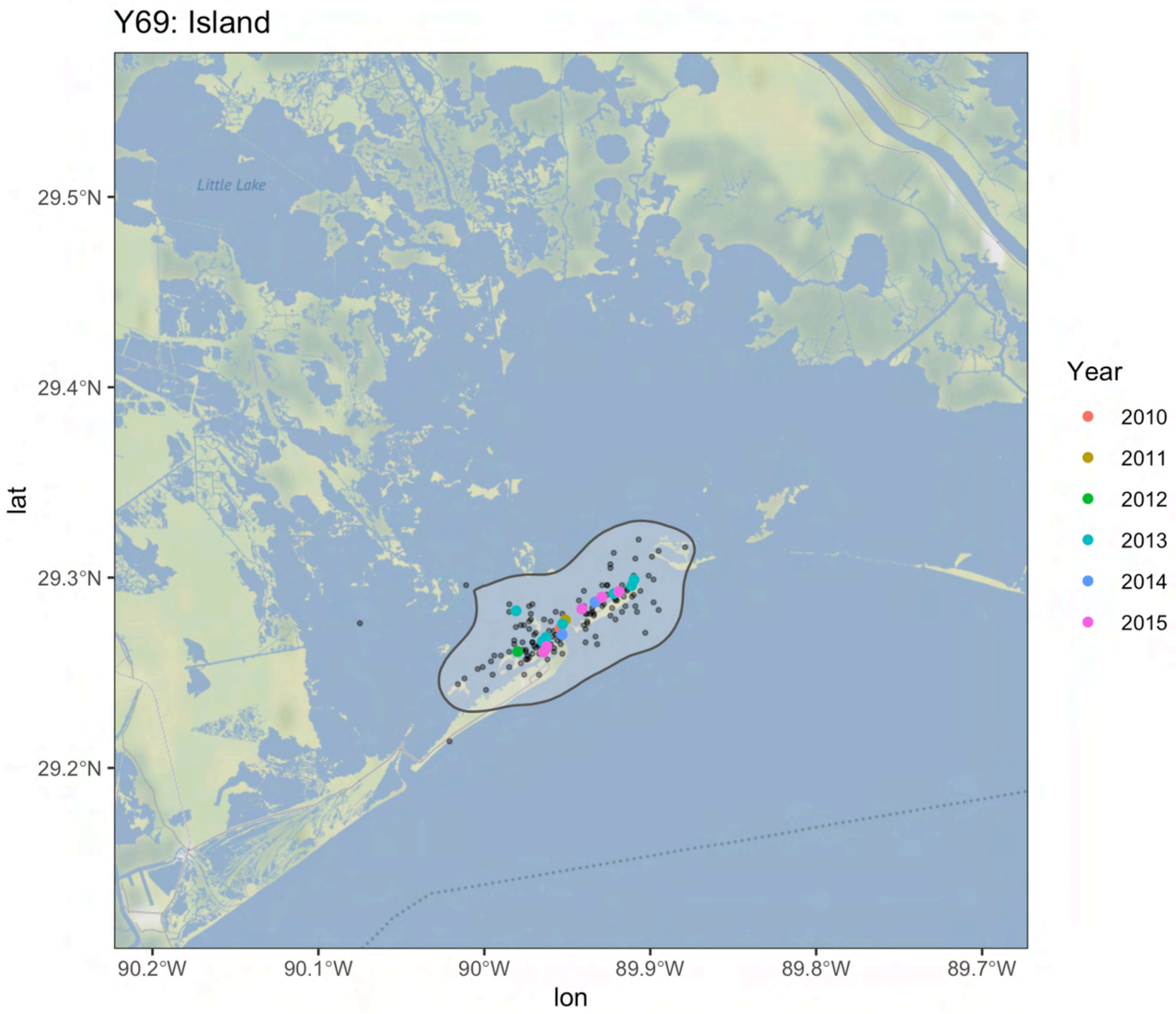

Y69: Island
Little Lake
29.5°N
29.4°N
29.3°N
29.2°N
lat
90.2°W
90.1°W
90°W
89.9°W
89.8°W
89.7°W
lon
Year
2010
2011
2012
2013
2014
2015

Y71: Island

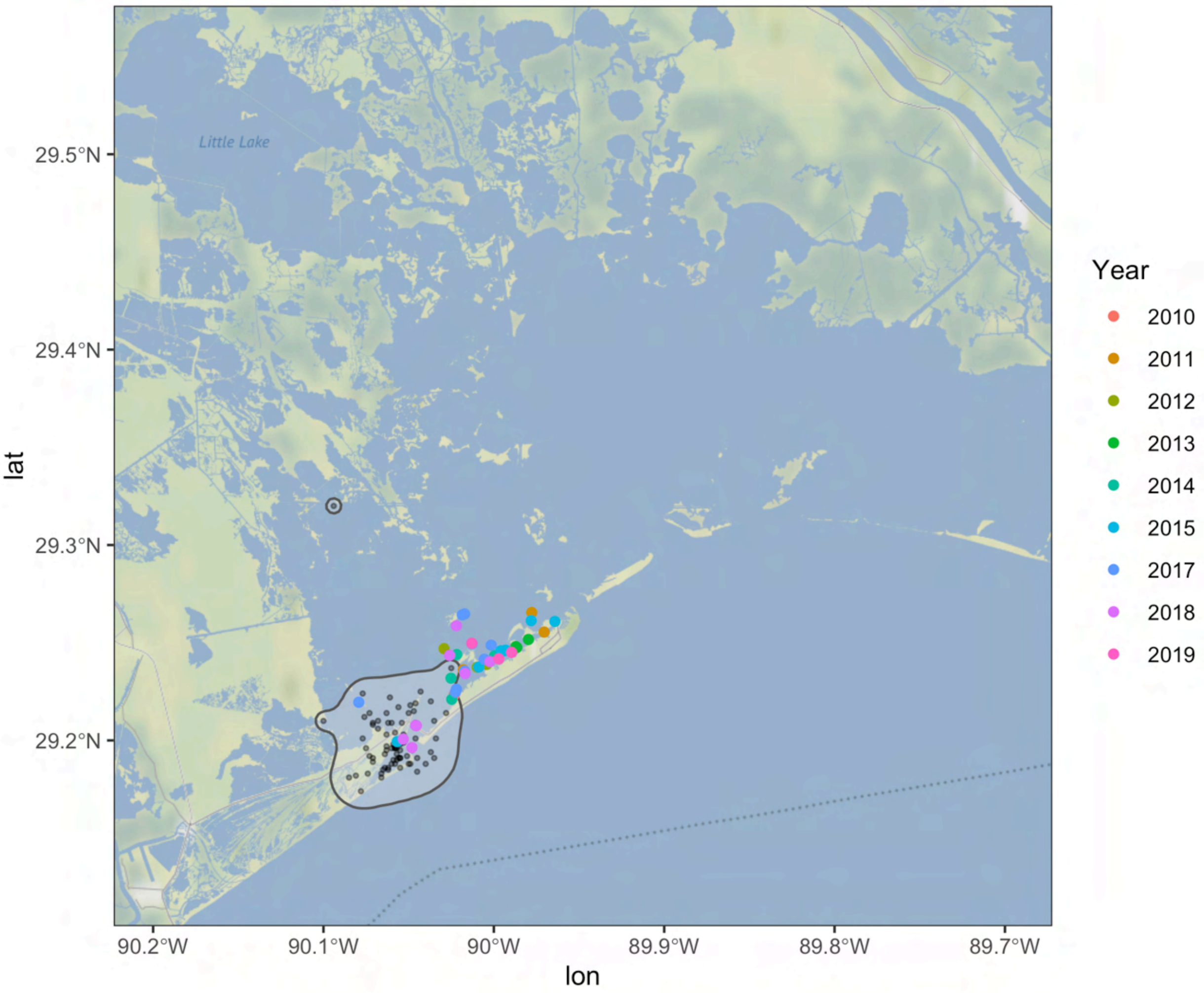

Little Lake
29.5°N
29.4°N
29.3°N
29.2°N
lat
90.2°W
90.1°W
90°W
89.9°W
89.8°W
89.7°W
lon
Year
2010
2011
2012
2013
2014
2015
2017
2018
2019

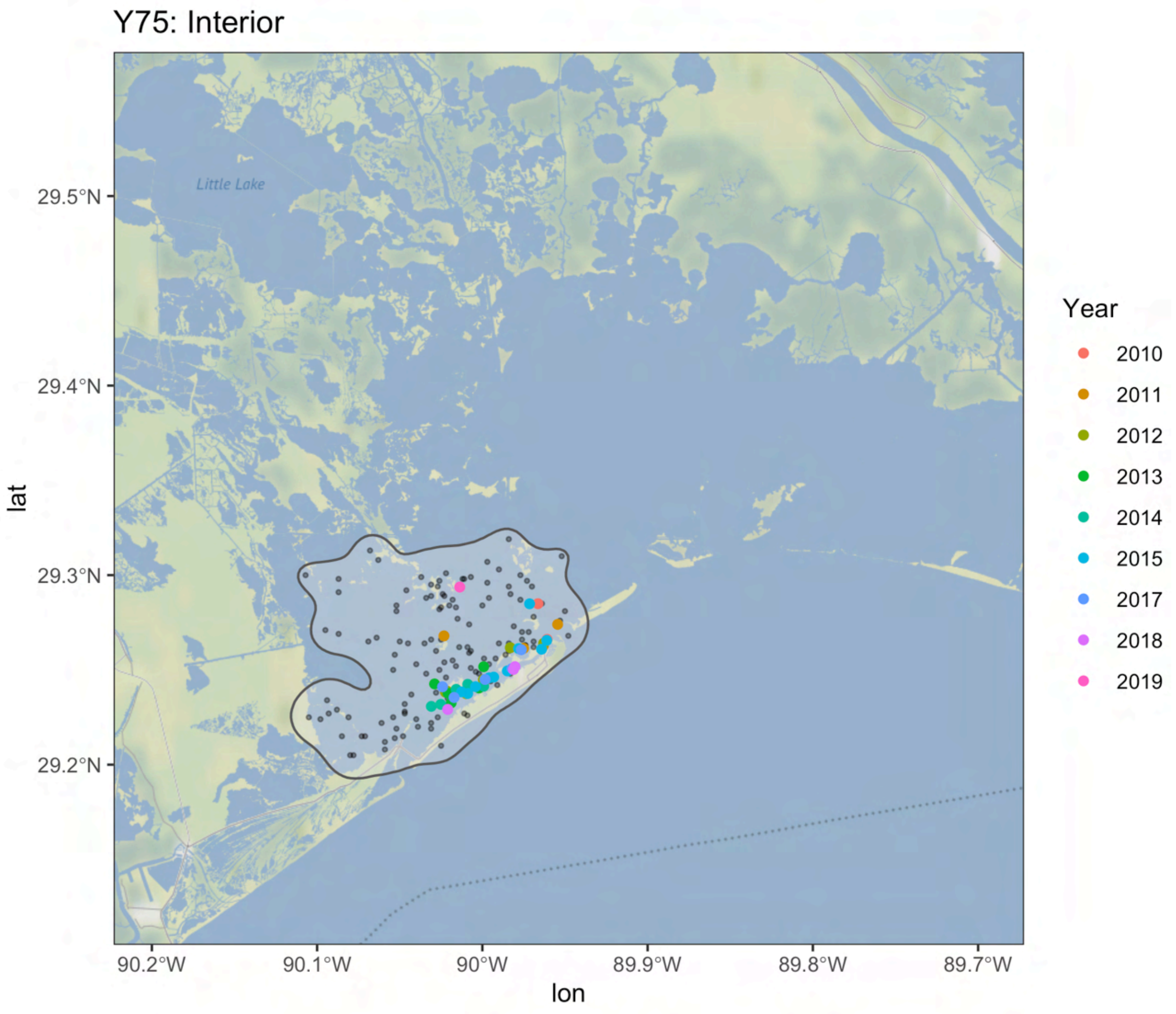
Y75: Interior
Little Lake
29.5°N
29.4°N
29.3°N
29.2°N
lat
90.2°W
90.1°W
90°W
89.9°W
89.8°W
89.7°W
lon
Year
2010
2011
2012
2013
2014
2015
2017
2018
2019

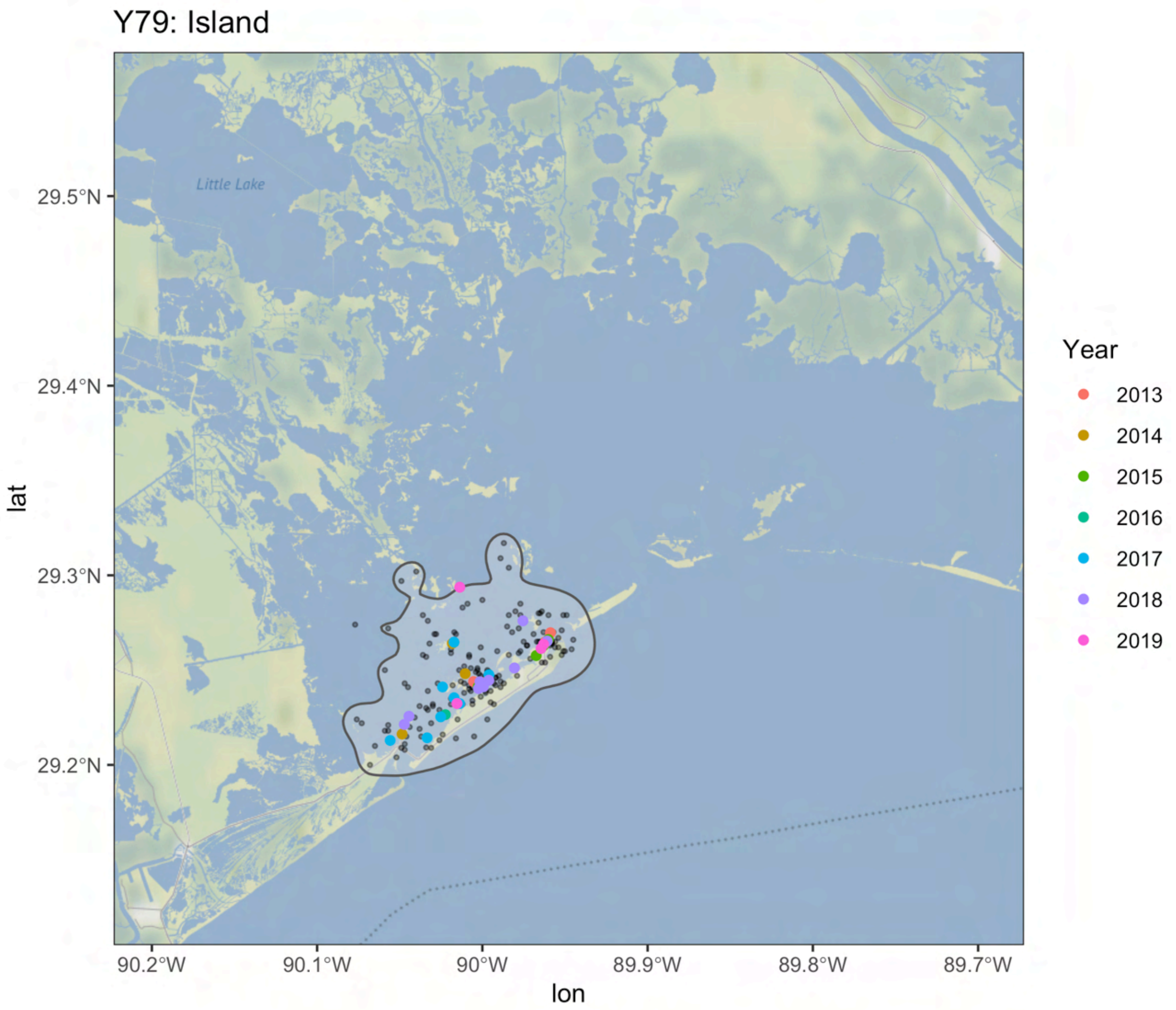
Y79: Island
Little Lake
29.5°N
29.4°N
29.3°N
29.2°N
lat
90.2°W
90.1°W
90°W
89.9°W
89.8°W
89.7°W
lon
Year
2013
2014
2015
2016
2017
2018
2019

Y80: Island

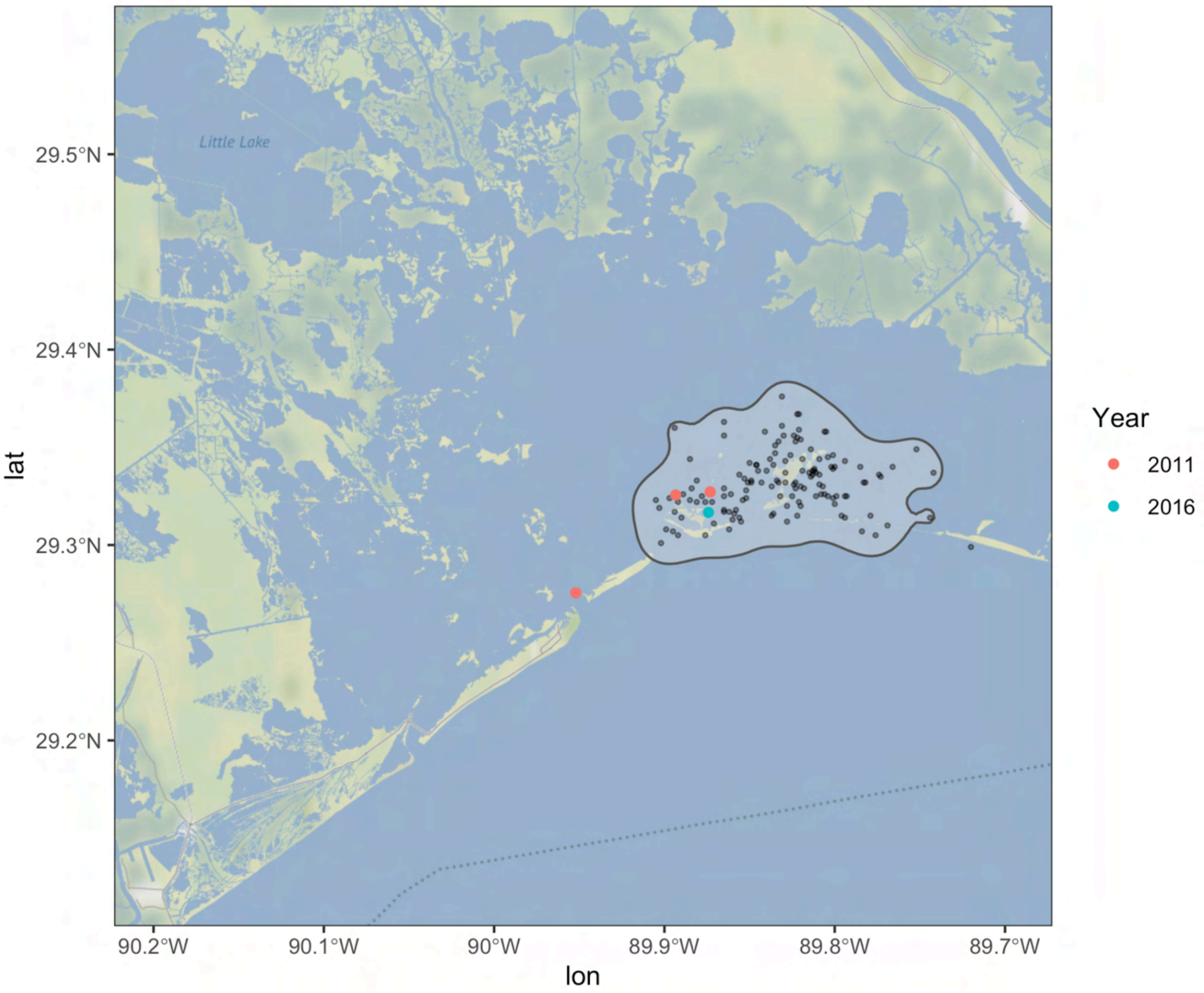
Little Lake
29.5°N
29.4°N
29.3°N
29.2°N
lat
90.2°W
90.1°W
90°W
89.9°W
89.8°W
89.7°W
lon
Year
2011
2016

Y81: Island

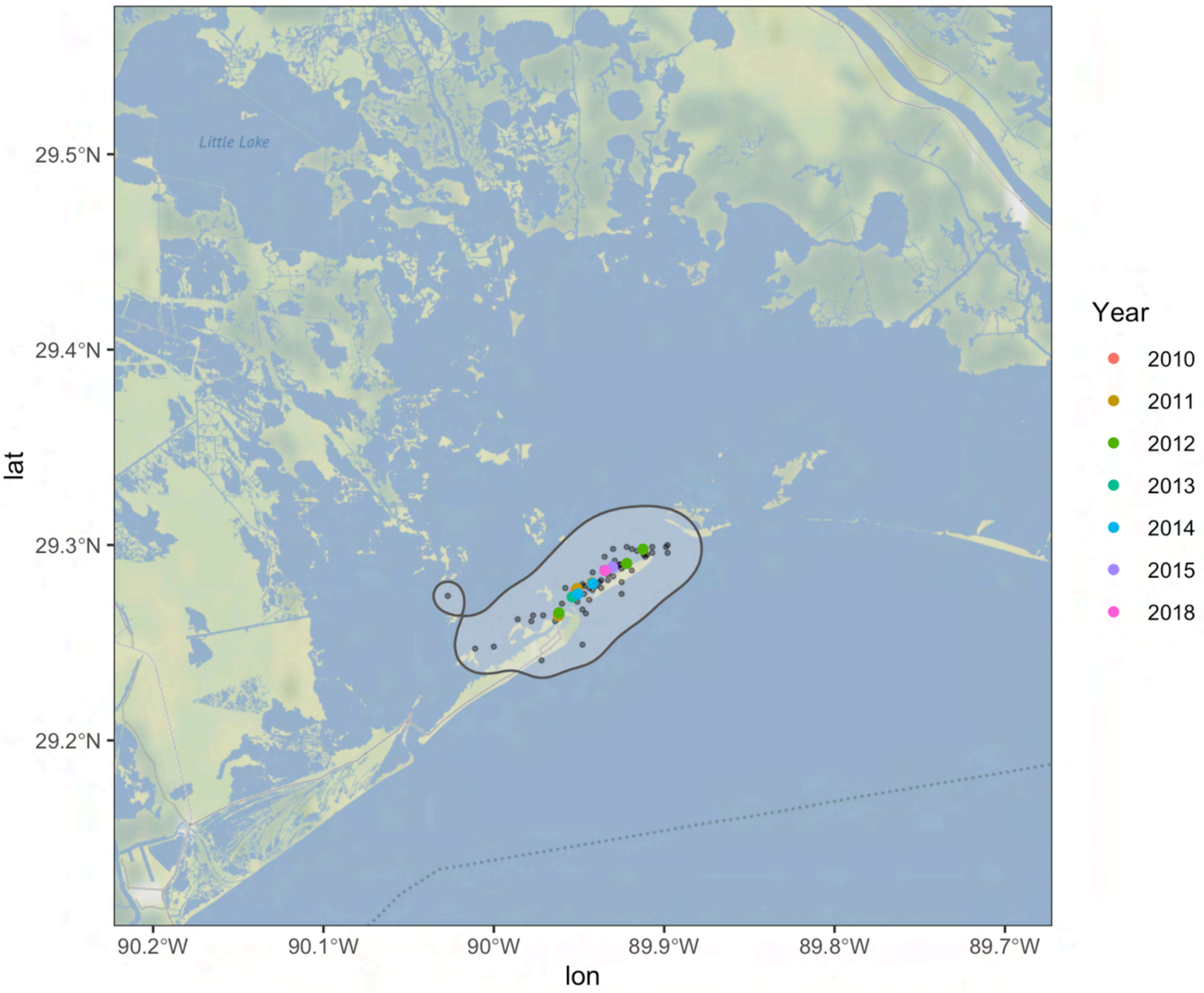

Little Lake
29.5°N
29.4°N
29.3°N
29.2°N
lat
90.2°W
90.1°W
90°W
89.9°W
89.8°W
89.7°W
lon
Year
2010
2011
2012
2013
2014
2015
2018

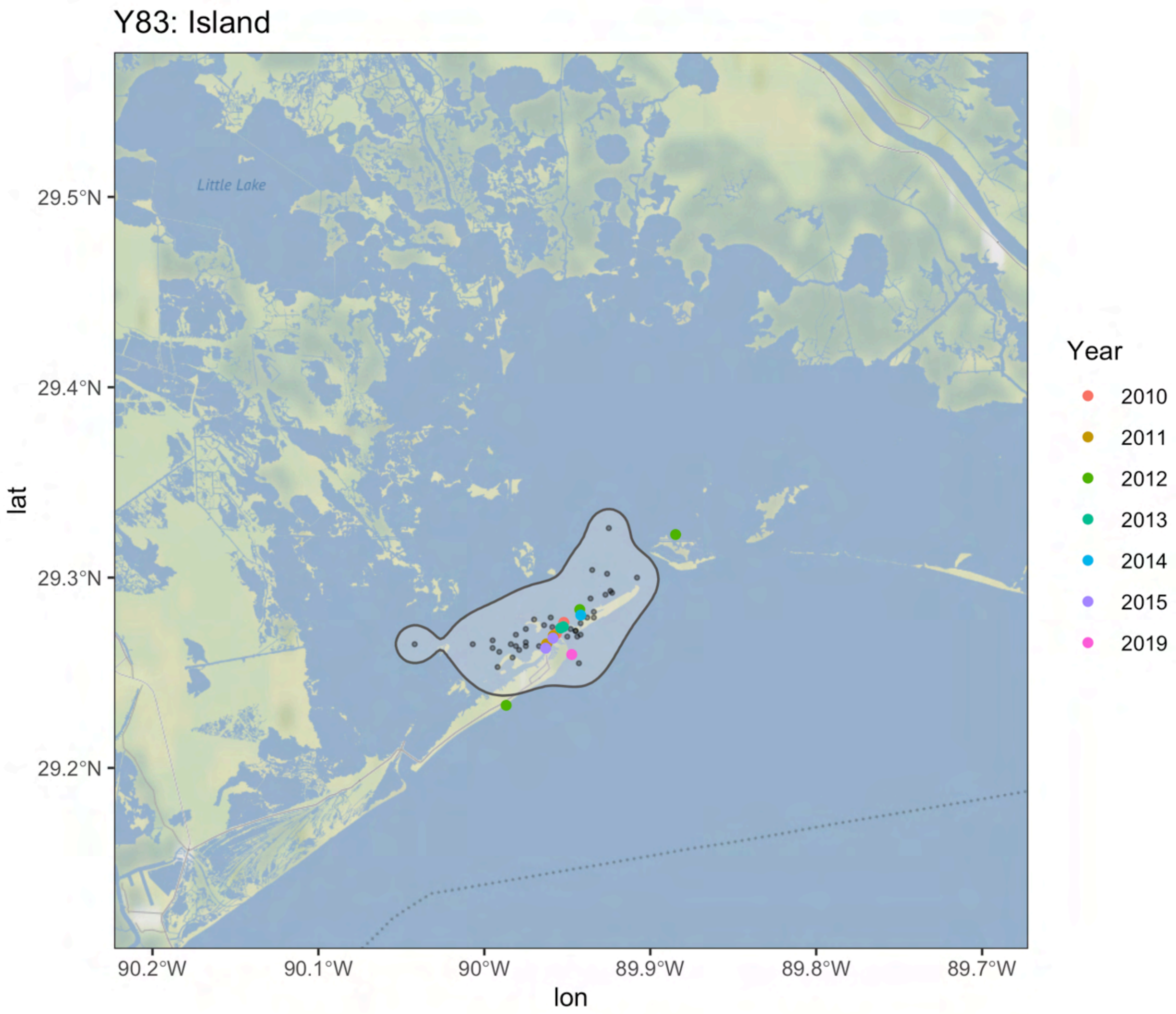

Y83: Island
Little Lake
29.5°N
29.4°N
29.3°N
29.2°N
lat
90.2°W
90.1°W
90°W
89.9°W
89.8°W
89.7°W
lon
Year
2010
2011
2012
2013
2014
2015
2019

Y85: Island

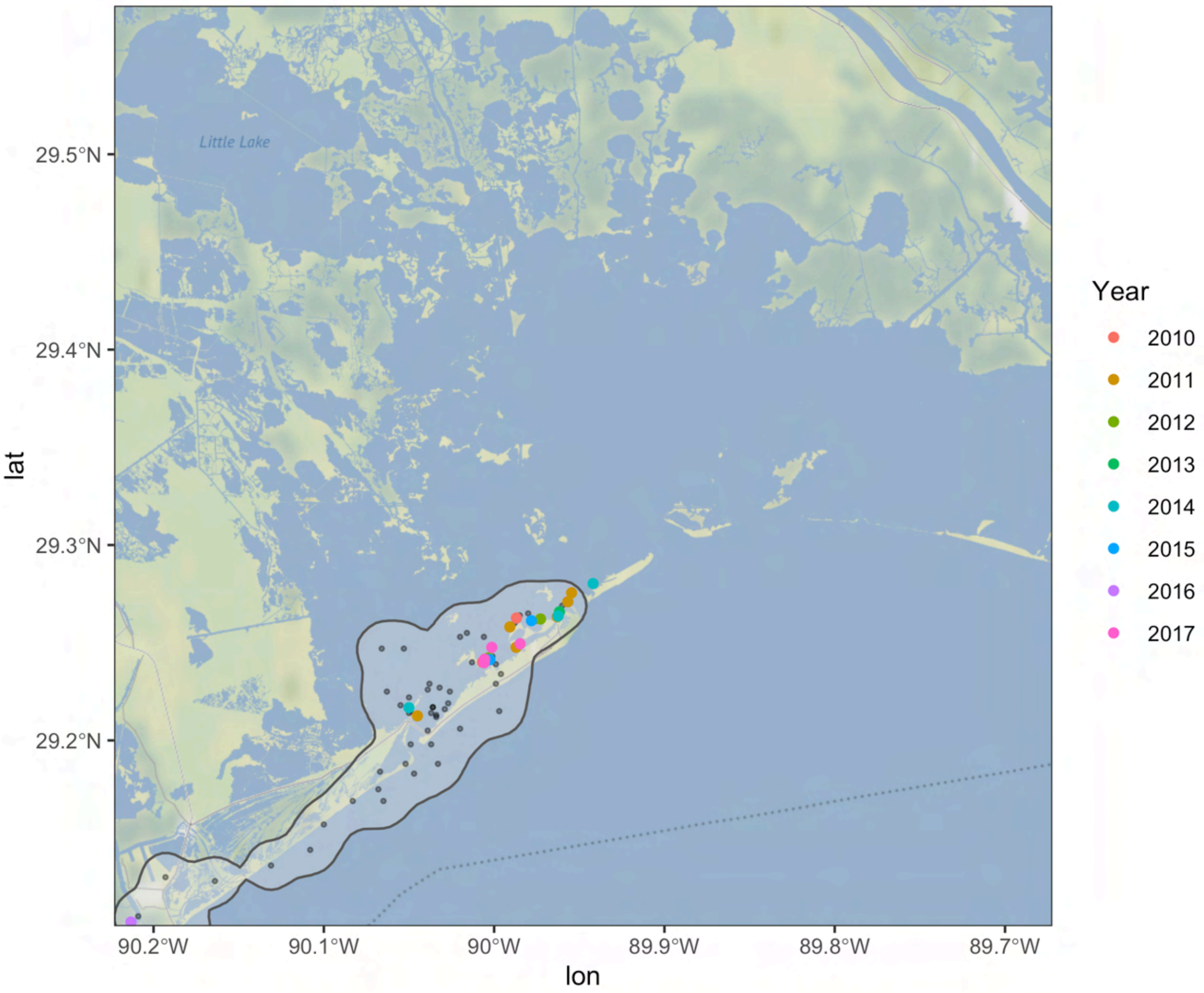
Little Lake
29.5°N
29.4°N
29.3°N
29.2°N
lat
90.2°W
90.1°W
90°W
89.9°W
89.8°W
89.7°W
lon
Year
2010
2011
2012
2013
2014
2015
2016
2017

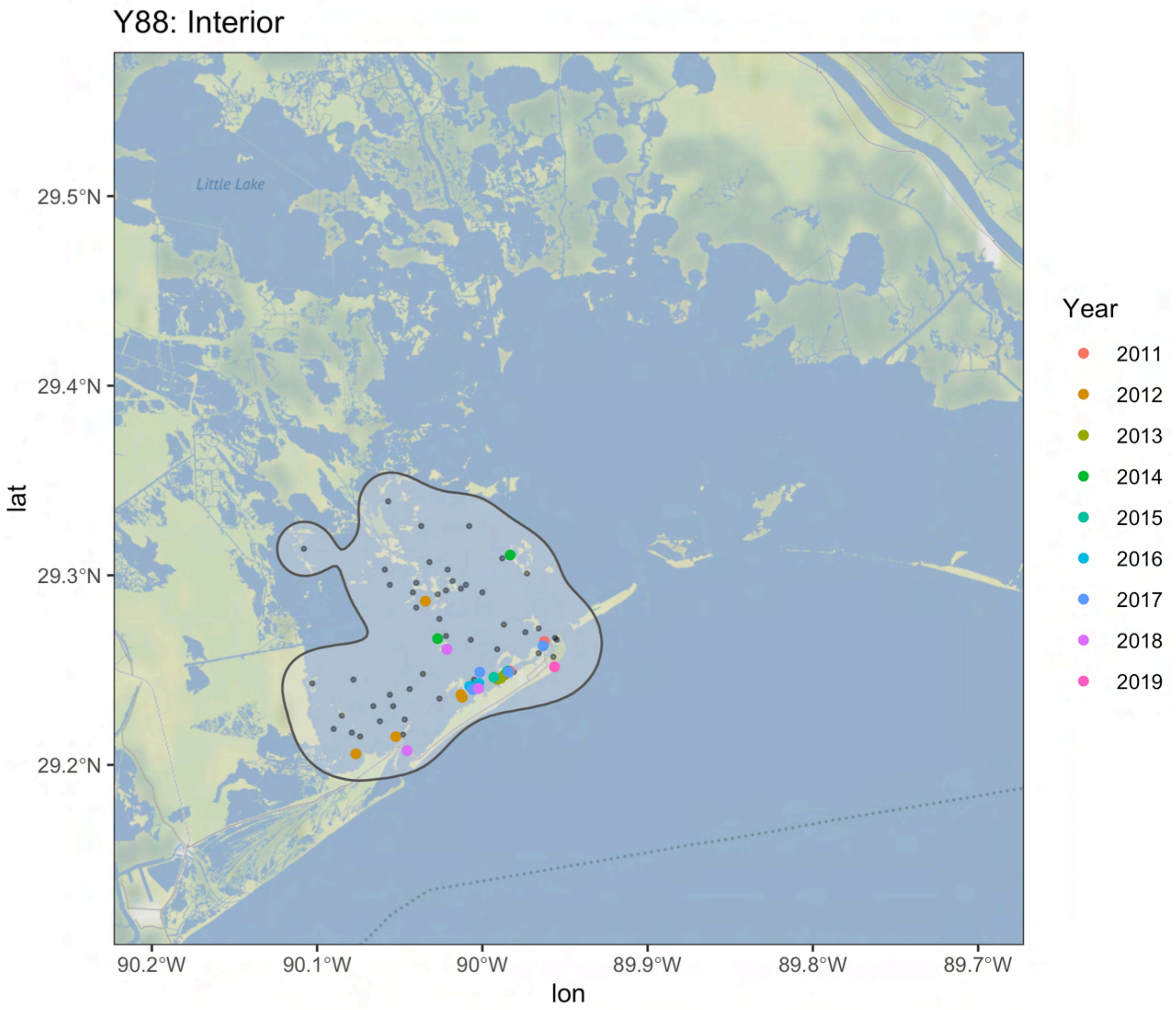

Y88: Interior
Little Lake
29.5°N
29.4°N
29.3°N
29.2°N
lat
90.2°W
90.1°W
90°W
89.9°W
89.8°W
89.7°W
lon
Year
2011
2012
2013
2014
2015
2016
2017
2018
2019

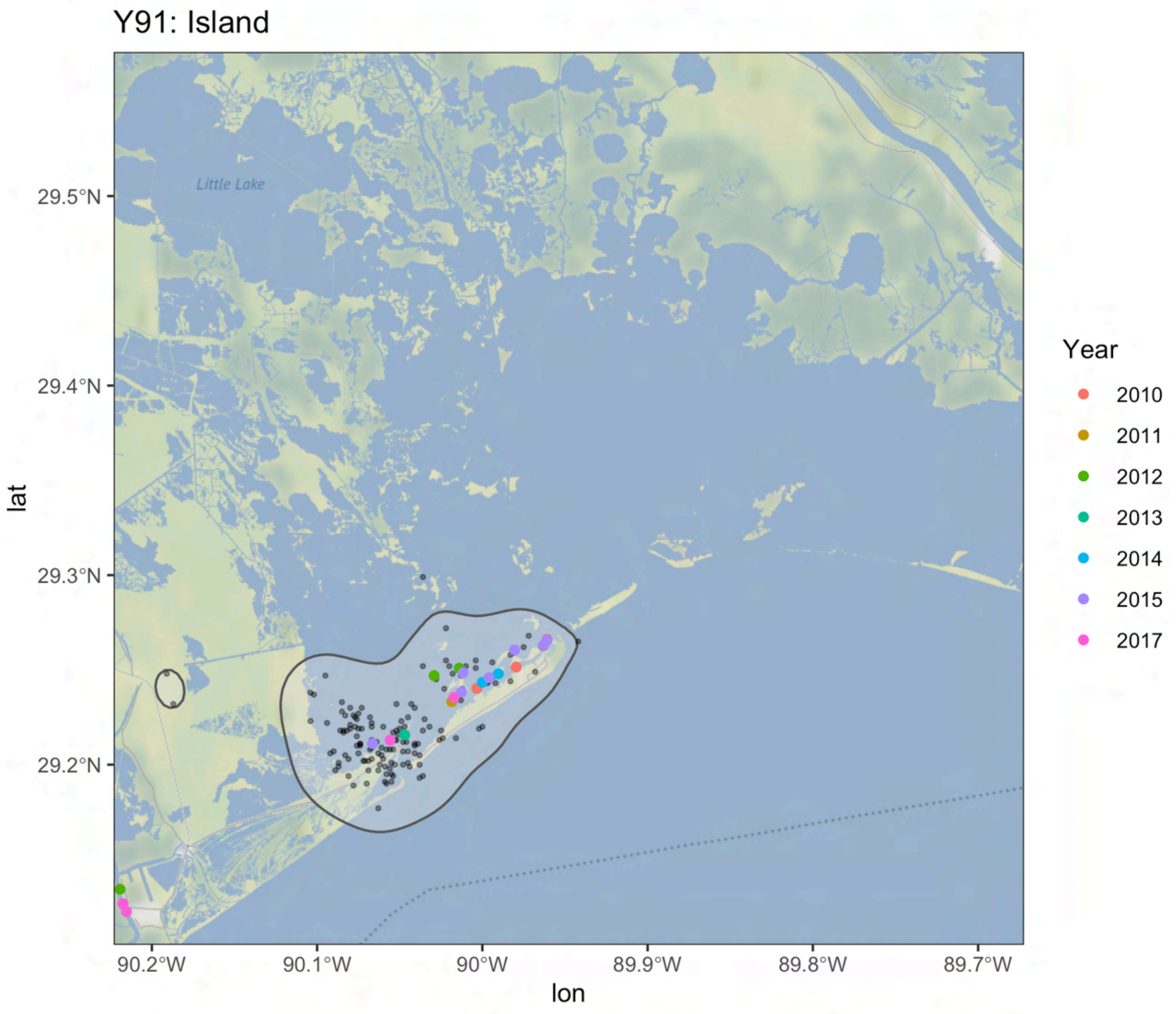

Y91: Island
Little Lake
29.5°N
29.4°N
29.3°N
29.2°N
lat
90.2°W
90.1°W
90°W
89.9°W
89.8°W
89.7°W
lon
Year
2010
2011
2012
2013
2014
2015
2017

Y94: Interior

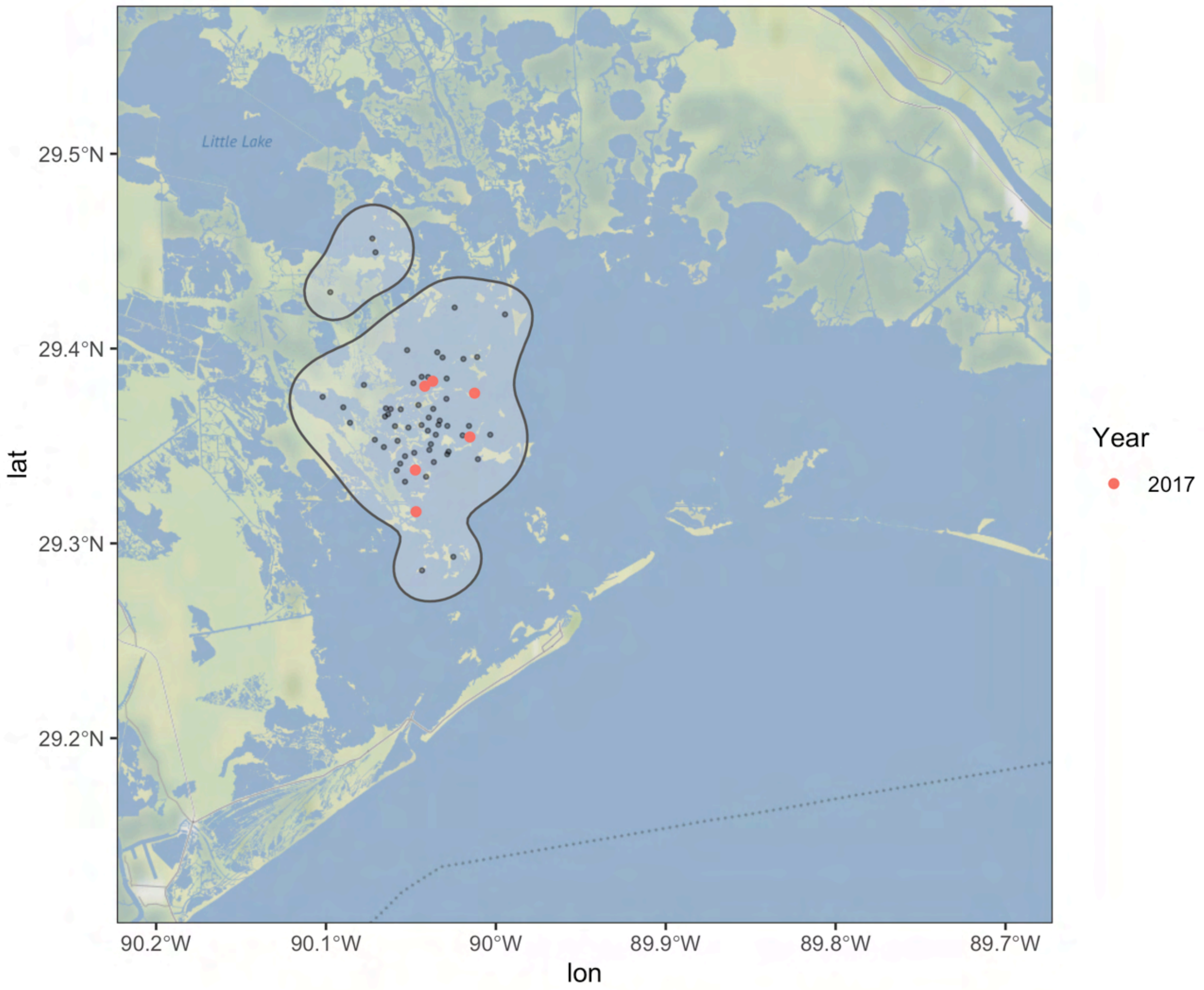

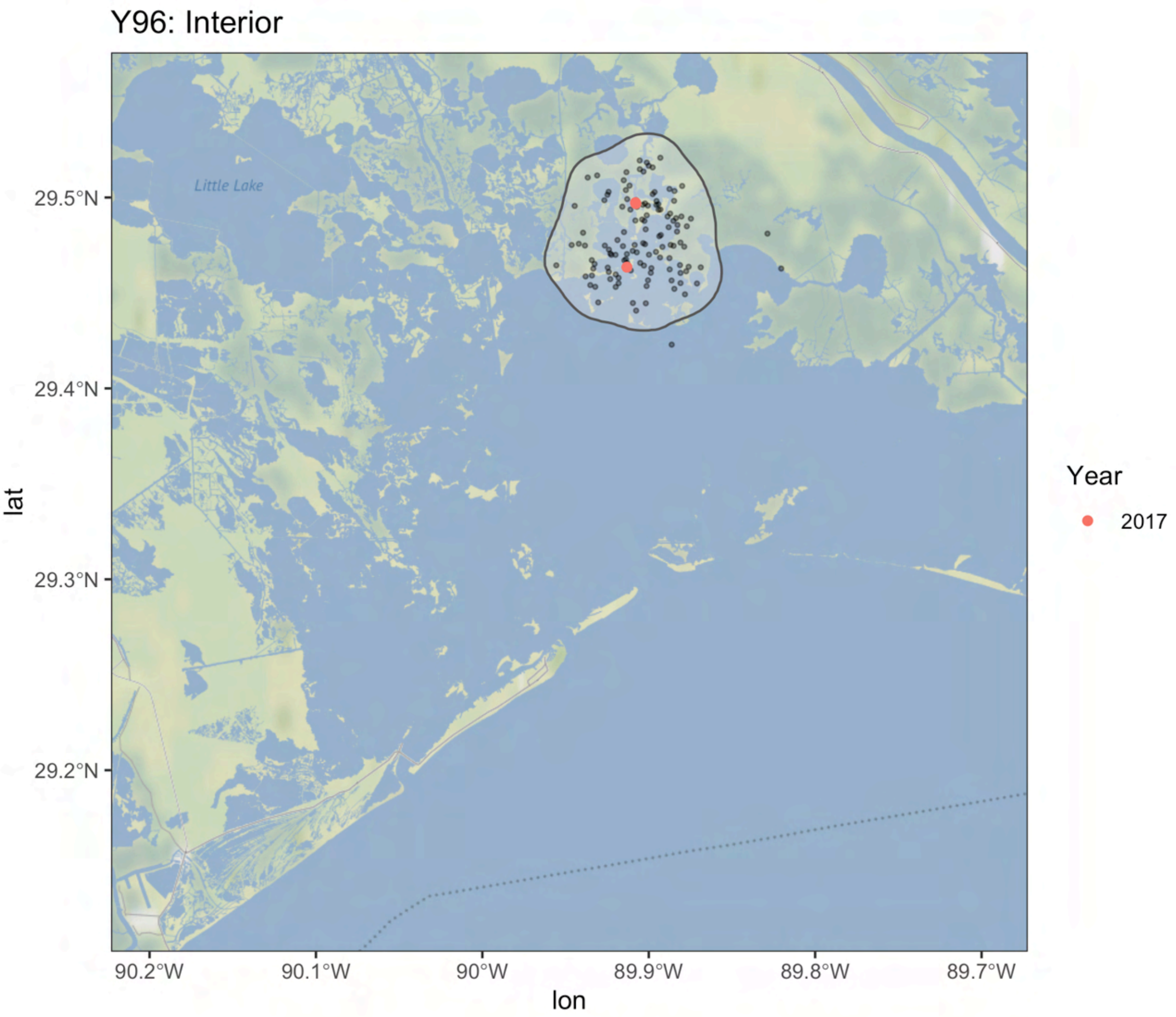

Y96: Interior
Little Lake
29.5°N
29.4°N
29.3°N
29.2°N
lat
90.2°W
90.1°W
90°W
89.9°W
89.8°W
89.7°W
lon
Year
2017

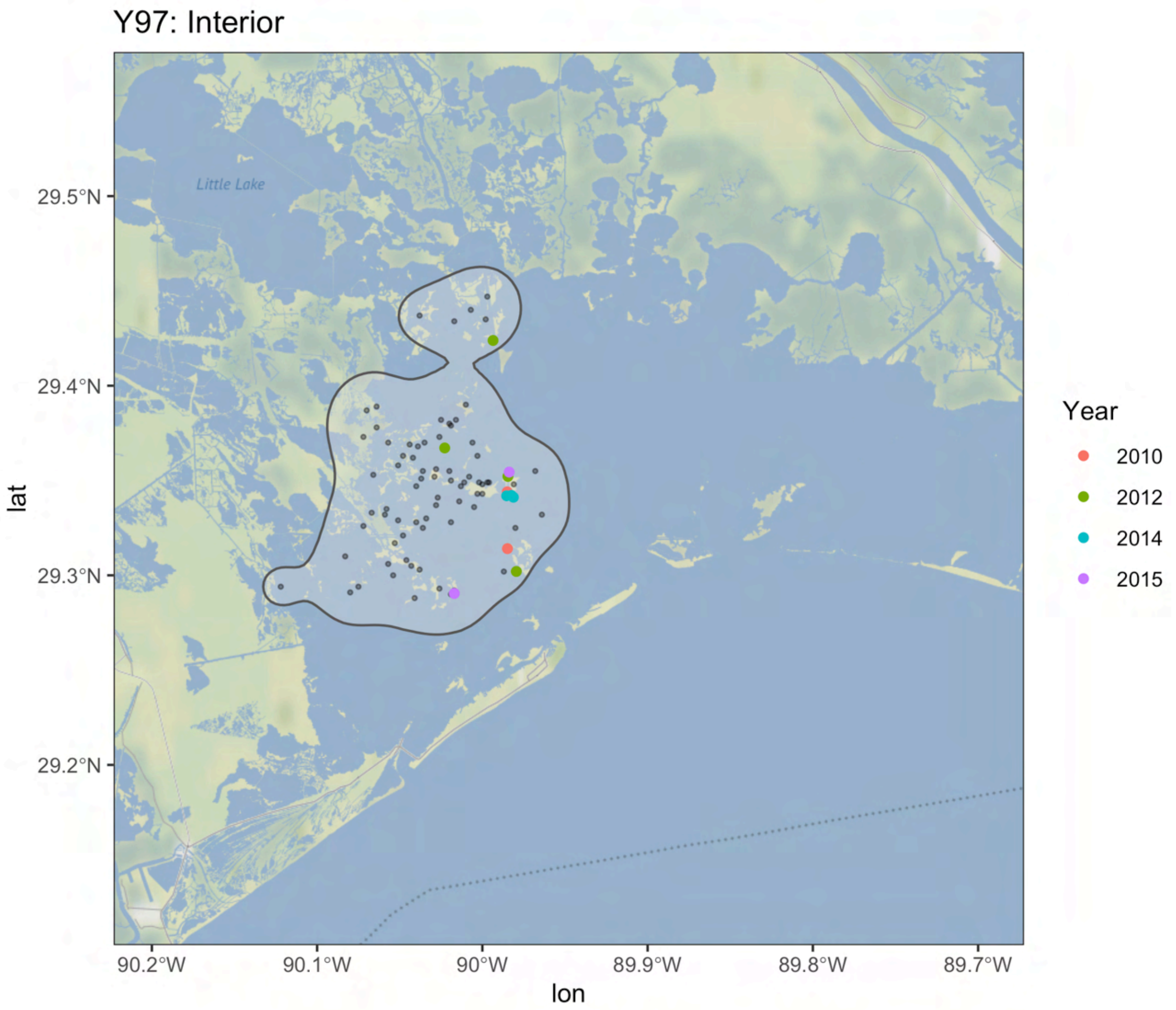
Y97: Interior
Little Lake
29.5°N
29.4°N
29.3°N
29.2°N
lat
90.2°W
90.1°W
90°W
89.9°W
89.8°W
89.7°W
lon
Year
2010
2012
2014
2015

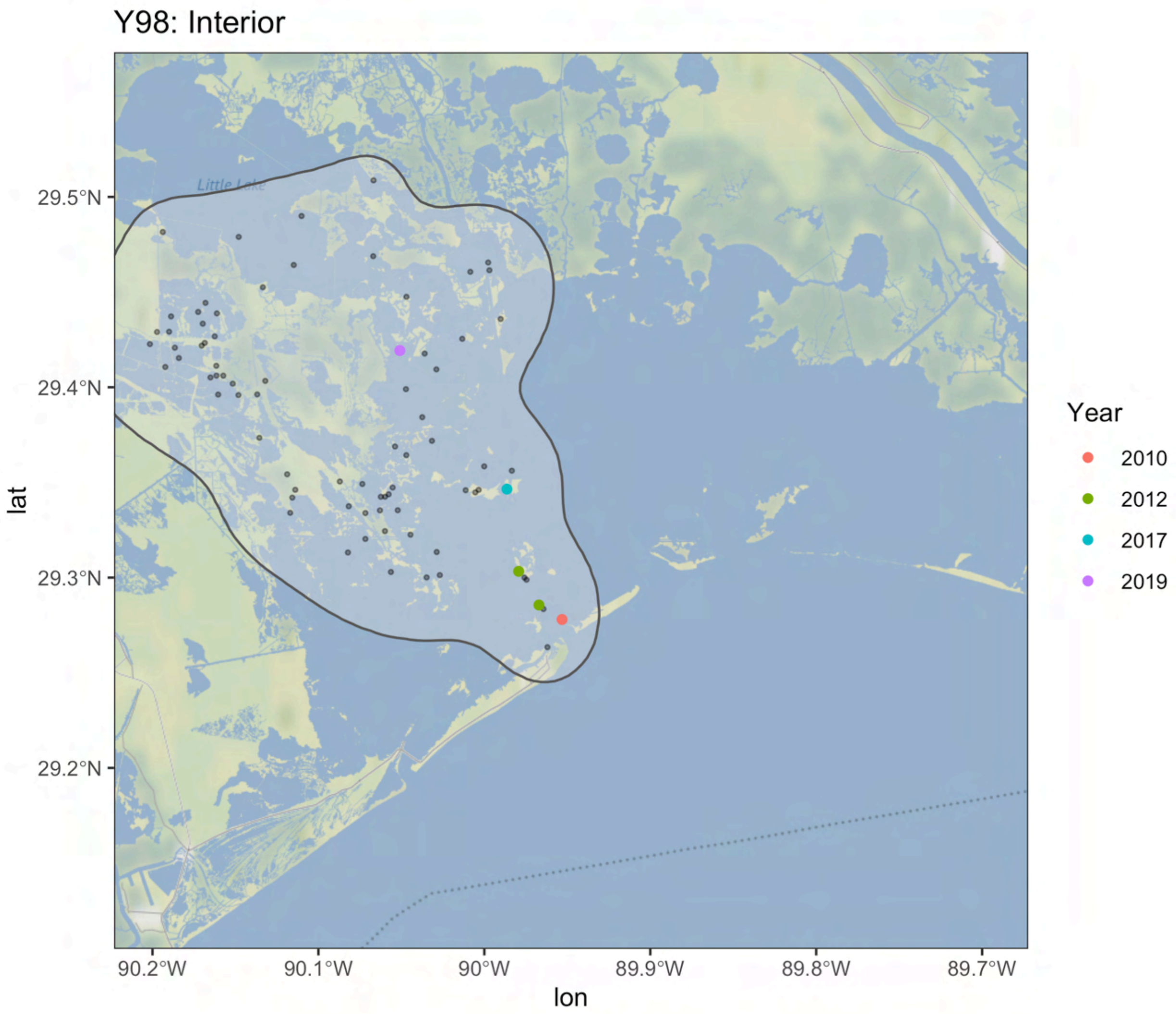

Y98: Interior
Little Lake
29.5°N
29.4°N
29.3°N
29.2°N
lat
90.2°W
90.1°W
90°W
89.9°W
89.8°W
89.7°W
lon
Year
2010
2012
2017
2019

Y99: Island

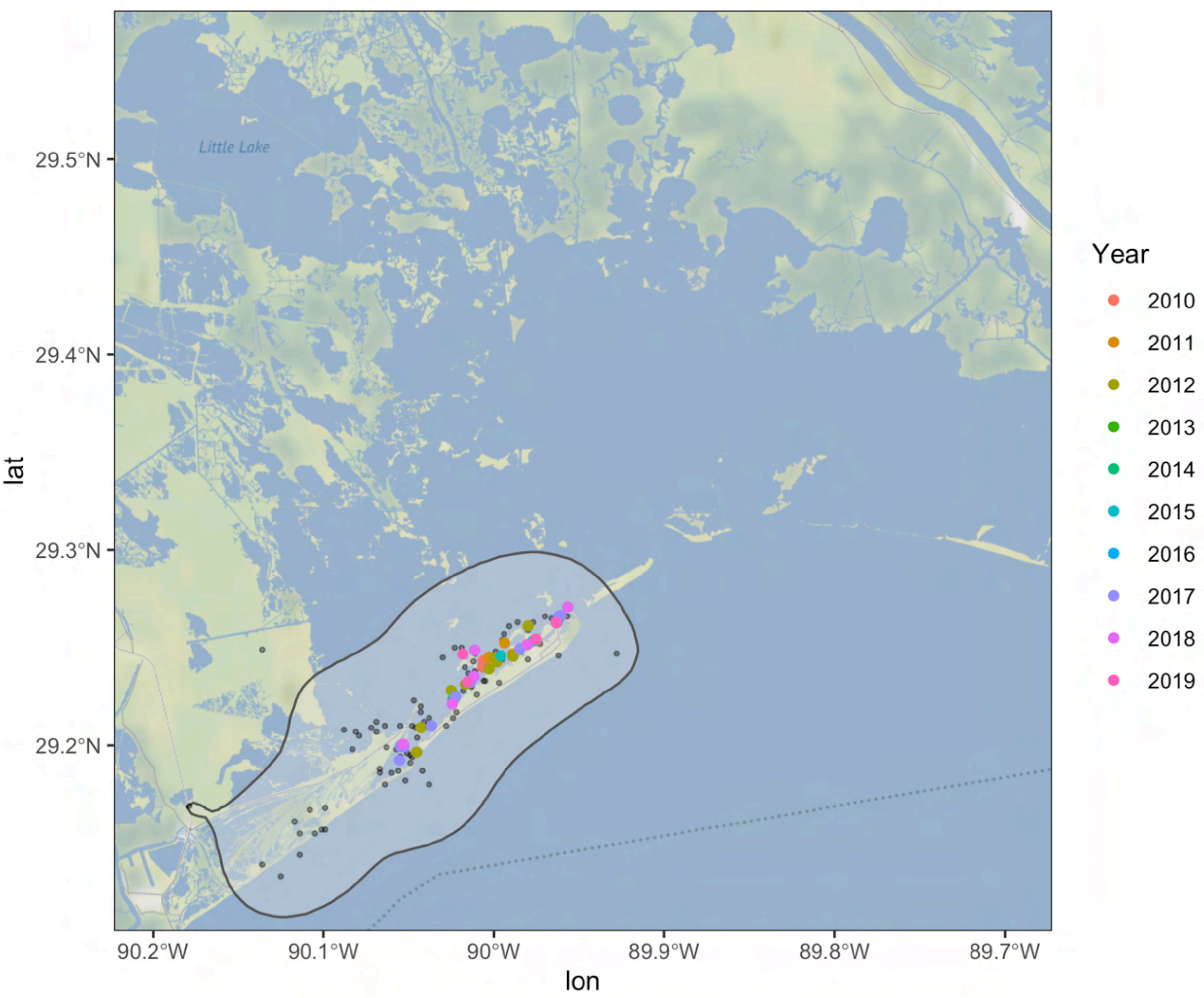

Little Lake
29.5°N
29.4°N
29.3°N
29.2°N
lat
90.2°W
90.1°W
90°W
89.9°W
89.8°W
89.7°W
lon
Year
2010
2011
2012
2013
2014
2015
2016
2017
2018
2019

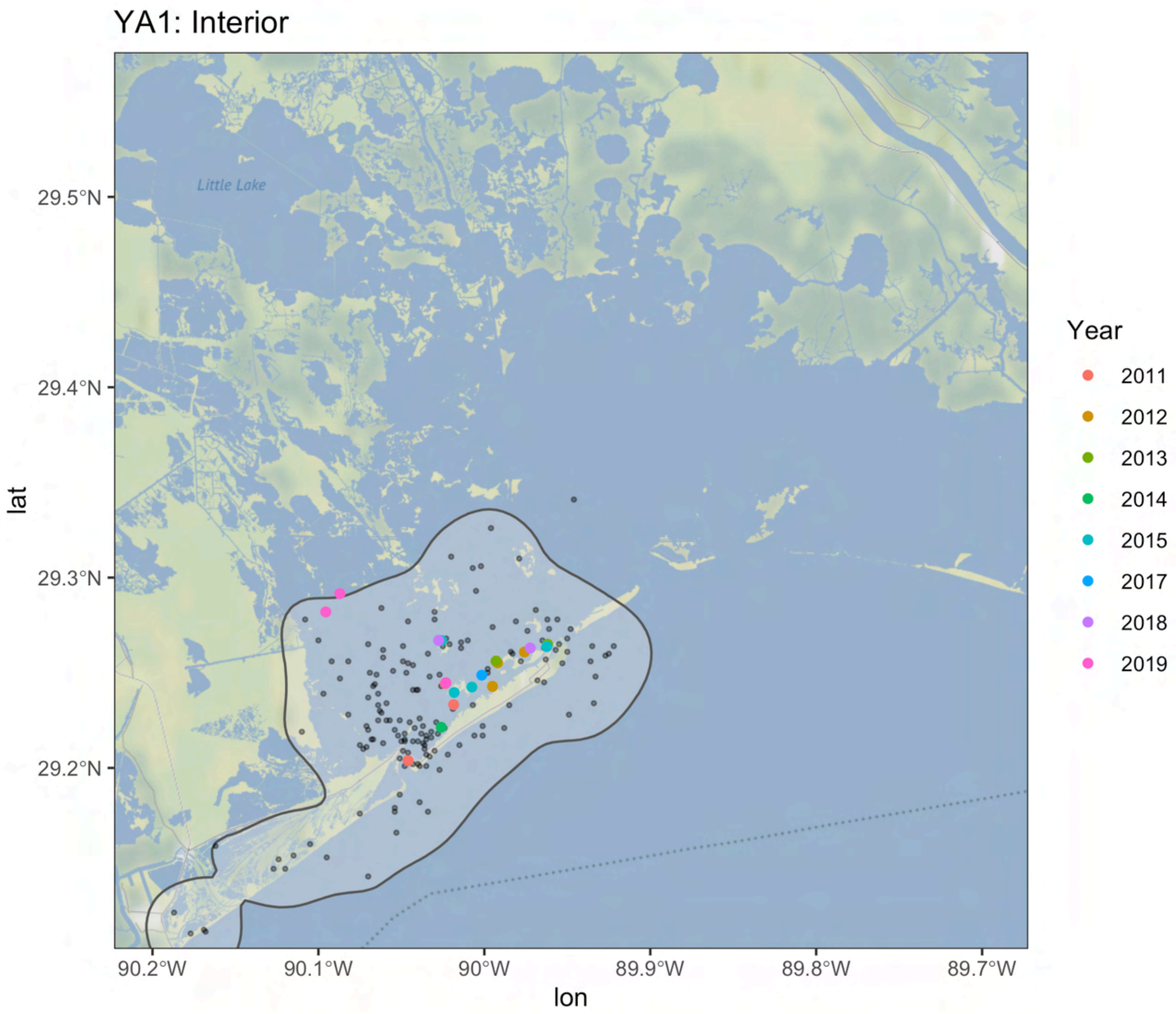
YA1: Interior
Little Lake
29.5°N
29.4°N
29.3°N
29.2°N
lat
90.2°W
90.1°W
90°W
89.9°W
89.8°W
89.7°W
lon
Year
2011
2012
2013
2014
2015
2017
2018
2019

YA3: Interior

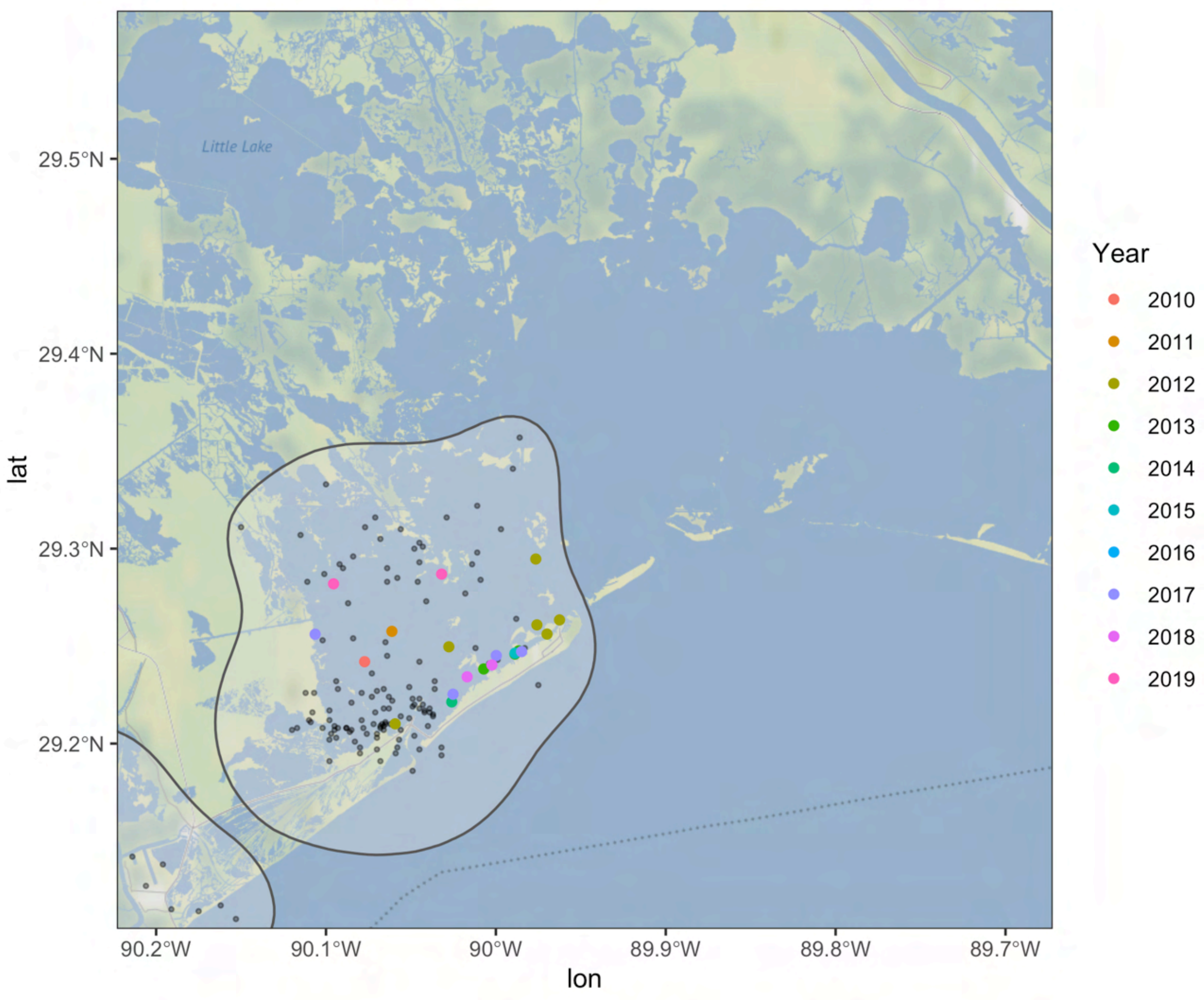

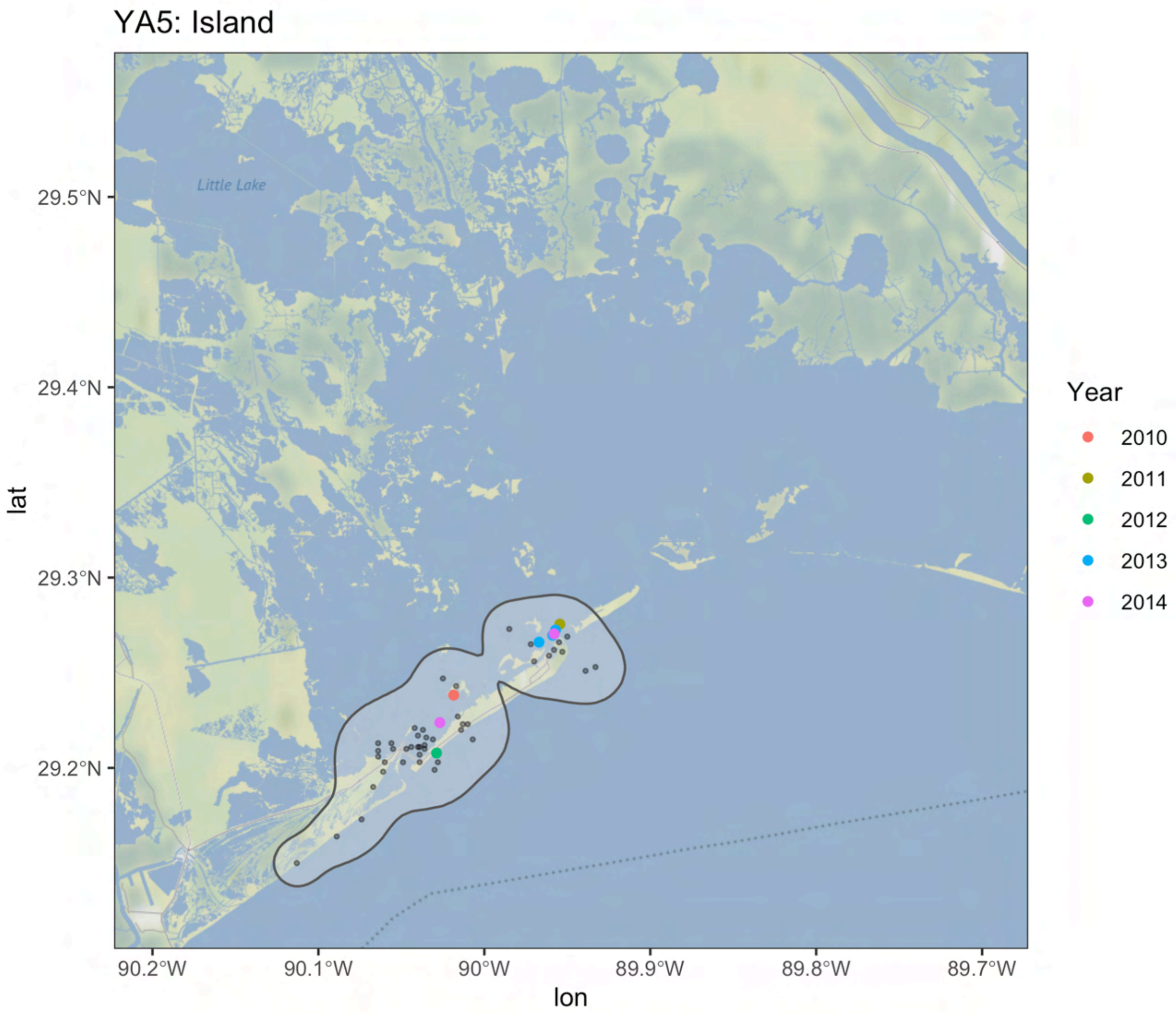
YA5: Island
Little Lake
29.5°N
29.4°N
29.3°N
29.2°N
lat
90.2°W
90.1°W
90°W
89.9°W
89.8°W
89.7°W
lon
Year
2010
2011
2012
2013
2014

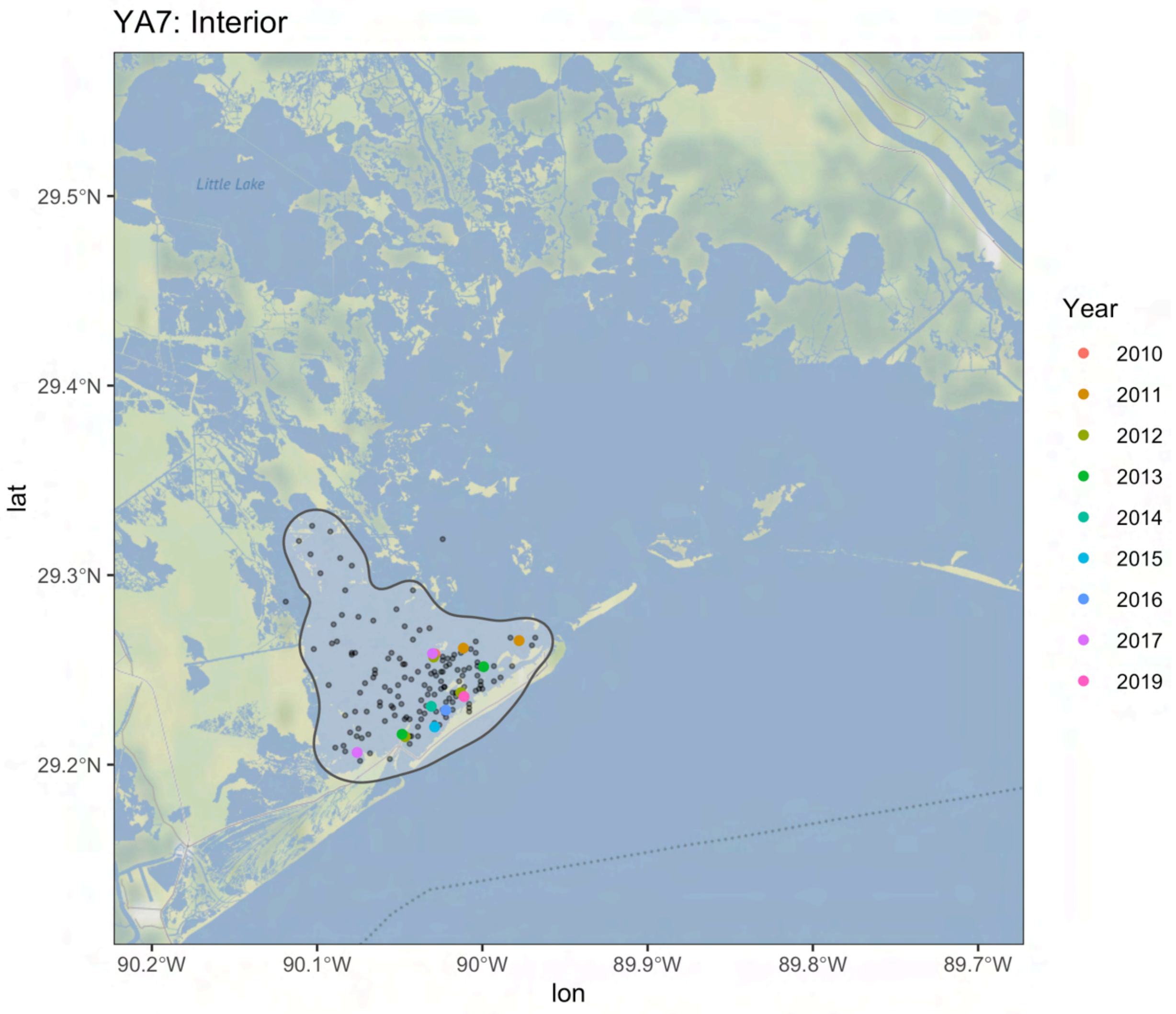
YA7: Interior
Little Lake
29.5°N
29.4°N
29.3°N
29.2°N
lat
90.2°W
90.1°W
90°W
89.9°W
89.8°W
89.7°W
lon
Year
2010
2011
2012
2013
2014
2015
2016
2017
2019

YF0: Interior

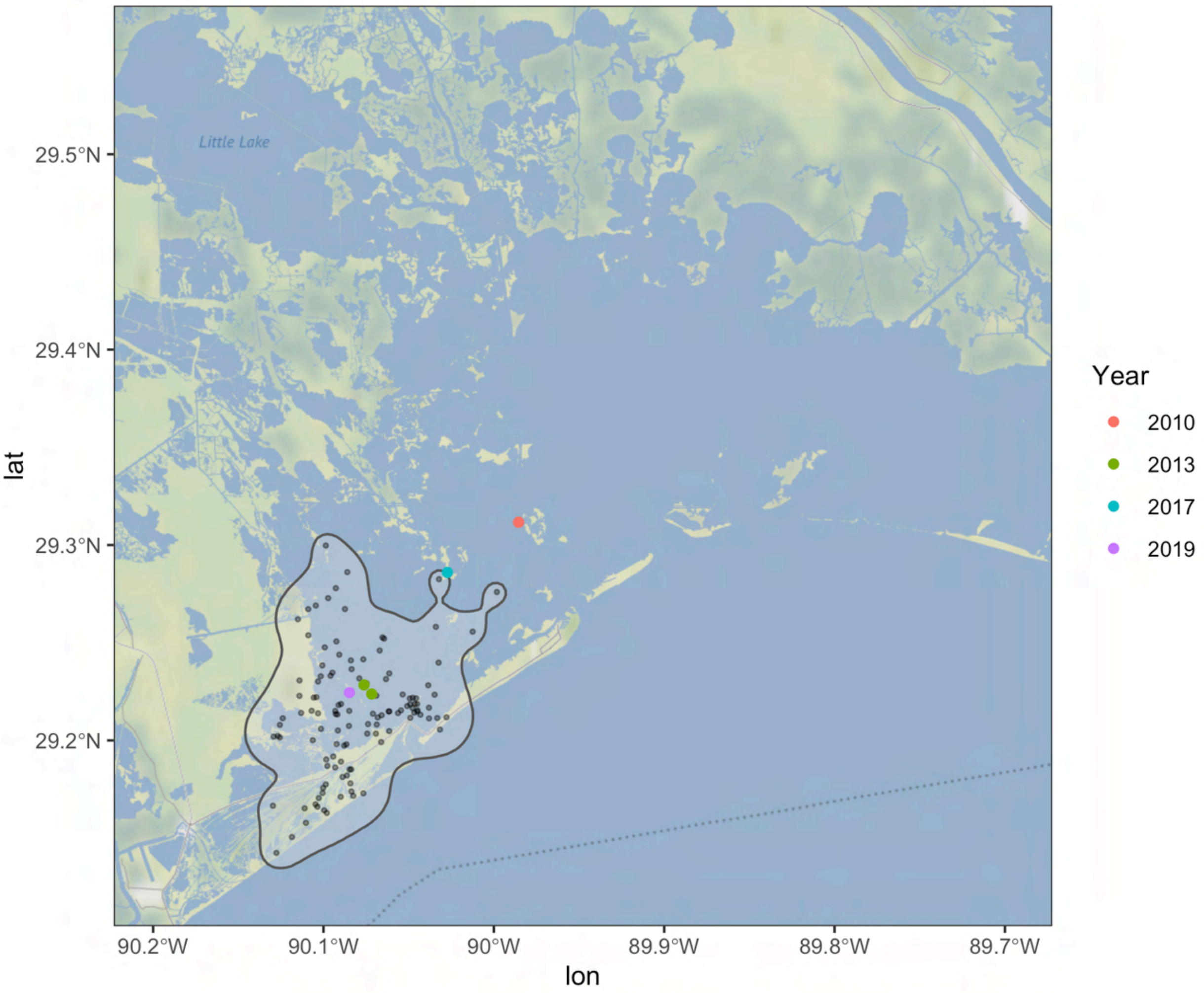

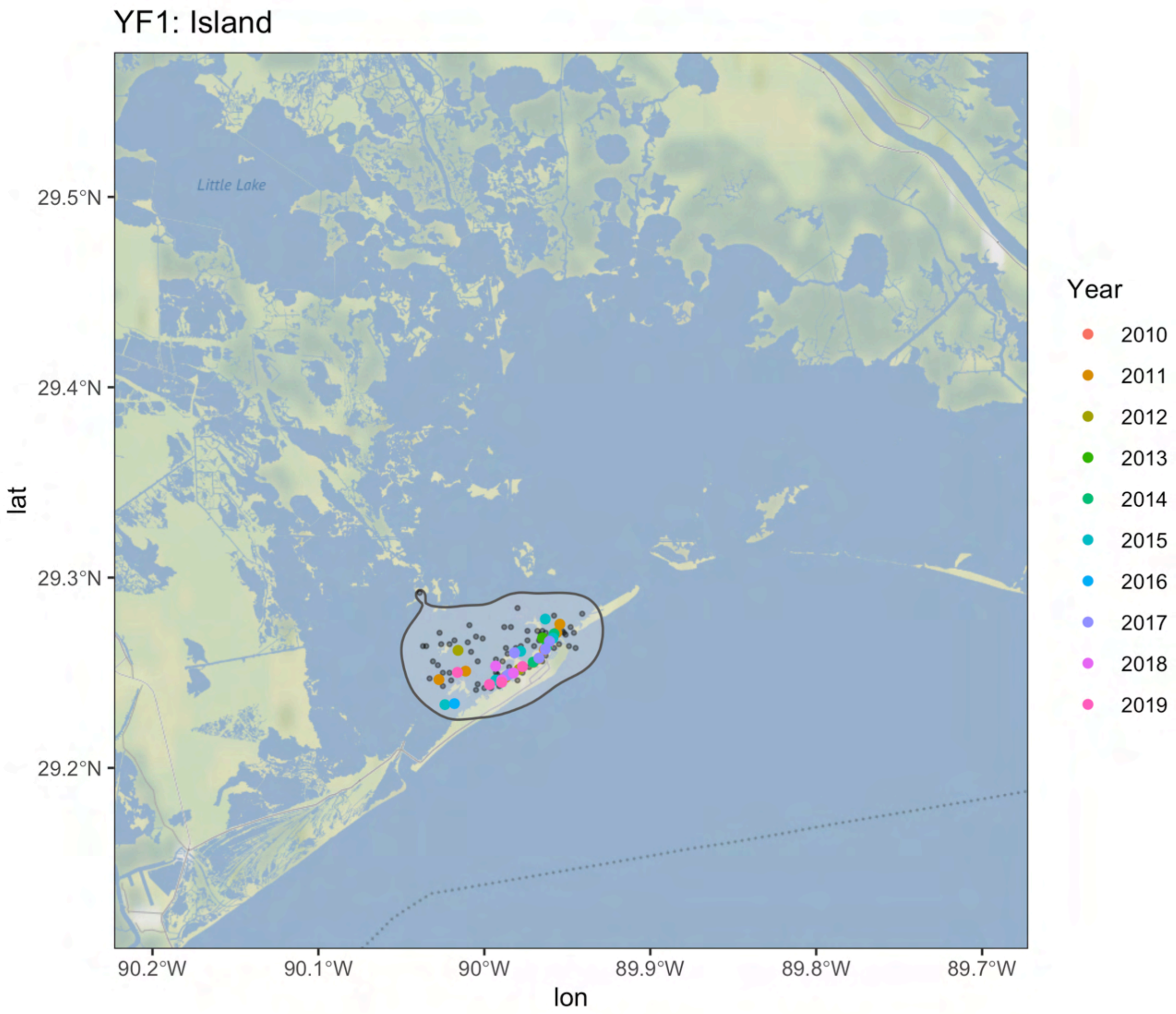
YF1: Island
Little Lake
29.5°N
29.4°N
29.3°N
29.2°N
lat
90.2°W
90.1°W
90°W
89.9°W
89.8°W
89.7°W
lon
Year
2010
2011
2012
2013
2014
2015
2016
2017
2018
2019

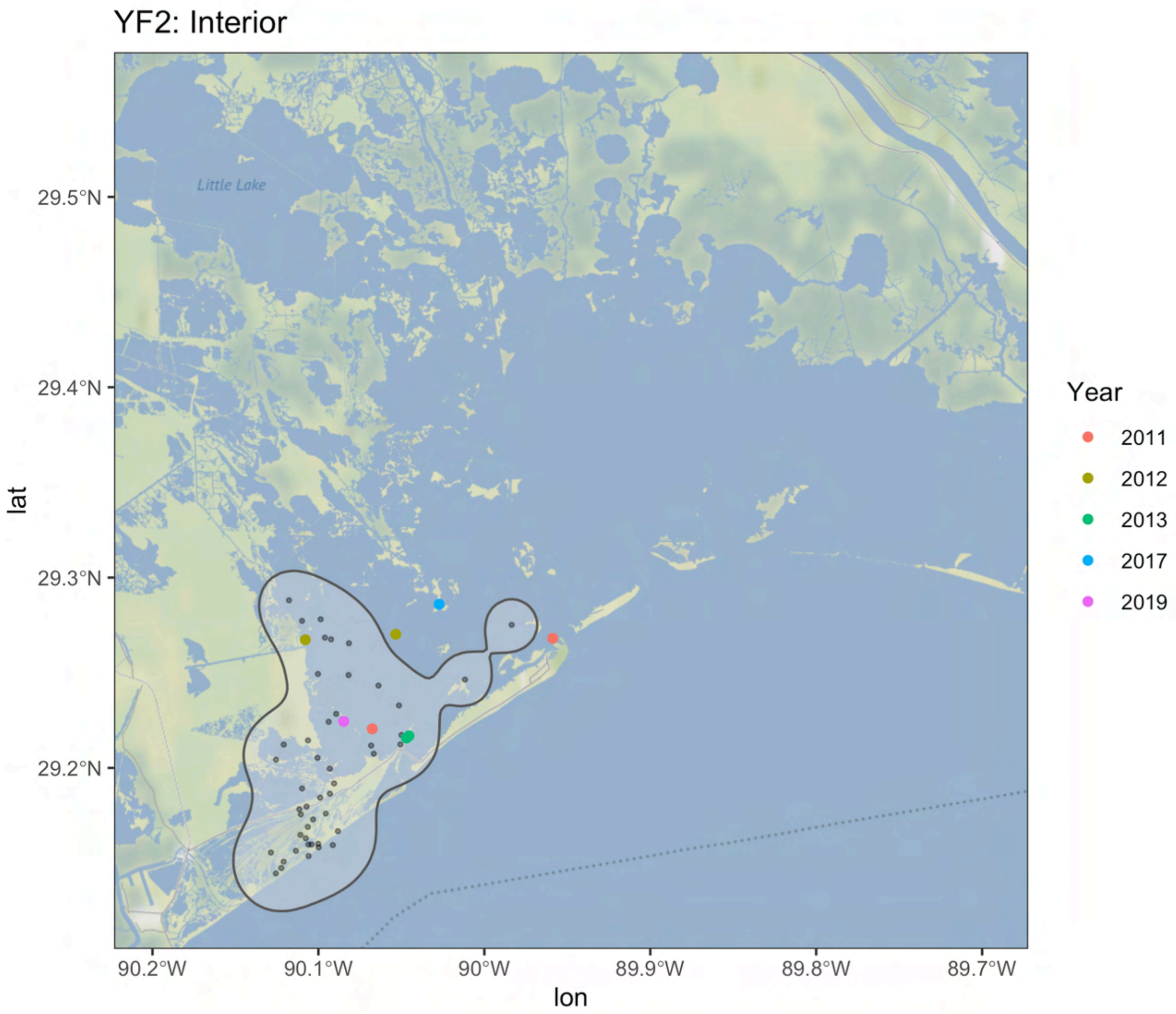
YF2: Interior
Little Lake
29.5°N
29.4°N
29.3°N
29.2°N
lat
90.2°W
90.1°W
90°W
89.9°W
89.8°W
89.7°W
lon
Year
2011
2012
2013
2017
2019

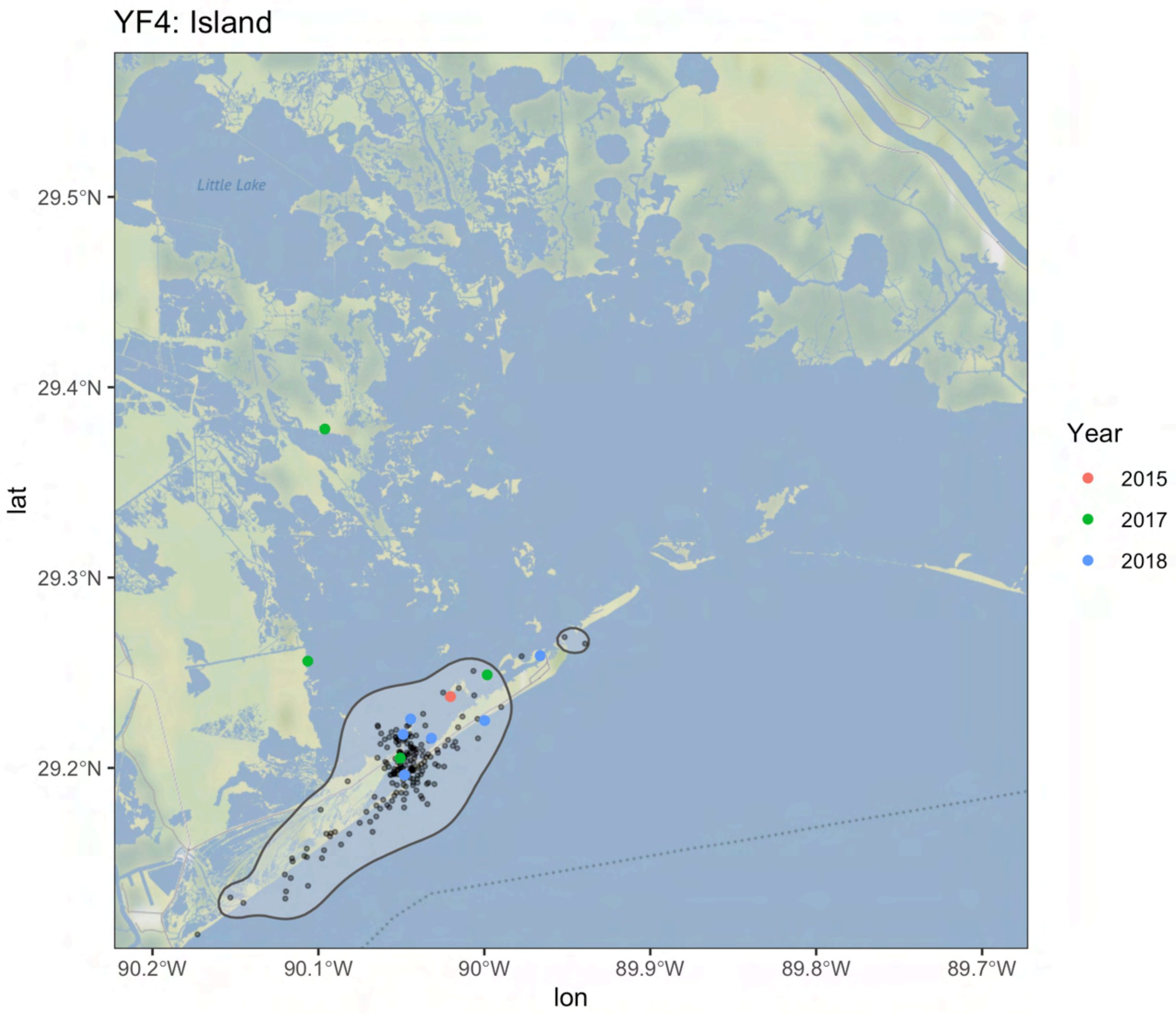

YF4: Island
Little Lake
29.5°N
29.4°N
29.3°N
29.2°N
lat
90.2°W
90.1°W
90°W
89.9°W
89.8°W
89.7°W
lon
Year
2015
2017
2018

YJ1: Island

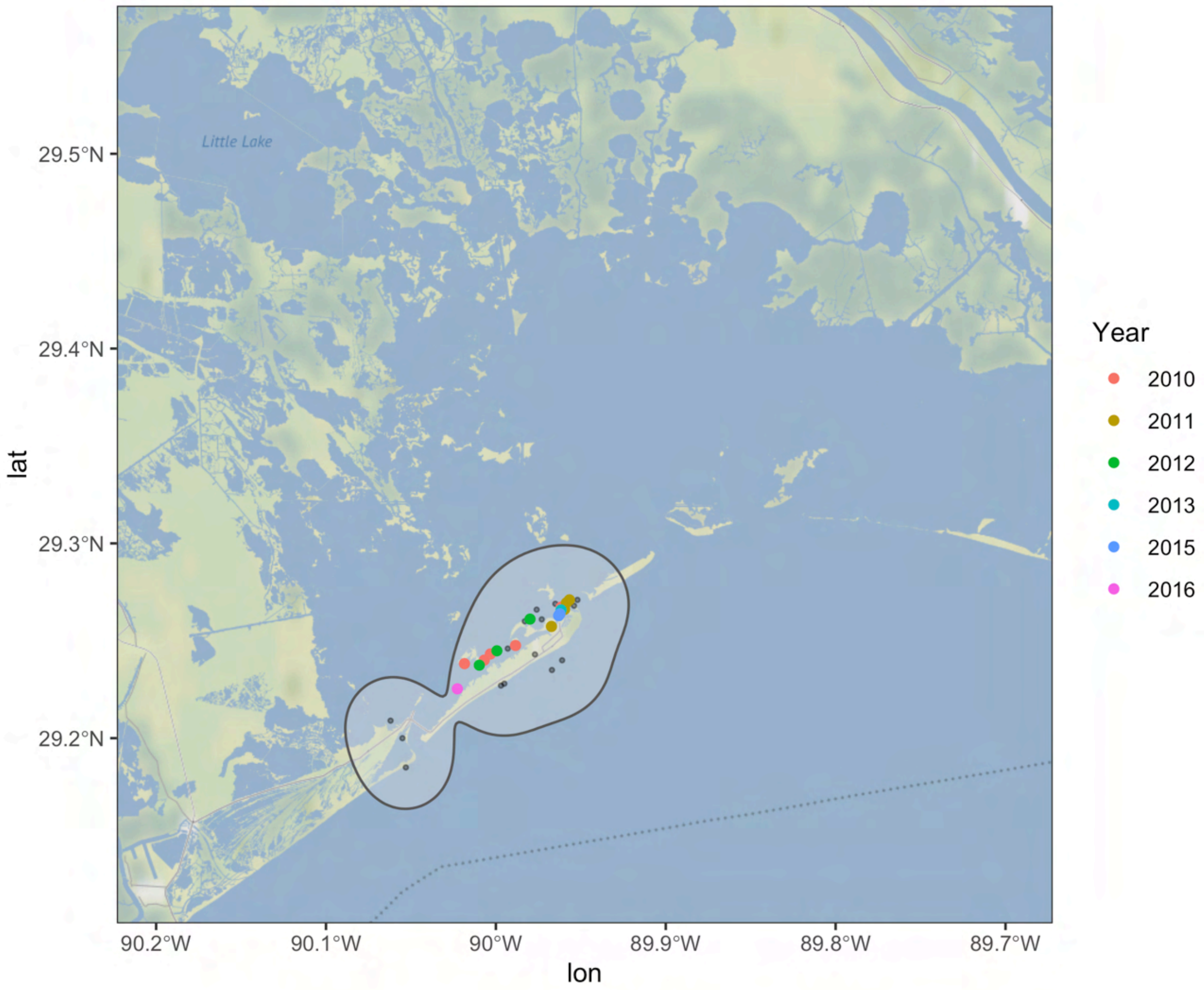

Little Lake
29.5°N
29.4°N
29.3°N
29.2°N
lat
90.2°W
90.1°W
90°W
89.9°W
89.8°W
89.7°W
lon
Year
2010
2011
2012
2013
2015
2016

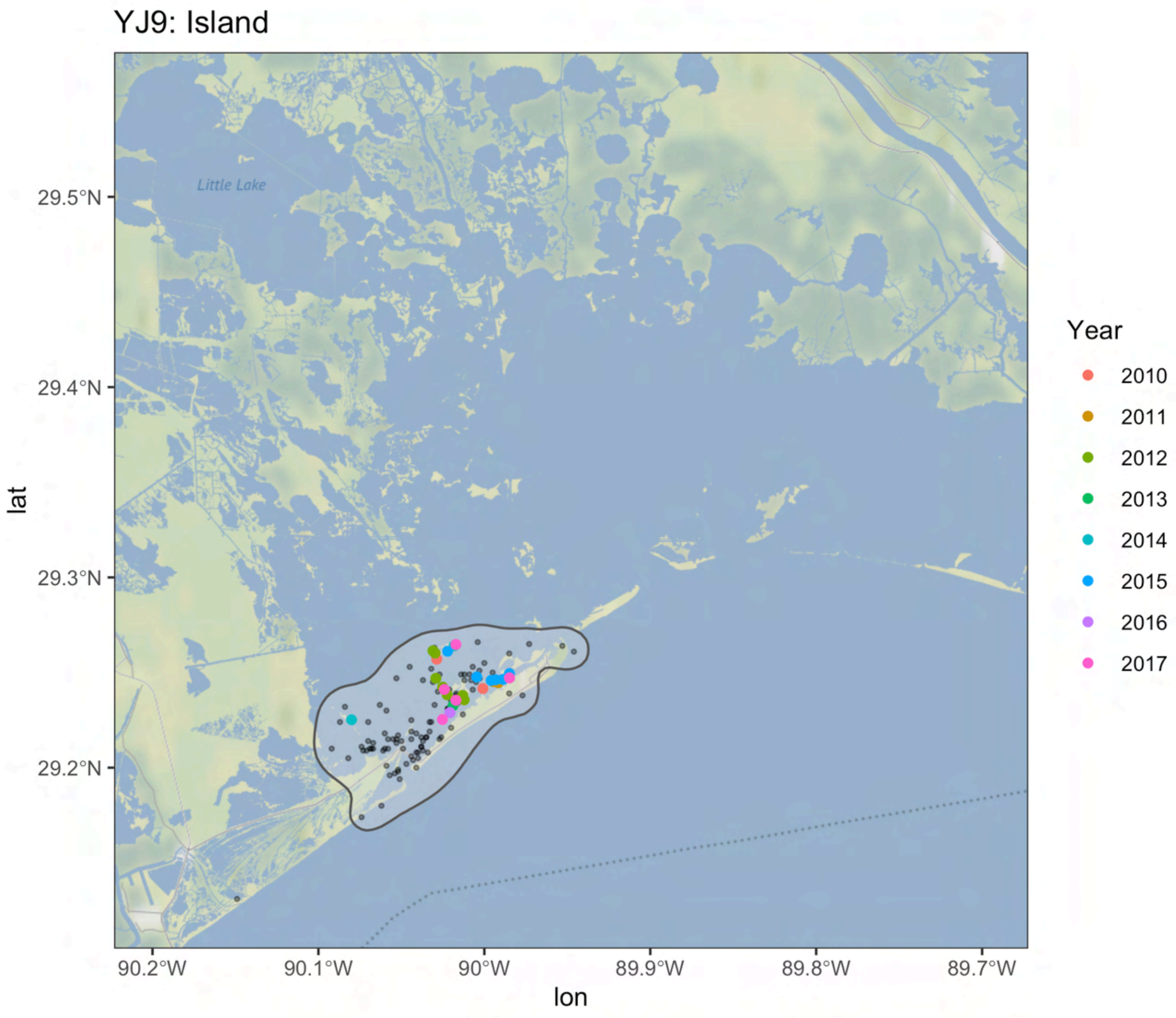

YJ9: Island
Little Lake
29.5°N
29.4°N
29.3°N
29.2°N
lat
90.2°W
90.1°W
90°W
89.9°W
89.8°W
89.7°W
lon
Year
2010
2011
2012
2013
2014
2015
2016
2017

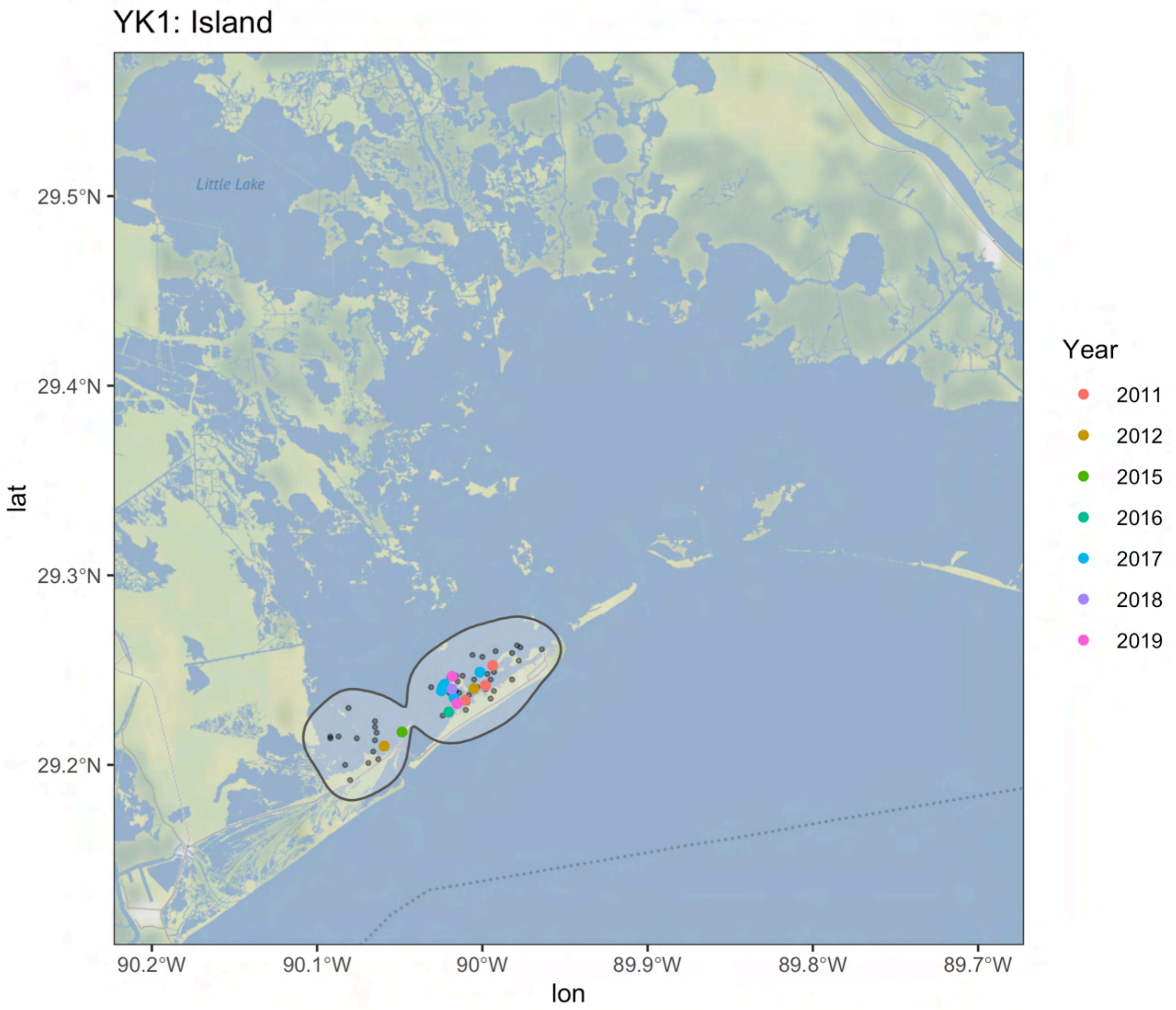

YK1: Island
Little Lake
29.5°N
29.4°N
29.3°N
29.2°N
lat
90.2°W
90.1°W
90°W
89.9°W
89.8°W
89.7°W
lon
Year
2011
2012
2015
2016
2017
2018
2019

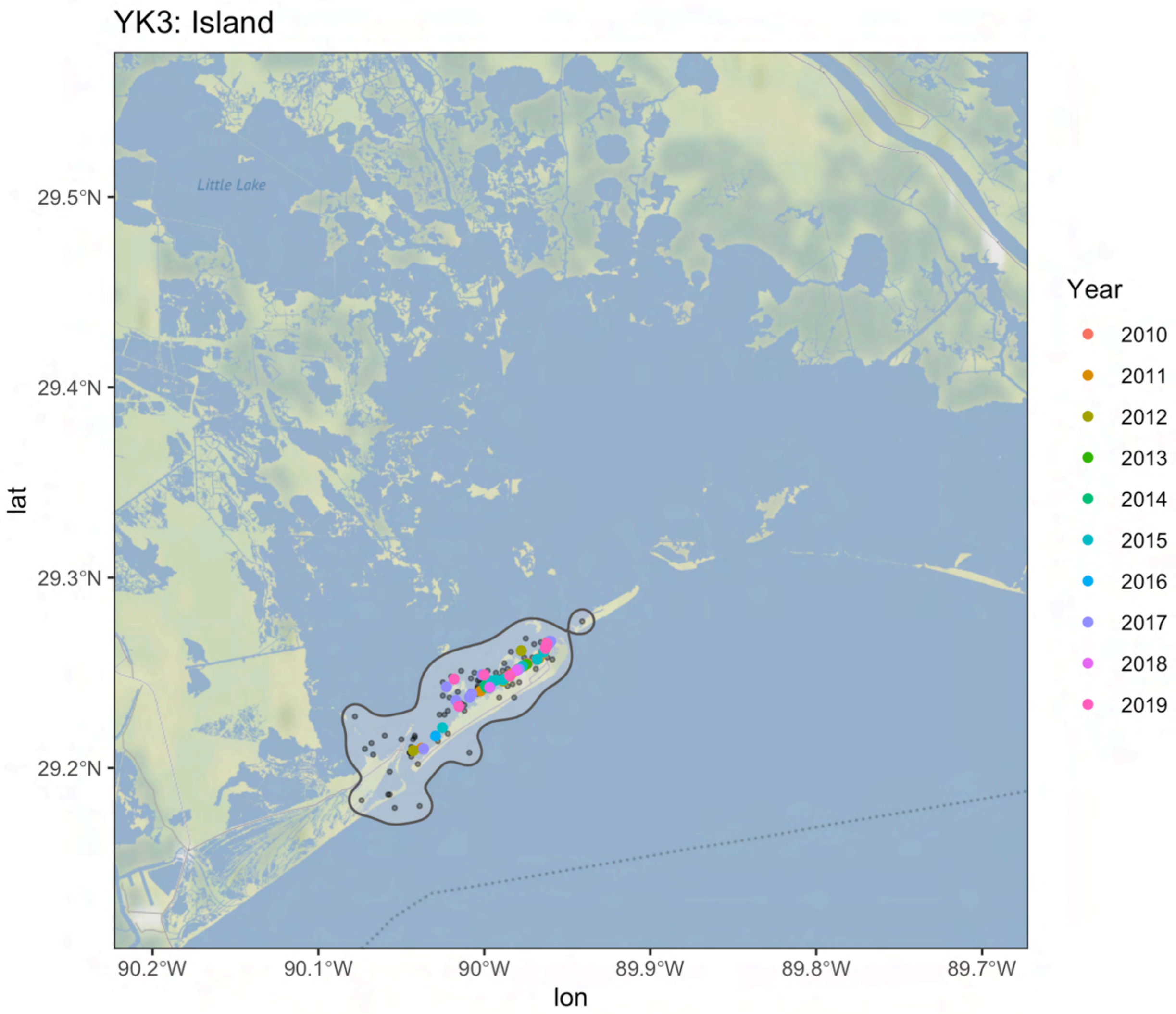
YK3: Island
Little Lake
29.5°N
29.4°N
29.3°N
29.2°N
lat
90.2°W
90.1°W
90°W
89.9°W
89.8°W
89.7°W
lon
Year
2010
2011
2012
2013
2014
2015
2016
2017
2018
2019

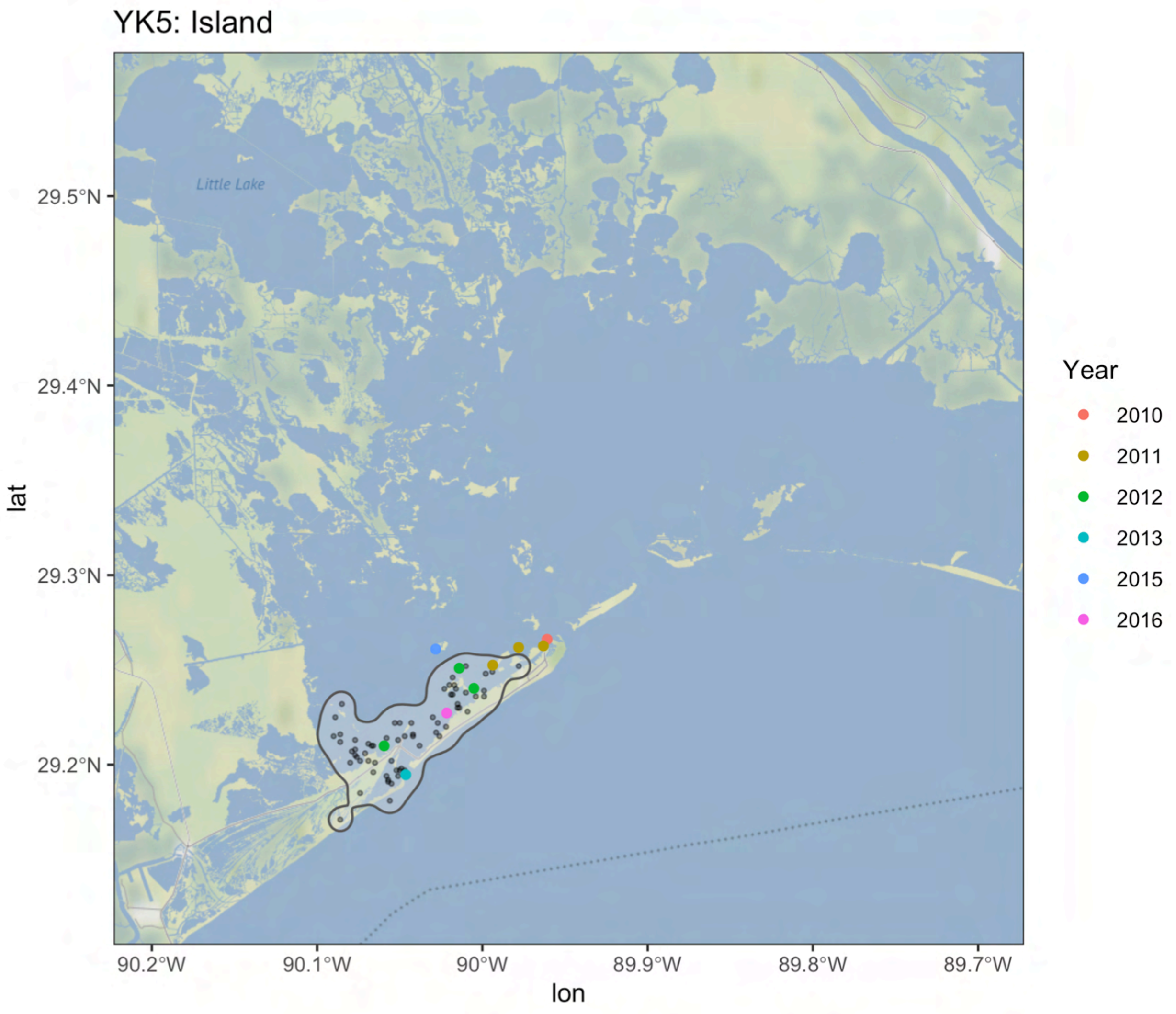
YK5: Island
Little Lake
29.5°N
29.4°N
29.3°N
29.2°N
lat
90.2°W
90.1°W
90°W
89.9°W
89.8°W
89.7°W
lon
Year
2010
2011
2012
2013
2015
2016

YK9: Interior

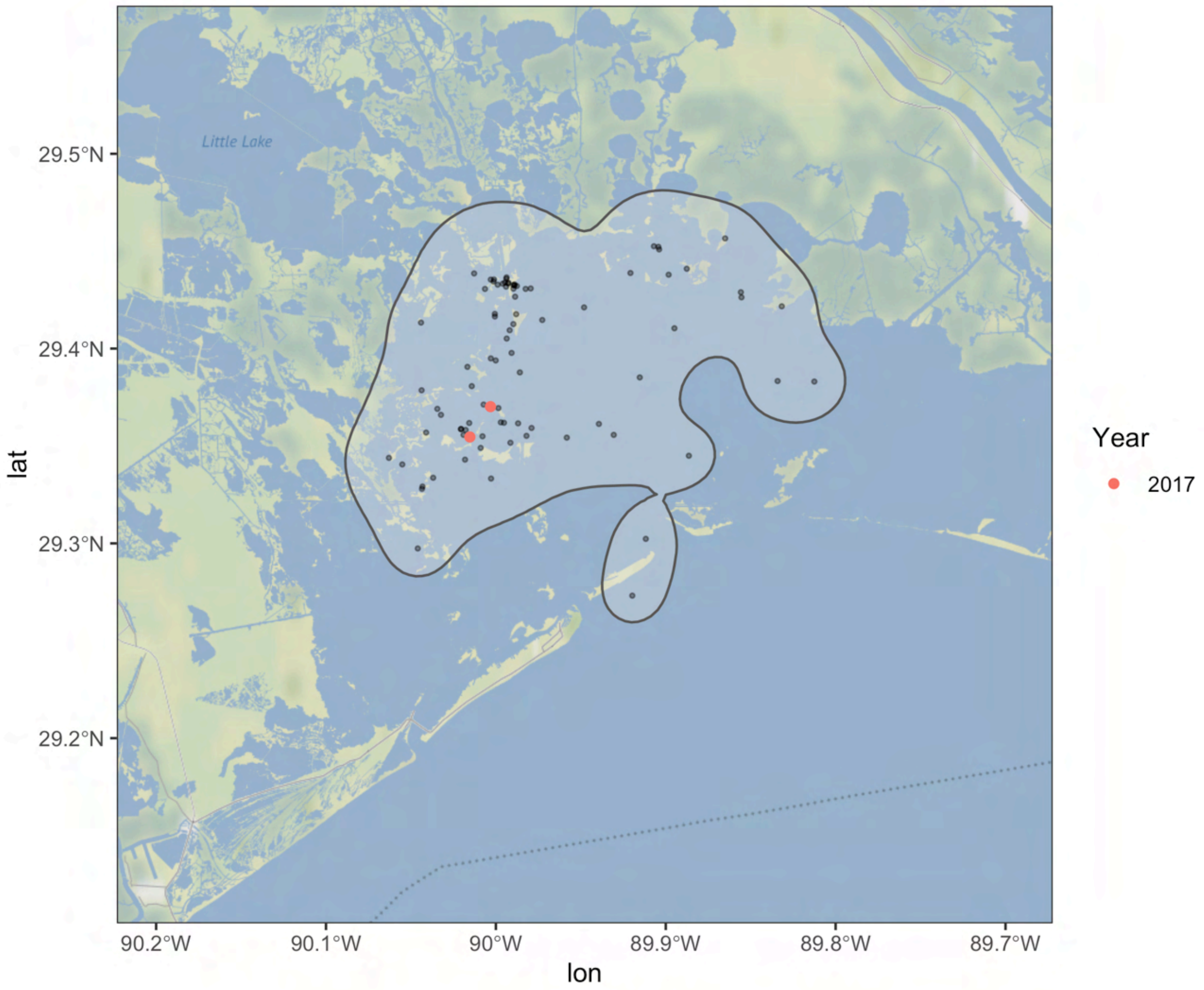

YN1: Interior

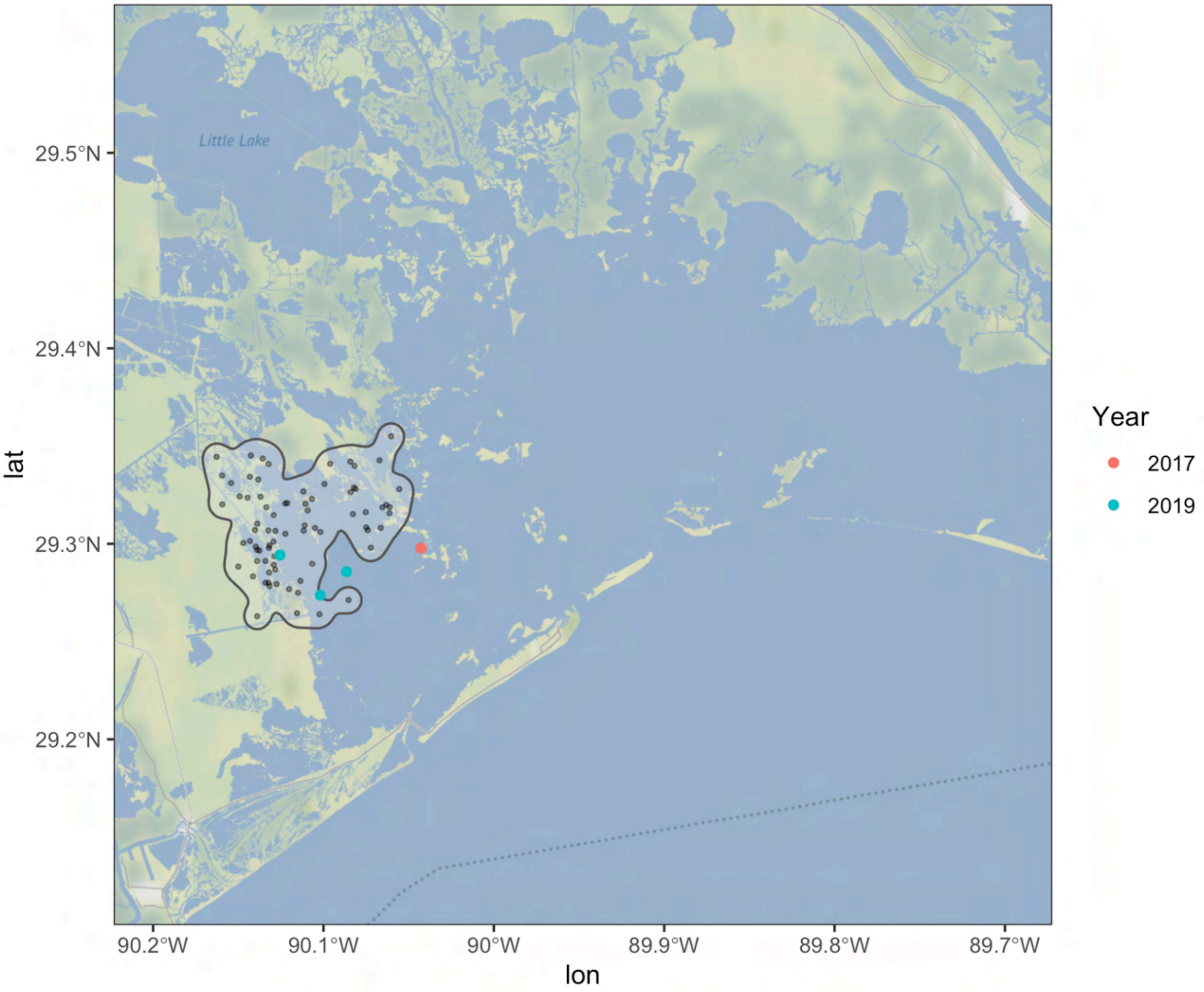

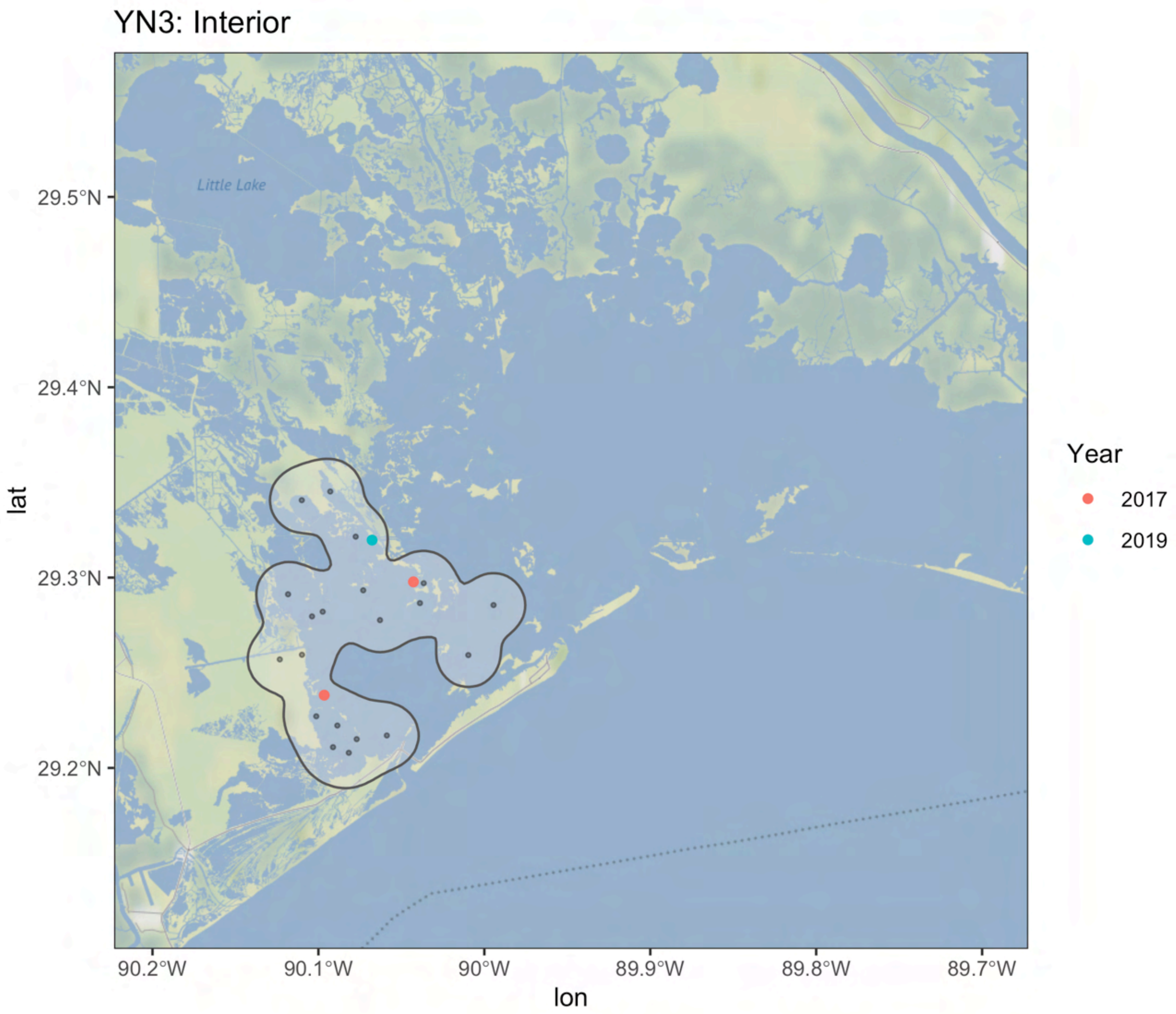
YN3: Interior
Little Lake
29.5°N
29.4°N
29.3°N
29.2°N
lat
90.2°W
90.1°W
90°W
89.9°W
89.8°W
89.7°W
lon
Year
2017
2019

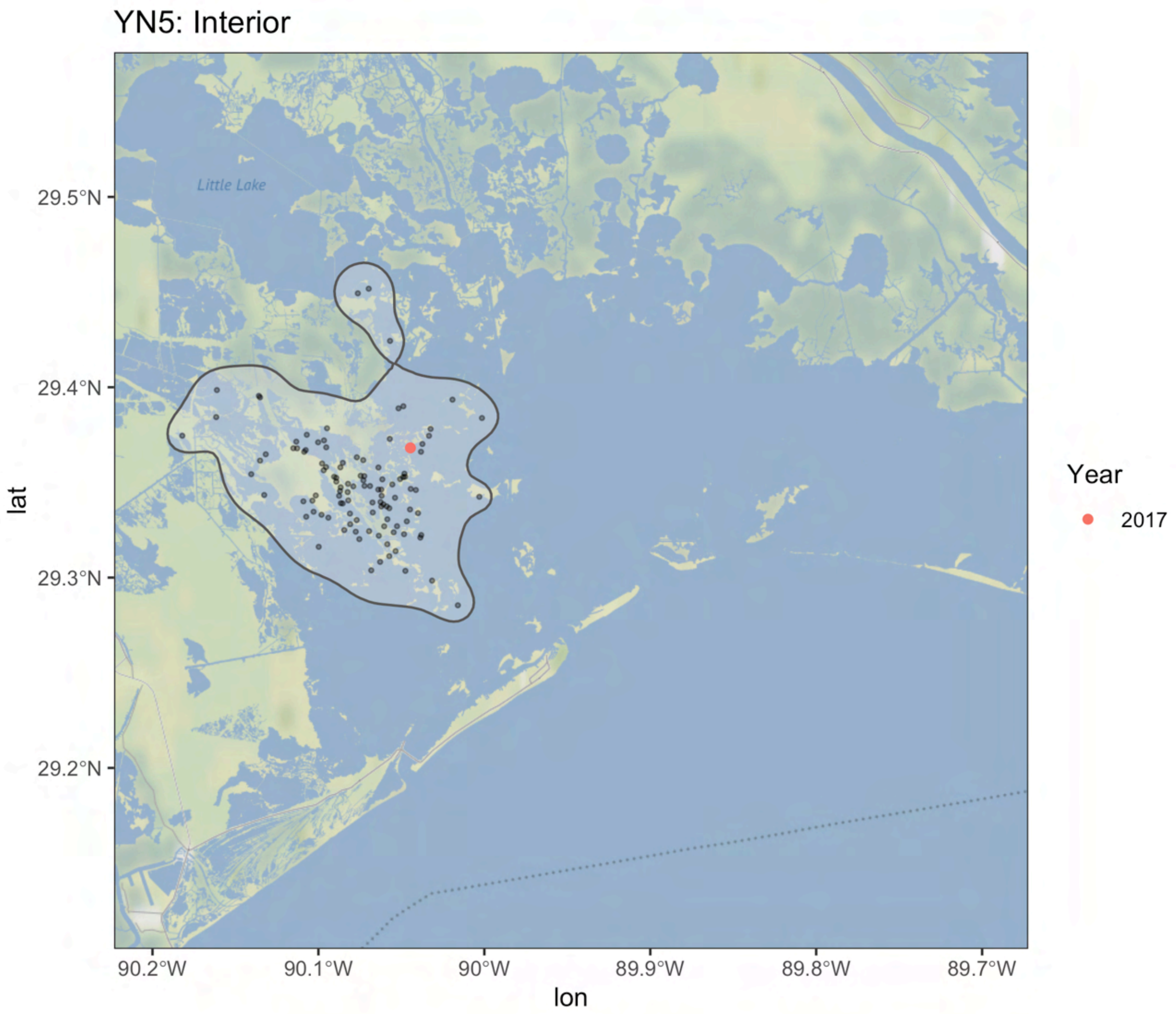
YN5: Interior
Little Lake
29.5°N
29.4°N
29.3°N
29.2°N
lat
90.2°W
90.1°W
90°W
89.9°W
89.8°W
89.7°W
lon
Year
2017

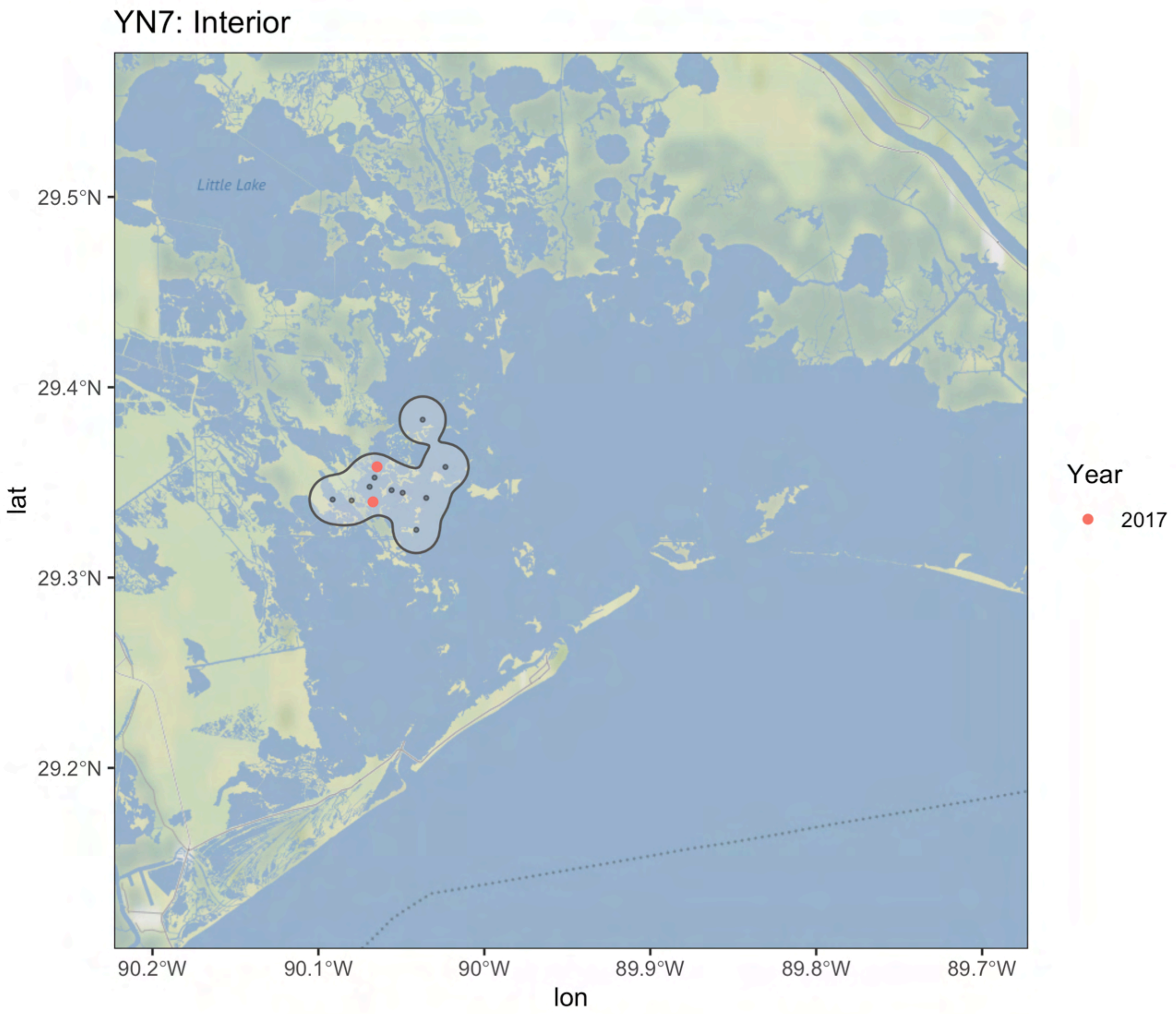
YN7: Interior
Little Lake
29.5°N
29.4°N
29.3°N
29.2°N
lat
90.2°W
90.1°W
90°W
89.9°W
89.8°W
89.7°W
lon
Year
2017

YN9: Interior

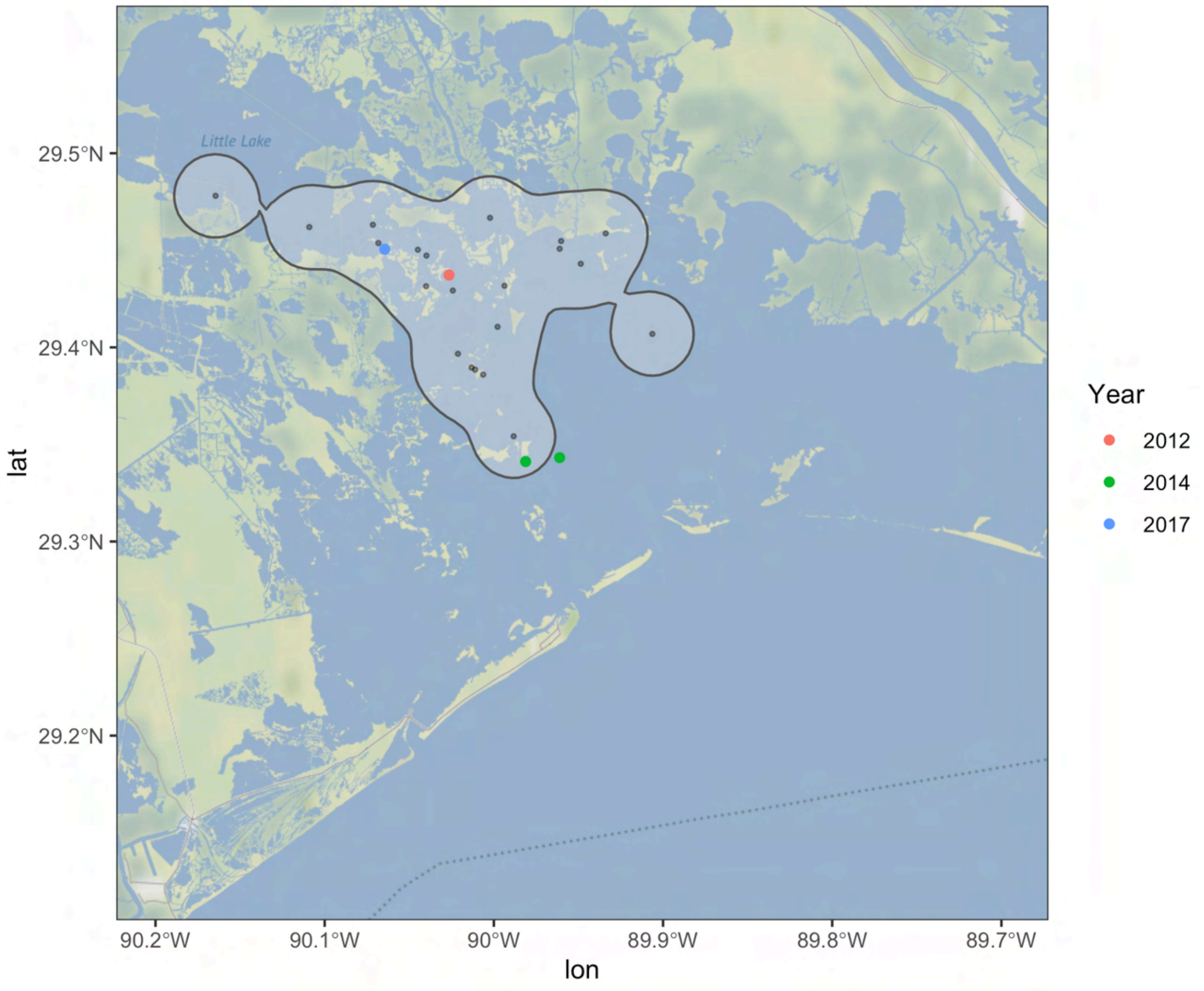

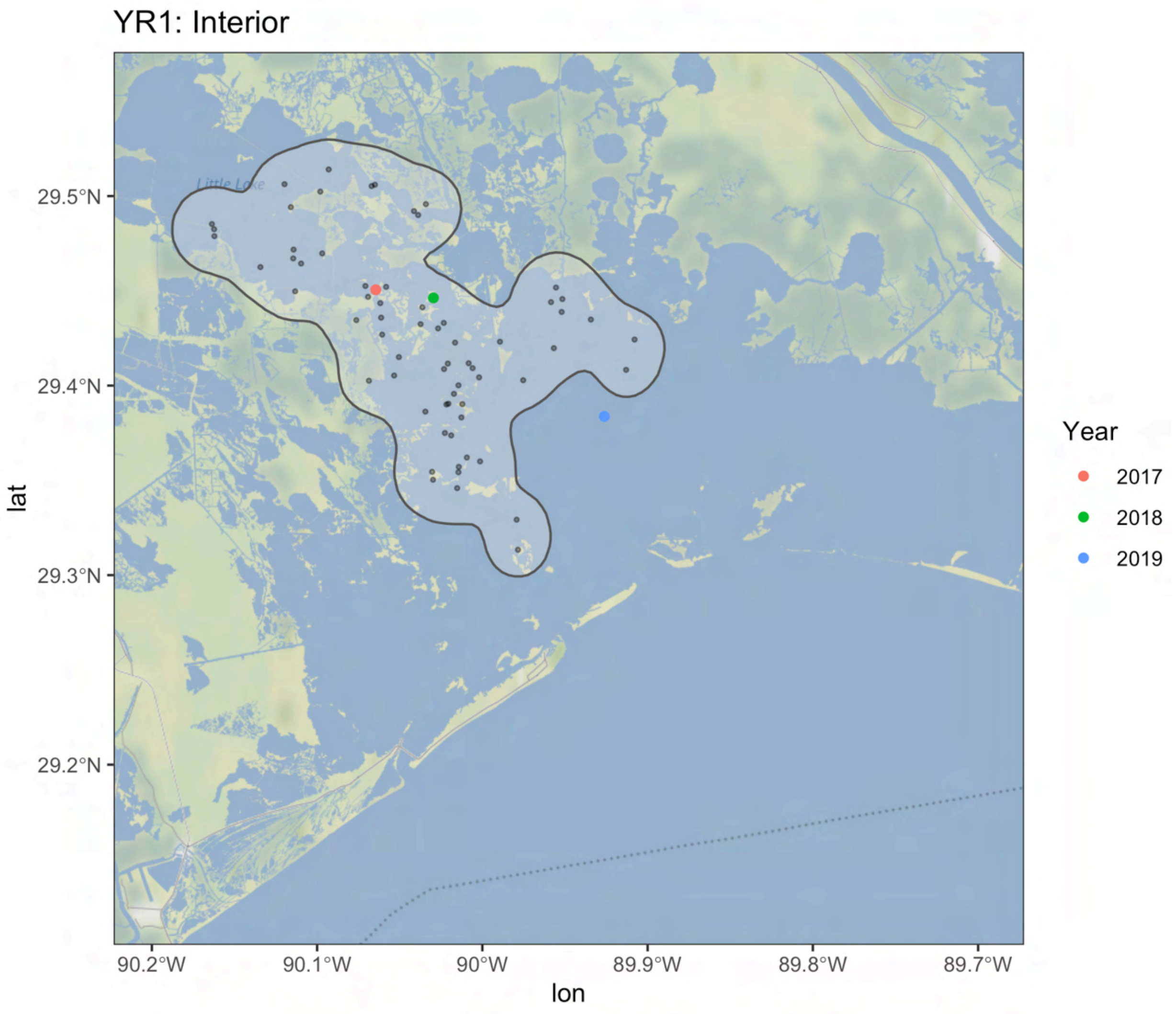
YR1: Interior
lat
29.5°N
29.4°N
29.3°N
29.2°N
90.2°W
90.1°W
90°W
89.9°W
89.8°W
89.7°W
lon
Year
2017
2018
2019

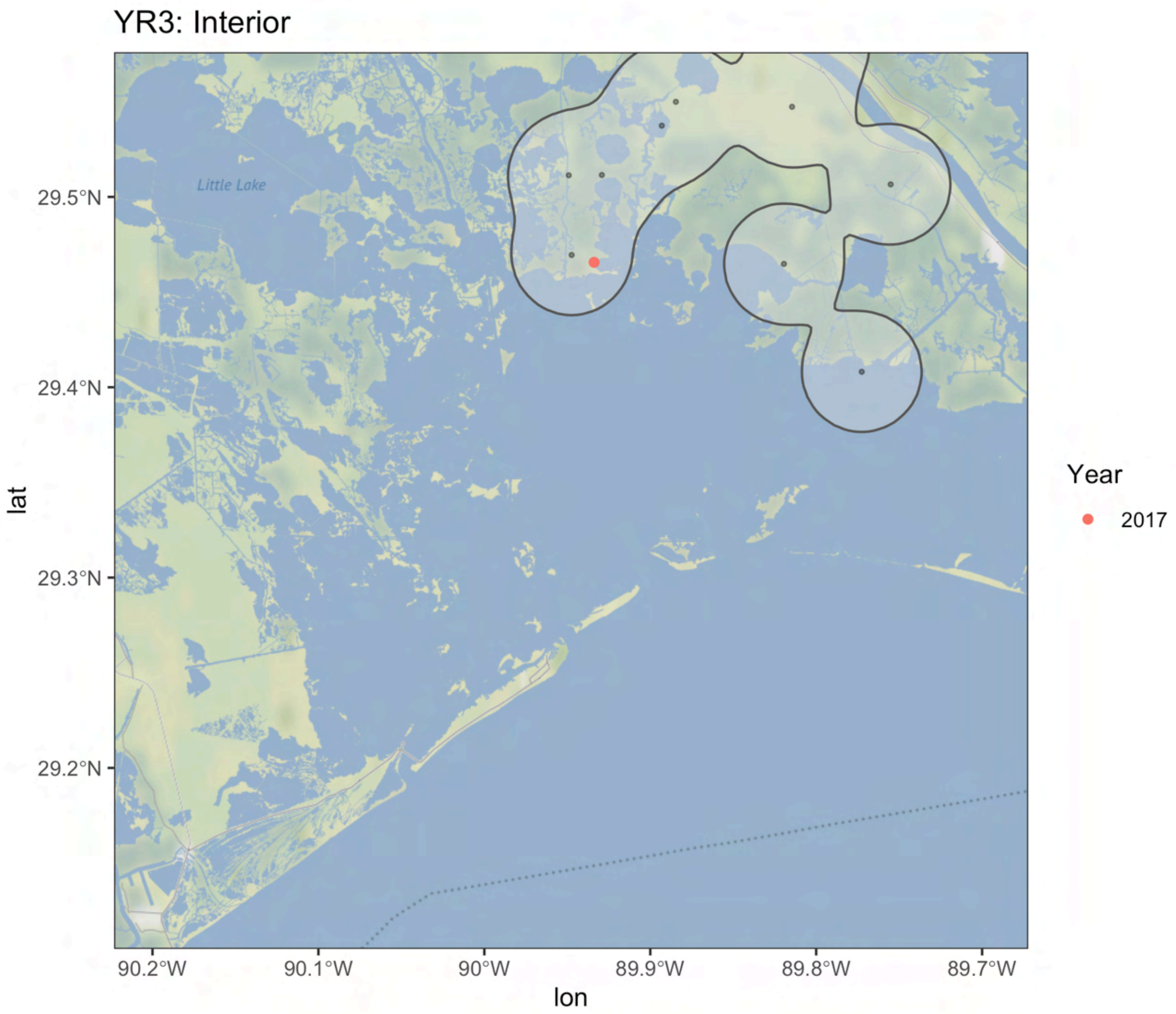

YR3: Interior
Little Lake
29.5°N
29.4°N
29.3°N
29.2°N
lat
90.2°W
90.1°W
90°W
89.9°W
89.8°W
89.7°W
lon
Year
2017

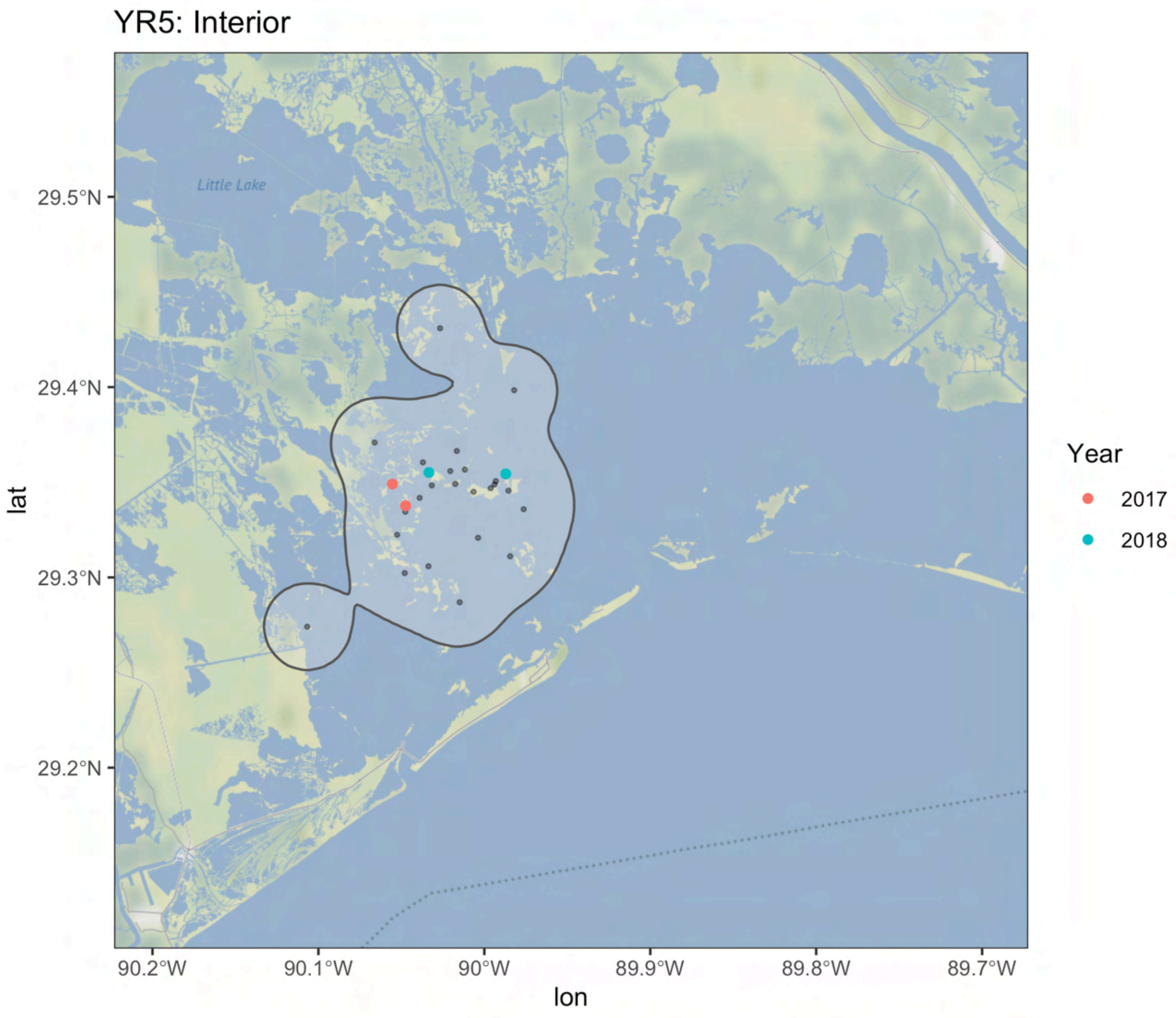
YR5: Interior
Little Lake
29.5°N
29.4°N
29.3°N
29.2°N
lat
90.2°W
90.1°W
90°W
89.9°W
89.8°W
89.7°W
lon
Year
2017
2018

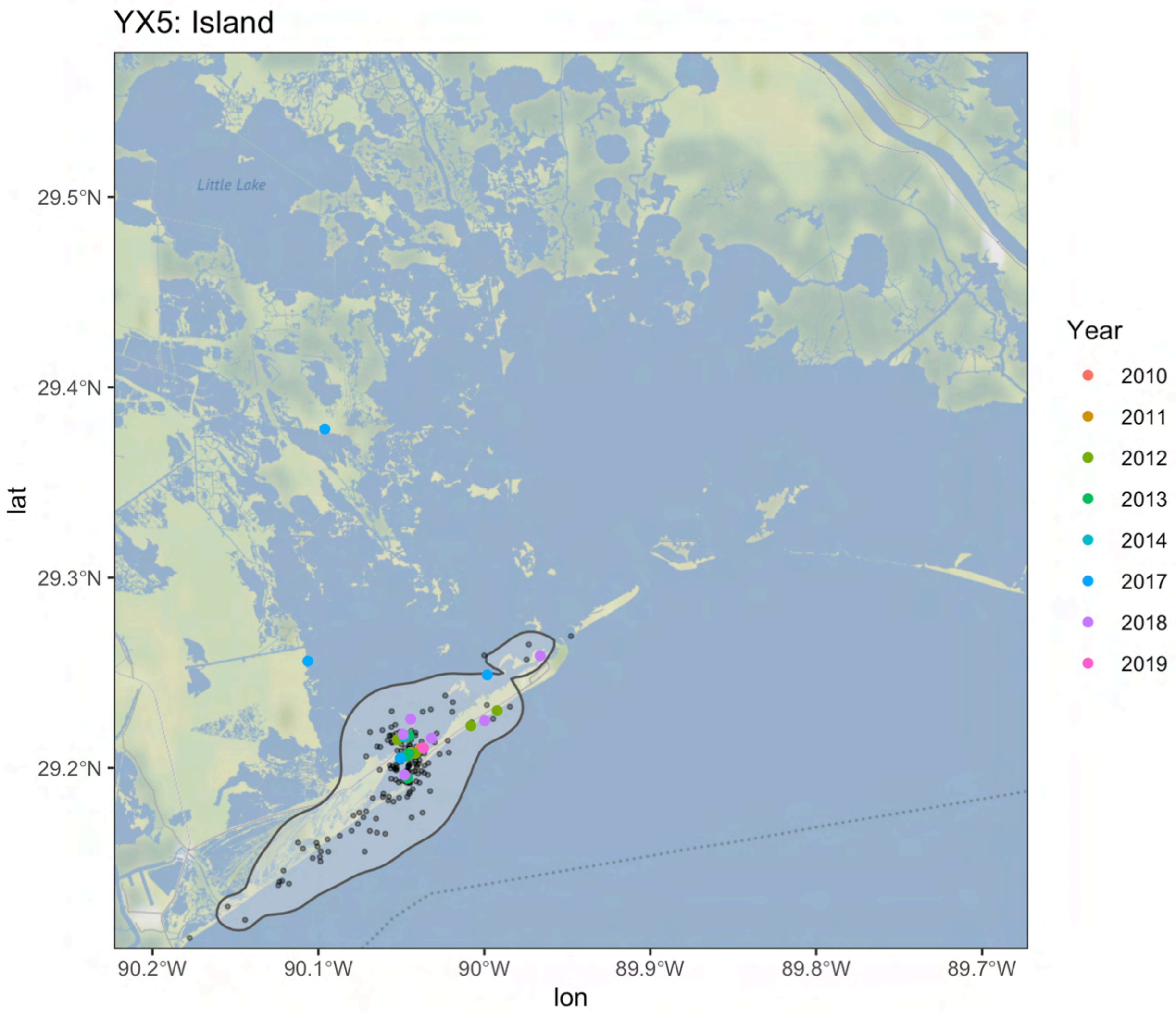
YX5: Island
Little Lake
29.5°N
29.4°N
29.3°N
29.2°N
lat
90.2°W
90.1°W
90°W
89.9°W
89.8°W
89.7°W
lon
Year
2010
2011
2012
2013
2014
2017
2018
2019
