# Supplementary material for: High site-fidelity in common bottlenose dolphins despite low salinity exposure and associated indicators of compromised health
Source: PLoS One. 2021 Sep 30;16(9):e0258031. doi: 10.1371/journal.pone.0258031 (PMC8483354; doi:10.1371/journal.pone.0258031)
Supplement: S5 File — The data recording sheet for assessing skin health during health assessments in the Barataria Basin. (PDF) [file pone.0258031.s005.pdf]

**Date (mm/dd):_________2018**	**Set #: __________**	**FB:**

Skin Assessment

Epidermal Sloughing: ☐ None ☐ Generalized ☐ Focused

Rake Marks: ☐ 0 ☐ 1–2 ☐ 3–5 ☐ > 5

Indicate location(s) of lesions described using corresponding Lesion(s) Description #:

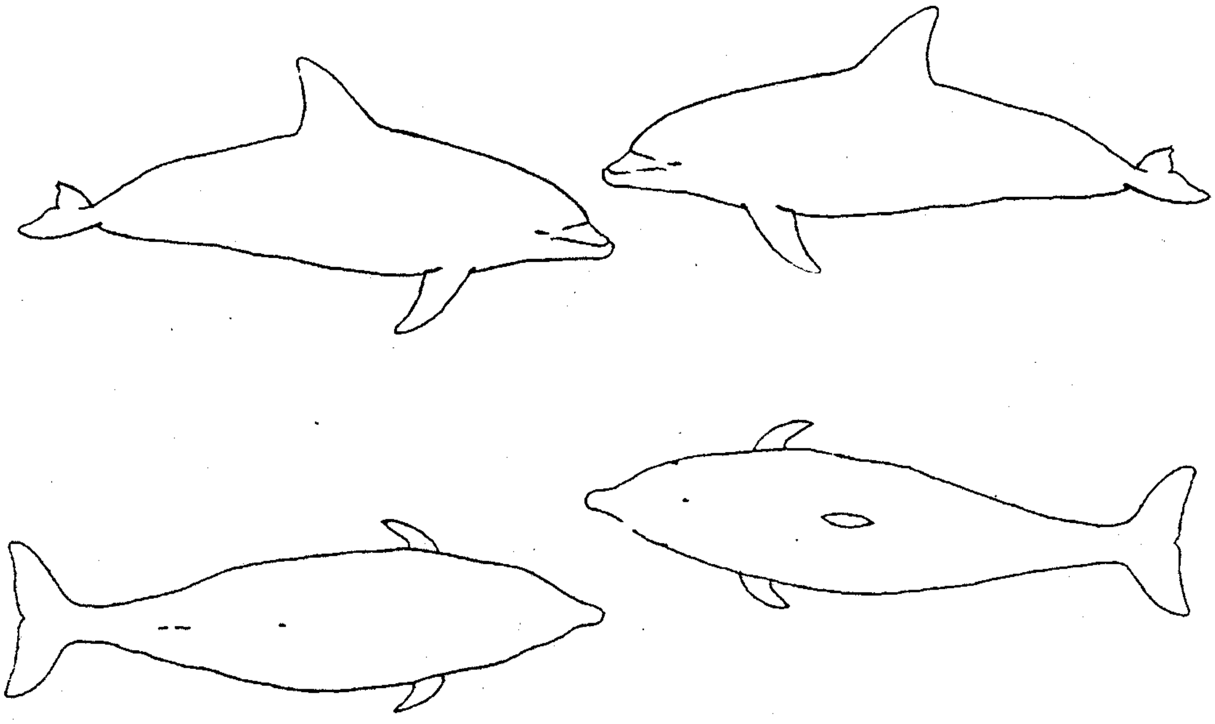

Comments:

____

____

____

____

____

Veterinarian __________________________ **Date** __________________

*Data Collector*______________________________

Date (mm/dd):_________2018 | **Set #: __________** | **FB:**

Lesion Descriptions

Lesion(s) Description #: ___________

Distribution: ☐ Single Lesion ☐ Multiple Lesions
Type: ☐ Fishery Int. ☐ Predator ☐ Other Traumatic ☐ Tattoo
☐ Lacaziosis ☐ Papilloma ☐ Pox ☐ Other Infectious ☐ Vesicular
Status: ☐ Active ☐ Inactive ☐ Active/Healing ☐ Healed ☐ NA
Color(s): ☐ Black ☐ Gray ☐ White ☐ Red ☐ Other: ______________
Shape(s): ☐ Pinhole ☐ Round ☐ Irregular ☐ Other: ______________
Site(s): ☐ Dorsal ☐ Lateral ☐ Ventral
Form(s): ☐ Flat ☐ Raised ☐ Depressed ☐ Ulcerated ☐ Cauliflower
☐ Other: ____
Biopsy: ☐ Frozen ☐ Histopath ☐ None
If biopsy taken: Vet ___________________ Size of punch _____________________

Single Lesion Only:
Size: ☐ < 1 cm^2 ☐ 1–3 cm^2 ☐ > 3 cm^2
Consistency: ☐ Firm ☐ Soft ☐ Gelatinous ☐ Slough ☐ Other: _______________

Multiple Lesions Only:
Pattern: ☐ Focal ☐ Multifocal ☐ Multifocal to Coalescing ☐ Diffuse ☐ Other
Severity: ☐ Mild (≤ 10%) ☐ Moderate (11–50%) ☐ Severe (> 50%) ☐ NA

Lesion(s) Description #: ___________

Distribution: ☐ Single Lesion ☐ Multiple Lesions
Type: ☐ Fishery Int. ☐ Predator ☐ Other Traumatic ☐ Tattoo
☐ Lacaziosis ☐ Papilloma ☐ Pox ☐ Other Infectious ☐ Vesicular
Status: ☐ Active ☐ Inactive ☐ Active/Healing ☐ Healed ☐ NA
Color(s): ☐ Black ☐ Gray ☐ White ☐ Red ☐ Other: ______________
Shape(s): ☐ Pinhole ☐ Round ☐ Irregular ☐ Other: ______________
Site(s): ☐ Dorsal ☐ Lateral ☐ Ventral
Form(s): ☐ Flat ☐ Raised ☐ Depressed ☐ Ulcerated ☐ Cauliflower
☐ Other: ____
Biopsy: ☐ Frozen ☐ Histopath ☐ None
If biopsy taken: Vet ___________________ Size of punch _____________________

Single Lesion Only:
Size: ☐ < 1 cm^2 ☐ 1–3 cm^2 ☐ > 3 cm^2
Consistency: ☐ Firm ☐ Soft ☐ Gelatinous ☐ Slough ☐ Other: _______________

Multiple Lesions Only:
Pattern: ☐ Focal ☐ Multifocal ☐ Multifocal to Coalescing ☐ Diffuse ☐ Other
Severity: ☐ Mild (≤ 10%) ☐ Moderate (11–50%) ☐ Severe (> 50%) ☐ NA

Veterinarian ____________________________ **Date** ________________

*Data Collector*______________________________
